# Global, regional, and national incidence, prevalence, and mortality of HIV, 1980–2017, and forecasts to 2030, for 195 countries and territories: a systematic analysis for the Global Burden of Diseases, Injuries, and Risk Factors Study 2017

**DOI:** 10.1016/S2352-3018(19)30196-1

**Published:** 2019-08-19

**Authors:** GBD 2017 HIV collaborators, Tahvi D Frank, Austin Carter, Deepa Jahagirdar, Molly H Biehl, Dirk Douwes-Schultz, Samantha Leigh Larson, Megha Arora, Laura Dwyer-Lindgren, Krista M Steuben, Hedayat Abbastabar, Laith Jamal Abu-Raddad, Direslgne Misker Abyu, Maryam Adabi, Oladimeji M Adebayo, Victor Adekanmbi, Olatunji O Adetokunboh, Alireza Ahmadi, Keivan Ahmadi, Elham Ahmadian, Ehsan Ahmadpour, Muktar Beshir Ahmed, Chalachew Genet Akal, Fares Alahdab, Noore Alam, Samuel B Albertson, Birhan Tamene T Alemnew, Kefyalew Addis Alene, Vahid Alipour, Nelson Alvis-Guzman, Saeed Amini, Zohreh Anbari, Nahla Hamed Anber, Mina Anjomshoa, Carl Abelardo T Antonio, Jalal Arabloo, Olatunde Aremu, Habtamu Abera Areri, Ephrem Tsegay Asfaw, Alebachew Fasil Ashagre, Daniel Asmelash, Anemaw A Asrat, Euripide F G A Avokpaho, Ashish Awasthi, Nefsu Awoke, Martin Amogre Ayanore, Samad Azari, Alaa Badawi, Mojtaba Bagherzadeh, Maciej Banach, Aleksandra Barac, Till Winfried Bärnighausen, Sanjay Basu, Neeraj Bedi, Masoud Behzadifar, Bayu Begashaw Bekele, Saba Abraham Belay, Yared Belete Belay, Yaschilal Muche Belayneh, Adugnaw Berhane, Anusha Ganapati Bhat, Krittika Bhattacharyya, Belete Biadgo, Ali Bijani, Muhammad Shahdaat Bin Sayeed, Helen Bitew, Andrew Blinov, Kassawmar Angaw Bogale, Hunduma Amensisa Bojia, Sharath B N Burugina Nagaraja, Zahid A Butt, Lucero Cahuana-Hurtado, Julio Cesar Campuzano Rincon, Félix Carvalho, Vijay Kumar Chattu, Devasahayam J Christopher, Dinh-Toi Chu, Raquel Crider, Tukur Dahiru, Lalit Dandona, Rakhi Dandona, Ahmad Daryani, José das Neves, Jan-Walter De Neve, Louisa Degenhardt, Feleke Mekonnen Demeke, Asmamaw Bizuneh Demis, Dereje Bayissa Demissie, Gebre Teklemariam Demoz, Kebede Deribe, Don Des Jarlais, Govinda Prasad Dhungana, Daniel Diaz, Shirin Djalalinia, Huyen Phuc Do, Linh Phuong Doan, Herbert Duber, Manisha Dubey, Eleonora Dubljanin, Eyasu Ejeta Duken, Bereket Duko Adema, Andem Effiong, Aziz Eftekhari, Maysaa El Sayed Zaki, Shaimaa I El-Jaafary, Ziad El-Khatib, Aisha Elsharkawy, Aman Yesuf Endries, Sharareh Eskandarieh, Oghenowede Eyawo, Farshad Farzadfar, Batool Fatima, Netsanet Fentahun, Eduarda Fernandes, Irina Filip, Florian Fischer, Morenike Oluwatoyin Folayan, Masoud Foroutan, Takeshi Fukumoto, Nancy Fullman, Alberto L Garcia-Basteiro, Reta Tsegaye Gayesa, Ketema Bizuwork Gebremedhin, Gebreamlak Gebremedhn Gebremedhn Gebremeskel, Kelali Kalaye Gebreyohannes, Getnet Azeze Gedefaw, Belayneh K Gelaw, Hailay Abrha Gesesew, Birhanu Geta, Kebede Embaye Gezae, Keyghobad Ghadiri, Ahmad Ghashghaee, Themba T G Ginindza, Harish Chander Gugnani, Rafael Alves Guimarães, Michael Tamene Haile, Gessessew Bugssa Hailu, Arvin Haj-Mirzaian, Arya Haj-Mirzaian, Samer Hamidi, Senad Handanagic, Demelash Woldeyohannes Handiso, Lolemo Kelbiso Hanfore, Amir Hasanzadeh, Hadi Hassankhani, Hamid Yimam Hassen, Simon I Hay, Andualem Henok, Chi Linh Hoang, H Dean Hosgood, Mehdi Hosseinzadeh, Mohamed Hsairi, Segun Emmanuel Ibitoye, Bulat Idrisov, Kevin S Ikuta, Olayinka Stephen Ilesanmi, Seyed Sina Naghibi Irvani, Chinwe Juliana Iwu, Kathryn H Jacobsen, Spencer L James, Ensiyeh Jenabi, Ravi Prakash Jha, Jost B Jonas, Zahra Jorjoran Shushtari, Ali Kabir, Zubair Kabir, Rajendra Kadel, Amir Kasaeian, Belete Kassa, Getachew Mullu Kassa, Tesfaye Dessale Kassa, Gbenga A Kayode, Mihiretu M Kebede, Adane Teshome Kefale, Andre Pascal Kengne, Yousef Saleh Khader, Morteza Abdullatif Khafaie, Nauman Khalid, Ejaz Ahmad Khan, Gulfaraz Khan, Junaid Khan, Young-Ho Khang, Khaled Khatab, Salman Khazaei, Abdullah T Khoja, Aliasghar A Kiadaliri, Yun Jin Kim, Adnan Kisa, Sezer Kisa, Sonali Kochhar, Hamidreza Komaki, Parvaiz A Koul, Ai Koyanagi, Barthelemy Kuate Defo, G Anil Kumar, Manasi Kumar, Desmond Kuupiel, Dharmesh Kumar Lal, Jane Jean-Hee Lee, Tsegaye Lolaso Lenjebo, Cheru Tesema Leshargie, Erlyn Rachelle King Macarayan, Emilie R Maddison, Hassan Magdy Abd El Razek, Carlos Magis-Rodriguez, Phetole Walter Mahasha, Marek Majdan, Azeem Majeed, Reza Malekzadeh, Navid Manafi, Chabila Christopher Mapoma, Francisco Rogerlândio Martins-Melo, Anthony Masaka, Emmanuel Ngassa Laurent Mayenga, Varshil Mehta, Gebrekiros Gebremichael Meles, Hagazi Gebre Meles, Addisu Melese, Mulugeta Melku, Peter T N Memiah, Ziad A Memish, Alemayehu Toma Mena, Walter Mendoza, Desalegn Tadese Mengistu, Getnet Mengistu, Tuomo J Meretoja, Tomislav Mestrovic, Ted R Miller, Babak Moazen, Bahram Mohajer, Amjad Mohamadi-Bolbanabad, Karzan Abdulmuhsin Mohammad, Yousef Mohammad, Aso Mohammad Darwesh, Naser Mohammad Gholi Mezerji, Moslem Mohammadi, Roghayeh Mohammadibakhsh, Milad Mohammadoo-Khorasani, Jemal Abdu Mohammed, Shafiu Mohammed, Farnam Mohebi, Ali H Mokdad, Yoshan Moodley, Maryam Moossavi, Ghobad Moradi, Maziar Moradi-Lakeh, Marilita M Moschos, Tilahun Belete Mossie, Seyyed Meysam Mousavi, Kindie Fentahun Muchie, Atalay Goshu Muluneh, Moses K Muriithi, Ghulam Mustafa, Saravanan Muthupandian, Ahamarshan Jayaraman Nagarajan, Gurudatta Naik, Farid Najafi, Javad Nazari, Duduzile Edith Ndwandwe, Cuong Tat Nguyen, Huong Lan Thi Nguyen, Son Hoang Nguyen, Trang Huyen Nguyen, Dina Nur Anggraini Ningrum, Molly R Nixon, Chukwudi A Nnaji, Mehdi Noroozi, Jean Jacques Noubiap, Malihe Nourollahpour Shiadeh, Mohammed Suleiman Obsa, Emmanuel Ankrah Odame, Richard Ofori-Asenso, Felix Akpojene Ogbo, Anselm Okoro, Olanrewaju Oladimeji, Andrew T Olagunju, Tinuke O Olagunju, Solomon Olum, Kwaku Oppong Asante Oppong Asante, Eyal Oren, Stanislav S Otstavnov, Mahesh PA, Jagadish Rao Padubidri, Smita Pakhale, Amir H Pakpour, Sangram Kishor Patel, Kebreab Paulos, Veincent Christian Filipino Pepito, Emmanuel K Peprah, Bakhtiar Piroozi, Akram Pourshams, Mostafa Qorbani, Mohammad Rabiee, Navid Rabiee, Amir Radfar, Anwar Rafay, Alireza Rafiei, Fakher Rahim, Afarin Rahimi-Movaghar, Vafa Rahimi-Movaghar, Sajjad ur Rahman, Chhabi Lal Ranabhat, Salman Rawaf, Cesar Reis, Vishnu Renjith, Melese Abate Reta, Mohammad Sadegh Rezai, Carlos Miguel Rios González, Elias Merdassa Roro, Ali Rostami, Salvatore Rubino, Sahar Saeedi Moghaddam, Saeed Safari, Rajesh Sagar, Mohammad Ali Sahraian, Marwa R Rashad Salem, Yahya Salimi, Joshua A Salomon, Evanson Zondani Sambala, Abdallah M Samy, Benn Sartorius, Maheswar Satpathy, Monika Sawhney, Mehdi Sayyah, Aletta Elisabeth Schutte, Sadaf G Sepanlou, Seyedmojtaba Seyedmousavi, Hosein Shabaninejad, Amira A Shaheen, Masood Ali Shaikh, Seifadin Ahmed Shallo, Morteza Shamsizadeh, Hamid Sharifi, Kenji Shibuya, Jae Il Shin, Reza Shirkoohi, Diego Augusto Santos Silva, Dayane Gabriele Alves Silveira, Jasvinder A Singh, Malede Mequanent M Sisay, Mekonnen Sisay, Solomon Sisay, Amanda E Smith, Anton Sokhan, Ranjani Somayaji, Sergey Soshnikov, Dan J Stein, Mu'awiyyah Babale Sufiyan, Bruno F Sunguya, Bryan L Sykes, Birkneh Tilahun Tadesse, Degena Bahrey Tadesse, Koku Sisay Tamirat, Nuno Taveira, Shishay Wahdey Tekelemedhin, Habtamu Denekew Temesgen, Fisaha Haile Tesfay, Manaye Yihune Teshale, Subash Thapa, Kenean Getaneh Tlaye, Stephanie M Topp, Marcos Roberto Tovani-Palone, Bach Xuan Tran, Khanh Bao Tran, Irfan Ullah, Bhaskaran Unnikrishnan, Olalekan A Uthman, Yousef Veisani, Sergey Konstantinovitch Vladimirov, Fiseha Wadilo Wada, Yasir Waheed, Kidu Gidey Weldegwergs, Girmay Teklay T Weldesamuel, Ronny Westerman, Tissa Wijeratne, Haileab Fekadu Wolde, Dawit Zewdu Wondafrash, Tewodros Eshete Wonde, Berhanu Yazew Wondmagegn, Addisu Gize Yeshanew, Mekdes Tigistu Yilma, Ebrahim M Yimer, Naohiro Yonemoto, Marcel Yotebieng, Yoosik Youm, Chuanhua Yu, Zoubida Zaidi, Afshin Zarghi, Zerihun Menlkalew Zenebe, Taye Abuhay Zewale, Arash Ziapour, Sanjay Zodpey, Mohsen Naghavi, Stein Emil Vollset, Haidong Wang, Stephen S Lim, Hmwe Hmwe Kyu, Christopher J L Murray

## Abstract

**Background:**

Understanding the patterns of HIV/AIDS epidemics is crucial to tracking and monitoring the progress of prevention and control efforts in countries. We provide a comprehensive assessment of the levels and trends of HIV/AIDS incidence, prevalence, mortality, and coverage of antiretroviral therapy (ART) for 1980–2017 and forecast these estimates to 2030 for 195 countries and territories.

**Methods:**

We determined a modelling strategy for each country on the basis of the availability and quality of data. For countries and territories with data from population-based seroprevalence surveys or antenatal care clinics, we estimated prevalence and incidence using an open-source version of the Estimation and Projection Package—a natural history model originally developed by the UNAIDS Reference Group on Estimates, Modelling, and Projections. For countries with cause-specific vital registration data, we corrected data for garbage coding (ie, deaths coded to an intermediate, immediate, or poorly defined cause) and HIV misclassification. We developed a process of cohort incidence bias adjustment to use information on survival and deaths recorded in vital registration to back-calculate HIV incidence. For countries without any representative data on HIV, we produced incidence estimates by pulling information from observed bias in the geographical region. We used a re-coded version of the Spectrum model (a cohort component model that uses rates of disease progression and HIV mortality on and off ART) to produce age-sex-specific incidence, prevalence, and mortality, and treatment coverage results for all countries, and forecast these measures to 2030 using Spectrum with inputs that were extended on the basis of past trends in treatment scale-up and new infections.

**Findings:**

Global HIV mortality peaked in 2006 with 1·95 million deaths (95% uncertainty interval 1·87–2·04) and has since decreased to 0·95 million deaths (0·91–1·01) in 2017. New cases of HIV globally peaked in 1999 (3·16 million, 2·79–3·67) and since then have gradually decreased to 1·94 million (1·63–2·29) in 2017. These trends, along with ART scale-up, have globally resulted in increased prevalence, with 36·8 million (34·8–39·2) people living with HIV in 2017. Prevalence of HIV was highest in southern sub-Saharan Africa in 2017, and countries in the region had ART coverage ranging from 65·7% in Lesotho to 85·7% in eSwatini. Our forecasts showed that 54 countries will meet the UNAIDS target of 81% ART coverage by 2020 and 12 countries are on track to meet 90% ART coverage by 2030. Forecasted results estimate that few countries will meet the UNAIDS 2020 and 2030 mortality and incidence targets.

**Interpretation:**

Despite progress in reducing HIV-related mortality over the past decade, slow decreases in incidence, combined with the current context of stagnated funding for related interventions, mean that many countries are not on track to reach the 2020 and 2030 global targets for reduction in incidence and mortality. With a growing population of people living with HIV, it will continue to be a major threat to public health for years to come. The pace of progress needs to be hastened by continuing to expand access to ART and increasing investments in proven HIV prevention initiatives that can be scaled up to have population-level impact.

**Funding:**

Bill & Melinda Gates Foundation, National Institute of Mental Health of the US National Institutes of Health (NIH), and the National Institute on Aging of the NIH.

## Introduction

Between 2000 and 2015, excitement around the Millennium Development Goals (MDGs) galvanised more than US$500 billion in spending on prevention, care, and treatment for HIV/AIDS globally.^1^ Despite the subsequent decrease in overall HIV-related mortality, more than 36 million people still live with HIV/AIDS, which continues to be the underlying cause of death for almost 1 million people every year, concentrated in sub-Saharan Africa.^2^, ^3^ Recognising the sustained threat, UNAIDS set targets for the years 2020 and 2030 with the aim of ending the epidemic by 2030.^4^, ^5^ In this study, we estimate the current and future burden of HIV/AIDS and track progress towards meeting these targets.

**Research in context**

**Evidence before this study**

The levels and trends of the global HIV/AIDS epidemic have been estimated by two groups: the Global Burden of Diseases, Injuries, and Risk Factors Study (GBD) and UNAIDS. We searched PubMed with the search terms hiv[MeSH] AND (“mortality” OR “incidence” OR “prevalence”) AND “global” AND (trend*), with no language restrictions, for articles published since database inception until Nov 7, 2018. We did not identify any additional studies that provided comparable evaluations of the global trends in the HIV/AIDS epidemic across countries. The last GBD on HIV was in 2015; however, it did not include assessment of achieving UNAIDS targets using forecasts of past trends and associations in the data.

**Added value of this study**

For GBD 2017, the main inputs for our estimation of global HIV trends were systematically updated. These updates include a comprehensive update of population estimates that are internally consistent with fertility and mortality estimates for GBD 2017, and incorporate new prevalence data from national surveys and antenatal care clinics. Additionally, we made improvements in our estimation of paediatric HIV via modelling of natural disease progression and incorporating cohort data on child antiretroviral therapy (ART) initiation and mortality. We also better reflected geographical differences in the sex-specific distribution of HIV burden on the basis of a model fit to the sex ratio of prevalence observed in countries with representative surveys. Finally, we used forecasting methods to generate country-level estimates towards achieving global targets related to ART coverage, HIV incidence, and HIV-related mortality by 2020 and 2030.

**Implications of all the available evidence**

By improving and extending existing HIV/AIDS burden estimates, this study provides valuable insight into progress towards Sustainable Development Goal 3's target to end the AIDS epidemic by 2030 and the fast-track strategy to do so. Relative to incidence and mortality, more countries are on track to meet ART coverage targets of 81% (90% started, 90% retained) by 2020 and 90% (95% started, 95% retained) by 2030. The relative progress necessary to achieve the 2020 and 2030 targets for reduction in incidence and mortality is not on pace in most countries. Renewed attention and investment in HIV prevention initiatives could help to restore global propensity to meet these targets. This study's assessment of current trends and progress towards ambitious global targets provides evidence for decision makers to respond to current needs and plan for a future free of HIV/AIDS.

Complementing the ambitious Sustainable Development Goal (SDG) to end the HIV/AIDS epidemic by 2030, UNAIDS' 90-90-90 targets (90% of people living with HIV diagnosed, of whom 90% are on treatment, of whom 90% are virally suppressed) have been set for 2020, and 95-95-95 targets (95% of people living with HIV diagnosed, of whom 95% are on treatment, of whom 95% are virally suppressed) for 2030.^5^ In accordance with this fast-track initiative to achieve the SDG goal, UNAIDS has since set targets for reducing the number of HIV incident cases and deaths between 2010 and 2020 by 75% and between 2010 and 2030 by 90% for each country.^4^ Although these latest targets have helped to renew focus on the epidemic, measuring patterns in HIV/AIDS incidence, prevalence, and mortality is challenging, in part because of poor vital registration data and incomplete disease notification systems in high-burden areas, and complex disease modelling strategies and methodological limitations.^6^ Still, comprehensive global estimates are needed to track progress and understand future burden.

In this Article, we present results from the Global Burden of Diseases, Injuries, and Risk Factors Study (GBD) 2017. We address several methodological and data-related challenges associated with estimating HIV burden to provide a comprehensive and robust assessment of trends in HIV incidence, prevalence, and mortality across 195 countries and territories from 1980 to 2017. Building on previous iterations, we extensively updated population estimates and incorporated new prevalence data from national surveys and antenatal care clinics. Additionally, we generated country-level forecasts towards achieving targets associated with antiretroviral therapy (ART) coverage, HIV incidence, and HIV-related mortality. These forecasts enable us to report country-specific progress towards achieving the following targets: a reduction in the number of HIV incident cases of 75% between 2010 and 2020 and 90% between 2010 and 2030; a reduction in the number of HIV deaths of 75% between 2010 and 2020 and 90% between 2010 and 2030; 81% (90% started, 90% retained) ART coverage by 2020 and 90% (95% started, 95% retained) coverage by 2030.^4^, ^5^

## Methods

### Study design and modelling strategy

GBD is a systematic, scientific effort to quantify the comparative magnitude of health loss due to diseases and injuries by age, sex, and geography over time. GBD 2017 includes 195 countries and territories, 16 of which (Brazil, China, Ethiopia, India, Indonesia, Iran, Japan, Kenya, Mexico, New Zealand, Norway, Russia, South Africa, Sweden, the UK, and the USA) were analysed at the subnational level. The conceptual and analytical framework for GBD, hierarchy of causes, and detailed methods have been published elsewhere.^2^, ^3^, ^7^ The GBD protocol is also available online. Herein we describe the specific methods used for analysing the burden of HIV for GBD 2017.

Input data for modelling HIV morbidity and mortality include vital registration data, household seroprevalence surveys, data from antenatal care clinics, demographic estimates (population, fertility, migration, and HIV-free survival rates from GBD 2017), intervention coverage data reported to UNAIDS including ART, prevention of mother-to-child transmission, HIV mortality on and off ART, and rates of disease progression from a systematic review (appendix 1 pp 2–4).

The GBD framework for HIV/AIDS aims to produce internally consistent estimates for HIV incidence, prevalence, and mortality and relies on two established estimation models. We used the Estimation and Projection Package (EPP), an HIV epidemic model originally developed by the UNAIDS Reference Group on Estimates, Modelling, and Projections.^8^ EPP uses Bayesian methods to infer force of infection from trends in HIV prevalence data. EPP generates incidence and prevalence estimates for individuals aged 15–49 years for both sexes combined. We also used a modified version of Spectrum, a compartmental model used by UNAIDS that ages a population over time while applying HIV incidence, progression, and mortality to produce age-sex-specific HIV incidence, prevalence, and mortality.^8^

Multiple methodological improvements to both EPP and Spectrum were made for GBD estimation, including developing a model of ART coverage distribution as a function of income, age, sex, and disease progression that we used in Spectrum. Full details of modifications to EPP and Spectrum are in appendix 1 (pp 7–13).

To ensure appropriate modelling techniques, we grouped countries on the basis of availability and quality of data. Group 1 includes countries with HIV prevalence data from antenatal care clinics or representative population-based seroprevalence surveys. Group 1A includes countries with a peak of at least 0·5% prevalence, and group 1B includes countries with a peak prevalence of at least 0·25% plus vital registration completeness less than 65%. Group 2 includes all other countries, which are further classified as groups 2A, 2B, and 2C on the basis of availability of vital registration data. Group 2A locations have high-quality data, group 2B locations have at least some data, and group 2C locations have no data on HIV-specific mortality.^2^ The modelling framework by country grouping is shown in appendix 1 (pp 5, 6).

This study was approved by the University of Washington Institutional Review Board (application 46665).

### Incidence and prevalence estimation

For group 1 countries, we used EPP to estimate incidence and prevalence for individuals aged 15–49 years, for both sexes combined, using population-based surveys and antenatal care clinic data. To account for bias created by the differences in HIV prevalence between pregnant women who attended an antenatal care clinic and the general population, we extracted data from available Demographic and Health Surveys on HIV prevalence among pregnant women who gave birth in the past year and who attended an antenatal care clinic. For antenatal care bias adjustment, we input this data into a regression model with regional random effects to generate country-specific prior distributions where surveys were available and regional prior distributions for locations without a survey. We then used the incidence and prevalence results from EPP as inputs in Spectrum to further disaggregate to age-sex-specific HIV incidence and prevalence. We used the sex ratio of prevalence from population-based surveys to inform the sex-splitting assumptions for adults in Spectrum, and applied default age-splitting assumptions from Spectrum.^8^ We calculated vertical transmission as a function of prevention of mother-to-child transmission inputs and age-specific fertility rates adjusted to account for differential fertility among women who were HIV positive.

For group 2 countries, we developed a process called cohort incidence bias adjustment to estimate incidence and prevalence using mortality data. We ran a first stage of Spectrum to generate initial incidence, prevalence, and mortality curves, along with incidence cohort survival. We then calculated the bias between Spectrum mortality estimates and smoothed vital registration data for each year, which we used along with Spectrum cohort survival estimates to adjust incidence (appendix 1 pp 11–13). To account for sensitivity in our estimates to input incidence, we ran the first stage of Spectrum using various input incidence curves and selected the option with the smallest resulting bias in mortality estimates. We ran a second stage of Spectrum using adjusted incidence to produce age-sex-specific incidence and prevalence estimates. In countries with high-quality case notification data, we scaled incidence results to align with case reports after accounting for an assumed average of 5 years' lag between infection and diagnosis.^9^

### Mortality estimation

We undertook a meta-analysis of cohort studies to derive on-ART and off-ART mortality as inputs into Spectrum and EPP. We estimated age-sex-specific, CD4-specific, region-specific, and duration-specific on-ART mortality using cohort data after correcting for loss to follow-up (appendix 1 pp 4–7). We jointly estimated off-ART mortality and CD4 progression via an optimisation process that found a best fit to survival curves from cohort studies.

For group 1 countries, we generated age-sex-specific HIV mortality estimates in Spectrum using the incidence and prevalence estimated in EPP. For group 2 countries, we adjusted vital registration data for incompleteness and garbage coding (ie, deaths coded to an intermediate, immediate, or poorly defined cause).^2^ We further corrected the data for HIV misclassification by identifying causes of death that deviated from expected age patterns of mortality in years with known HIV epidemics, and excess deaths were attributed to HIV. We used spatiotemporal Gaussian process regression to smooth and complete the time series of adjusted vital registration data (appendix 1 p 11). For groups 2A and 2B, we used the smoothed vital registration data to inform Spectrum-estimated mortality through the cohort incidence bias adjustment process. In group 2C countries, we leveraged spatial information by sampling cohort incidence bias adjustment-generated incidence-adjustment scalars in the region, which were then input into Spectrum to create mortality estimates.

The GBD framework produced three distinct sources of HIV mortality estimates: HIV mortality results from Spectrum; estimated excess HIV mortality from the all-cause mortality process; and smoothed HIV-specific mortality from vital registration data.^10^ For group 1 countries, we used an ensemble approach to reconcile the differences between HIV mortality estimated by Spectrum and by the all-cause mortality process and generate final HIV mortality. In group 1 countries, EPP and Spectrum estimates were largely driven by HIV prevalence data and mortality estimates generated from cohort data, whereas the all-cause mortality process was primarily based on sibling survival data. For individuals aged 15 years and older, the ensemble model averaged HIV mortality estimates from the two processes with equal weights. For individuals younger than 15 years, we applied the fraction of deaths due to HIV in Spectrum to estimated all-cause mortality to generate HIV-specific mortality and mortality from all other causes (HIV-free mortality). In group 2A countries, we estimated mortality only from vital registration data, and for group 2B and 2C countries we only used Spectrum results.

### Forecasting to 2030

We forecasted HIV incidence, prevalence, mortality, and treatment coverage through to 2030 in Spectrum using input parameters extended to 2030. We forecasted the adult ART coverage input on the basis of forecasted ART price, HIV spending on care and treatment, and lag-distributed income (ie, gross domestic product per capita that has been smoothed over the preceding 10 years). We modelled country-year-specific annual ART price per patient using Gaussian process regression with data from the Global Price Reporting Mechanism.^11^ We calculated the annualised rate of change of per-capita expenditure on HIV care and treatment in each country since 2010. We then forecast expenditure on HIV care and treatment for each country using the 50th percentile annualised rate of change across countries.^1^ We calculated annual dose-equivalents of ART by dividing spending by ART price, and we used logistic regression to model the association between annual dose-equivalents and ART coverage. We forecasted other treatment coverage inputs to Spectrum, such as child ART coverage and prevention of mother-to-child transmission, using the same approach based on the 50th percentile annualised rate of change observed across countries. Forecasting the incidence input had two steps. First, we calculated counterfactual incidence (ie, expected incidence in the absence of ART) using an assumption of 70% viral suppression among those on treatment,^12^ then we forecast counterfactual incidence using the 50th percentile annualised rate of change observed across countries in the previous 5 years. Because the forecasted incidence was derived from the counterfactual incidence using forecasted ART coverage, the final forecasted incidence changed in response to both the underlying secular trend and improvements in ART coverage. We used forecasted demographic inputs that were estimated for each location,^13^ then we ran Spectrum for all locations. Full details on the methods for forecasting are in appendix 1 (pp 17–22). We used the mean values (rounded to the nearest integer) of the resultant HIV forecasts to determine whether countries were on track to meet the 2020 and 2030 UNAIDS targets.

### Uncertainty analysis

We propagated uncertainty by generating 1000 draws of key inputs, including draw-level linkage of HIV-free mortality with the GBD all-cause mortality process. We ran EPP and Spectrum 1000 times per location to generate results for each draw. We present results with 95% uncertainty intervals (UIs).

### Role of the funding source

The funder of the study had no role in study design, data collection, data analysis, data interpretation, or the writing of the report. All authors had full access to the data in the study and had final responsibility for the decision to submit for publication

## Results

**Figure 1:**
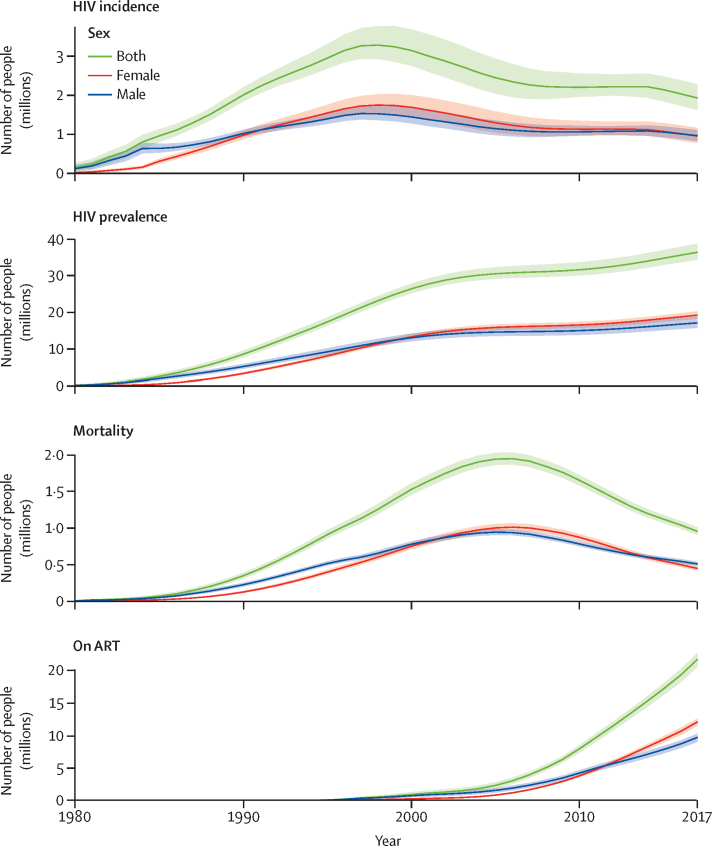
Global HIV incidence, prevalence, mortality, and people on ART, by sex, for all ages, 1980–2017 Shaded areas are 95% uncertainty intervals. ART=antiretroviral therapy.

Global deaths from HIV peaked in 2006 and have since decreased from 1·95 million (95% UI 1·87–2·04) deaths in 2006 to 0·95 million (0·91–1·01) in 2017 (figure 1). Global ART coverage increased from 2·98 million (2·44–3·58) in 2006 to 21·8 million (20·7–22·9) in 2017. The number of new HIV infection cases peaked in 1999 (3·16 million [2·79–3·67]) and has gradually decreased thereafter. Between 2007 and 2017, the global age-standardised annualised rate of change in HIV incidence decreased by 3·0% (1·5–4·5), with the number of new cases decreasing from 2·35 million (2·02–2·76) in 2007 to 1·94 million (1·63–2·29) in 2017 (table, figure 1). The confluence of these trends produces a steady increase in the total number of people living with HIV. Prevalence has increased from 8·74 million (7·90–9·68) people living with HIV in 1990 to 36·8 million (34·8–39·2) in 2017, of whom 40·5% (37·8–43·7) were not on ART.

**Table:** Number of HIV incident cases and deaths in 2017 by sex and annualised rate of change of HIV incident cases and deaths for 1990–2007 and 2007–17, for 21 GBD regions and 195 countries and territories

		**New HIV infections, 2017**			**HIV deaths, 2017**			**Annualised rate of change in new infections**		**Annualised rate of change in HIV deaths**	
		Females	Males	Total	Females	Males	Total	1990–2007	2007–17	1990–2007	2007–17
**Global**		**966 000 (786 000 to 1 180 000)**	**976 000 (835 000 to 1 120 000)**	**1 940 000 (1 630 000 to 2 290 000)**	**446 000 (417 000 to 479 000)**	**508 000 (483 000 to 540 000)**	**954 000 (907 000 to 1 010 000)**	**−0·4% (−1·2 to 0·3)**	**−3·0% (−4·5 to −1·5)**	**8·5% (7·8 to 9·1)**	**−8·3% (−8·7 to −7·9)**
Low SDI		259 000 (176 000 to 378 000)	177 000 (124 000 to 257 000)	436 000 (303 000 to 627 000)	132 000 (120 000 to 147 000)	131 000 (121 000 to 143 000)	262 000 (244 000 to 286 000)	−5·2% (−6·2 to −4·2)	−5·9% (−9·4 to −2·0)	4·6% (3·6 to 5·7)	−12·4% (−13·0 to −11·8)
Low-middle SDI		359 000 (271 000 to 459 000)	278 000 (212 000 to 356 000)	636 000 (487 000 to 808 000)	192 000 (170 000 to 217 000)	184 000 (163 000 to 207 000)	375 000 (338 000 to 416 000)	−1·6% (−2·8 to −0·5)	−4·1% (−6·1 to −2·0)	11·3% (10·0 to 12·4)	−8·5% (−9·1 to −7·8)
Middle SDI		240 000 (196 000 to 287 000)	280 000 (245 000 to 317 000)	521 000 (450 000 to 591 000)	105 000 (94 500 to 119 000)	153 000 (143 000 to 165 000)	258 000 (241 000 to 278 000)	6·0% (4·0 to 8·2)	−3·7% (−5·3 to −2·1)	18·6% (17·9 to 19·3)	−7·8% (−8·5 to −7·2)
High-middle SDI		80 800 (69 100 to 99 400)	167 000 (139 000 to 198 000)	247 000 (210 000 to 297 000)	14 200 (13 700 to 14 800)	31 500 (30 800 to 32 700)	45 700 (44 600 to 47 500)	3·7% (3·2 to 4·3)	7·5% (5·6 to 9·2)	4·6% (4·5 to 4·8)	0·7% (0·5 to 1·1)
High SDI		26 700 (15 300 to 39 200)	73 100 (42 900 to 107 000)	99 800 (58 300 to 146 000)	3170 (3140 to 3200)	8700 (8610 to 8800)	11 900 (11 800 to 12 000)	−2·5% (−3·9 to −1·0)	1·9% (−0·8 to 3·2)	−5·7% (−5·8 to −5·7)	−5·3% (−5·4 to −5·2)
**Central Europe, eastern Europe, and central Asia**		**59 300 (49 400 to 74 000)**	**121 000 (94 800 to 149 000)**	**180 000 (146 000 to 222 000)**	**8260 (8170 to 8350)**	**18 700 (18 400 to 18 900)**	**26 900 (26 700 to 27 100)**	**8·1% (7·0 to 9·2)**	**11·7% (9·1 to 13·5)**	**7·7% (7·6 to 7·8)**	**1·8% (1·7 to 2·0)**
Central Asia		3280 (2200 to 4480)	4030 (2950 to 5110)	7300 (5200 to 9550)	398 (377 to 419)	902 (869 to 935)	1300 (1260 to 1340)	4·2% (2·2 to 6·3)	7·2% (3·7 to 9·8)	6·8% (6·6 to 7·2)	−0·6% (−1·0 to −0·2)
	Armenia	65·3 (46·1 to 91·6)	99·6 (67·6 to 154)	165 (120 to 241)	5·7 (5·2 to 6·2)	18·0 (17·1 to 19·0)	23·7 (22·7 to 24·8)	21·1% (11·6 to 59·7)	7·5% (4·1 to 12·7)	11·7% (11·3 to 12·0)	10·7% (10·0 to 11·3)
	Azerbaijan	91·2 (60·5 to 127)	260 (157 to 416)	351 (222 to 535)	7·8 (5·5 to 9·8)	23·7 (17·9 to 28·3)	31·5 (23·8 to 37·8)	2·4% (−5·8 to 5·8)	9·5% (5·2 to 12·9)	6·4% (3·5 to 14·8)	−8·0% (−11·2 to −5·0)
	Georgia	108 (74·0 to 160)	283 (181 to 491)	391 (256 to 641)	8·5 (7·9 to 9·1)	25·9 (24·5 to 27·4)	34·4 (32·9 to 35·9)	18·4% (12·8 to 25·9)	2·2% (−0·0 to 4·4)	8·7% (8·3 to 9·1)	17·6% (17·0 to 18·3)
	Kazakhstan	988 (640 to 1440)	1510 (1000 to 1880)	2500 (1670 to 3220)	72·6 (67·2 to 78·2)	186 (175 to 198)	258 (246 to 271)	2·5% (0·9 to 4·2)	12·1% (8·0 to 14·2)	7·8% (7·5 to 8·2)	−2·1% (−2·7 to −1·6)
	Kyrgyzstan	457 (291 to 719)	447 (275 to 707)	904 (575 to 1390)	53·9 (50·2 to 57·6)	133 (126 to 140)	187 (179 to 194)	8·9% (7·3 to 11·1)	6·2% (1·1 to 10·7)	9·2% (8·8 to 9·5)	1·6% (1·0 to 2·2)
	Mongolia	11·5 (2·3 to 29·5)	48·7 (11·3 to 105)	60·2 (14·9 to 132)	3·8 (0·3 to 11·1)	16·1 (1·6 to 32·9)	19·9 (2·1 to 44·0)	25·1% (47·5 to 58·1)	6·7% (0·2 to 11·6)	35·4% (43·1 to 49·1)	8·2% (−11·0 to 13·4)
	Tajikistan	419 (184 to 633)	156 (95·4 to 224)	575 (280 to 819)	29·8 (21·3 to 44·2)	14·7 (12·2 to 18·1)	44·5 (34·3 to 60·5)	2·4% (−0·6 to 4·5)	9·1% (2·4 to 13·3)	14·1% (11·0 to 18·8)	−13·5% (−16·5 to −9·5)
	Turkmenistan	44·6 (32·4 to 69·4)	116 (88·5 to 169)	160 (122 to 236)	26·4 (24·4 to 28·8)	73·0 (68·7 to 77·6)	99·5 (94·6 to 105)	−6·4% (−10·1 to −1·7)	2·2% (0·3 to 4·4)	2·5% (2·2 to 2·9)	−2·5% (−3·1 to −1·8)
	Uzbekistan	1090 (330 to 1910)	1100 (305 to 2000)	2200 (650 to 3780)	189 (174 to 204)	413 (390 to 436)	601 (575 to 628)	5·0% (0·8 to 11·4)	5·1% (−7·3 to 11·6)	5·1% (4·8 to 5·4)	1·0% (0·3 to 1·5)
Central Europe		529 (401 to 669)	1600 (1260 to 2120)	2130 (1670 to 2770)	126 (118 to 144)	395 (356 to 462)	521 (476 to 604)	6·3% (4·4 to 8·0)	−0·1% (−2·1 to 1·4)	−0·0% (−0·4 to 0·8)	−0·9% (−1·7 to −0·1)
	Albania	0·9 (0·7 to 1·4)	2·1 (1·5 to 3·0)	3·0 (2·2 to 4·3)	0·4 (0·4 to 0·5)	0·9 (0·8 to 1·0)	1·3 (1·1 to 1·5)	0·4% (−0·4 to 1·3)	2·0% (0·2 to 3·7)	2·9% (2·0 to 4·2)	−3·6% (−4·8 to −2·4)
	Bosnia and Herzegovina	1·2 (0·8 to 1·6)	2·2 (1·6 to 3·2)	3·4 (2·5 to 4·4)	0·5 (0·5 to 0·6)	1·0 (0·9 to 1·2)	1·5 (1·4 to 1·7)	−3·9% (−5·1 to −2·5)	4·5% (2·6 to 6·1)	−2·0% (−3·4 to 0·0)	−3·9% (−4·7 to −3·1)
	Bulgaria	28·1 (22·9 to 33·9)	100 (66·9 to 126)	128 (93·7 to 156)	12·5 (11·8 to 13·3)	42·1 (39·9 to 44·3)	54·6 (52·3 to 57·0)	1·5% (−2·5 to 56·2)	3·3% (1·2 to 4·8)	6·7% (6·4 to 7·0)	−4·7% (−5·2 to −4·2)
	Croatia	10·9 (5·5 to 15·1)	34·1 (17·9 to 48·0)	44·9 (23·6 to 61·9)	2·1 (2·0 to 2·3)	6·8 (6·5 to 7·2)	9·0 (8·7 to 9·3)	1·7% (0·0 to 3·2)	1·9% (−3·9 to 6·0)	−2·6% (−2·9 to −2·2)	6·7% (6·2 to 7·3)
	Czech Republic	17·6 (9·3 to 28·4)	87·8 (38·0 to 148)	105 (48·0 to 165)	7·4 (6·9 to 7·9)	16·4 (15·6 to 17·2)	23·7 (22·9 to 24·6)	9·2% (8·0 to 10·3)	2·8% (−3·7 to 5·6)	3·9% (3·6 to 4·2)	5·4% (4·9 to 5·9)
	Hungary	28·8 (17·5 to 50·9)	99·4 (63·4 to 158)	128 (86·0 to 193)	8·1 (7·5 to 8·7)	31·8 (30·2 to 33·7)	39·8 (38·1 to 41·7)	−1·5% (−3·0 to 0·2)	3·1% (1·2 to 5·6)	−4·2% (−4·5 to −3·8)	−7·6% (−8·2 to −7·0)
	Macedonia	4·0 (3·0 to 5·2)	5·4 (4·0 to 7·4)	9·3 (7·1 to 12·2)	0·7 (0·5 to 0·9)	1·0 (0·8 to 1·1)	1·7 (1·4 to 2·0)	9·9% (7·6 to 11·8)	1·8% (−0·7 to 4·0)	8·6% (7·3 to 10·1)	−2·2% (−4·2 to −0·9)
	Montenegro	1·9 (1·4 to 2·4)	4·7 (3·6 to 6·2)	6·6 (5·1 to 8·4)	0·6 (0·4 to 0·9)	0·7 (0·6 to 0·8)	1·4 (1·1 to 1·7)	3·9% (1·8 to 5·7)	3·1% (1·0 to 4·8)	4·9% (3·2 to 6·3)	−2·8% (−6·1 to −0·0)
	Poland	151 (78·7 to 246)	532 (239 to 981)	683 (319 to 1220)	32·0 (30·2 to 33·9)	112 (106 to 118)	144 (138 to 150)	5·2% (2·8 to 7·6)	0·3% (−5·6 to 2·7)	5·3% (5·0 to 5·6)	−2·2% (−2·7 to −1·6)
	Romania	258 (185 to 323)	591 (417 to 778)	849 (634 to 1050)	47·9 (45·1 to 51·2)	118 (112 to 123)	165 (160 to 171)	10·0% (7·6 to 12·6)	−0·1% (−2·4 to 1·7)	−2·8% (−3·2 to −2·4)	2·7% (2·1 to 3·2)
	Serbia	18·5 (11·6 to 30·9)	112 (73·2 to 181)	130 (85·9 to 209)	12·3 (6·2 to 28·9)	60·2 (23·9 to 126)	72·5 (30·3 to 156)	8·3% (4·6 to 60·9)	−4·5% (−7·3 to −1·6)	3·7% (−0·3 to 54·4)	1·9% (−4·5 to 6·9)
	Slovakia	6·5 (4·7 to 8·3)	16·5 (11·2 to 25·2)	23·0 (16·2 to 33·5)	1·2 (0·8 to 1·7)	2·7 (2·1 to 3·3)	3·9 (3·0 to 4·9)	4·2% (−1·5 to 48·2)	2·0% (−0·1 to 4·3)	7·7% (4·1 to 38·6)	−0·1% (−3·7 to 2·0)
	Slovenia	1·6 (1·1 to 2·5)	9·9 (6·7 to 15·2)	11·5 (7·9 to 17·8)	0·4 (0·3 to 0·4)	2·1 (2·0 to 2·2)	2·5 (2·4 to 2·6)	−1·2% (−4·7 to 48·3)	4·1% (2·3 to 5·8)	−1·8% (−2·1 to −1·4)	−4·8% (−5·3 to −4·2)
Eastern Europe		55 500 (46 300 to 68 700)	115 000 (90 500 to 142 000)	171 000 (139 000 to 209 000)	7730 (7650 to 7820)	17 400 (17 100 to 17 600)	25 100 (24 800 to 25 300)	8·8% (7·6 to 10·1)	12·4% (9·8 to 14·3)	8·4% (8·3 to 8·5)	2·1% (2·0 to 2·3)
	Belarus	1280 (781 to 1930)	1260 (935 to 1680)	2540 (1730 to 3610)	94·1 (89·0 to 99·4)	210 (194 to 226)	304 (288 to 320)	7·8% (0·9 to 66·8)	9·8% (6·8 to 12·6)	10·6% (10·1 to 11·0)	0·3% (−0·4 to 1·0)
	Estonia	41·6 (30·5 to 61·3)	165 (119 to 238)	207 (152 to 298)	7·8 (7·4 to 8·2)	29·4 (27·3 to 31·7)	37·2 (35·0 to 39·5)	27·4% (24·5 to 30·8)	−1·8% (−5·3 to 2·9)	25·5% (25·1 to 25·9)	0·2% (−0·6 to 1·1)
	Latvia	203 (134 to 238)	255 (204 to 313)	458 (345 to 528)	57·1 (54·4 to 59·7)	69·4 (64·8 to 74·0)	126 (121 to 132)	4·0% (2·9 to 4·9)	7·1% (3·8 to 8·5)	5·5% (5·1 to 5·9)	5·1% (4·5 to 5·8)
	Lithuania	41·1 (10·0 to 61·9)	94·0 (26·1 to 146)	135 (36·0 to 196)	42·3 (40·3 to 44·6)	24·7 (23·1 to 26·4)	67·0 (64·5 to 69·7)	6·4% (4·3 to 62·5)	−0·1% (−12·3 to 2·7)	9·2% (8·9 to 9·6)	0·6% (0·0 to 1·2)
	Moldova	377 (267 to 548)	398 (287 to 560)	775 (569 to 1080)	49·5 (46·8 to 52·1)	124 (116 to 132)	173 (164 to 182)	8·5% (0·9 to 14·6)	1·8% (−0·5 to 3·6)	10·7% (10·2 to 11·1)	−3·1% (−3·9 to −2·4)
	Russia	41 700 (34 600 to 51 600)	97 100 (78 500 to 122 000)	139 000 (115 000 to 171 000)	5480 (5430 to 5520)	13 800 (13 700 to 14 000)	19 300 (19 100 to 19 500)	10·0% (8·8 to 12·3)	13·2% (10·3 to 15·5)	7·0% (6·9 to 7·0)	5·9% (5·8 to 6·0)
	Ukraine	11 800 (7920 to 16 500)	16 100 (9210 to 20 500)	27 900 (19 000 to 35 300)	2000 (1930 to 2080)	3070 (2930 to 3220)	5080 (4920 to 5240)	5·9% (2·8 to 8·1)	9·7% (6·9 to 12·6)	10·7% (10·5 to 11·0)	−5·9% (−6·3 to −5·5)
**High-income**		**34 600 (19 800 to 51 000)**	**88 600 (52 700 to 130 000)**	**123 000 (72 600 to 179 000)**	**3750 (3720 to 3790)**	**10 200 (10 100 to 10 300)**	**13 900 (13 800 to 14 000)**	**−1·8% (−3·1 to −0·4)**	**2·0% (−0·2 to 3·2)**	**−5·0% (−5·1 to −5·0)**	**−4·9% (−4·9 to −4·8)**
Australasia		417 (228 to 745)	1160 (691 to 1760)	1580 (950 to 2270)	20·6 (19·8 to 21·4)	65·9 (63·8 to 68·1)	86·6 (84·4 to 88·9)	−0·3% (−2·8 to 1·9)	2·5% (1·2 to 4·0)	−8·4% (−8·7 to −8·2)	−5·9% (−6·2 to −5·6)
	Australia	378 (202 to 698)	1020 (591 to 1560)	1390 (834 to 2030)	18·7 (17·9 to 19·5)	57·9 (55·8 to 60·0)	76·6 (74·4 to 78·9)	−1·0% (−3·7 to 1·4)	2·8% (1·5 to 4·3)	−8·2% (−8·4 to −7·9)	−6·3% (−6·7 to −6·0)
	New Zealand	38·9 (20·7 to 59·8)	147 (68·4 to 233)	186 (89·6 to 293)	1·9 (1·9 to 2·0)	8·1 (7·8 to 8·3)	10·0 (9·7 to 10·3)	5·7% (4·1 to 8·9)	0·6% (−4·2 to 3·7)	−10·4% (−10·7 to −10·2)	−2·6% (−3·0 to −2·2)
High-income Asia Pacific		669 (396 to 955)	2310 (1240 to 3660)	2970 (1680 to 4530)	79·9 (78·1 to 81·9)	312 (302 to 322)	392 (382 to 402)	5·0% (2·4 to 8·5)	0·2% (−4·5 to 2·7)	4·5% (4·3 to 4·7)	−2·2% (−2·5 to −1·9)
	Brunei	7·9 (4·3 to 12·8)	27·8 (15·1 to 45·9)	35·7 (19·8 to 57·7)	1·4 (1·1 to 1·6)	3·3 (2·8 to 3·9)	4·7 (4·0 to 5·4)	5·3% (3·9 to 6·6)	3·7% (0·4 to 5·8)	6·4% (5·0 to 8·0)	−1·6% (−3·9 to 0·3)
	Japan	426 (212 to 624)	1040 (501 to 1510)	1460 (724 to 2130)	59·4 (58·1 to 60·6)	147 (144 to 151)	207 (203 to 210)	4·4% (2·8 to 6·1)	1·9% (−2·1 to 3·5)	5·8% (5·8 to 6·0)	−2·9% (−3·0 to −2·7)
	Singapore	56·0 (22·3 to 90·6)	113 (62·9 to 196)	169 (90·1 to 268)	3·1 (2·8 to 3·5)	29·6 (27·5 to 31·7)	32·7 (30·6 to 34·9)	−5·6% (−8·1 to −4·0)	−1·1% (−3·8 to 1·6)	7·5% (6·9 to 8·1)	−6·7% (−7·7 to −5·8)
	South Korea	180 (50·8 to 296)	1130 (273 to 2280)	1310 (327 to 2530)	16·1 (14·9 to 17·4)	131 (122 to 142)	148 (138 to 157)	8·3% (1·7 to 62·6)	−2·6% (−13·6 to 2·2)	1·0% (0·5 to 1·4)	−0·7% (−1·5 to 0·1)
High-income North America		15 600 (6270 to 24 700)	39 200 (16 100 to 62 300)	54 800 (22 600 to 86 200)	2140 (2110 to 2170)	5480 (5400 to 5550)	7620 (7540 to 7690)	−2·3% (−3·8 to 0·9)	1·6% (−4·7 to 3·6)	−6·2% (−6·2 to −6·1)	−6·0% (−6·2 to −5·9)
	Canada	942 (434 to 1490)	2590 (1050 to 4310)	3530 (1520 to 5660)	72·2 (68·5 to 76·3)	202 (189 to 215)	274 (261 to 287)	−1·8% (−3·5 to 1·9)	3·9% (−1·7 to 6·8)	−4·8% (−5·1 to −4·6)	−5·6% (−6·2 to −5·0)
	Greenland	6·2 (2·2 to 11·1)	4·7 (2·1 to 7·7)	10·9 (4·6 to 18·1)	0·8 (0·6 to 1·0)	0·9 (0·6 to 1·1)	1·6 (1·3 to 2·0)	−0·8% (−2·5 to 2·2)	4·2% (−2·2 to 6·6)	0·1% (−1·3 to 1·7)	−5·0% (−7·6 to −3·4)
	USA	14 700 (5440 to 23 600)	36 600 (14 300 to 58 900)	51 300 (19 700 to 81 300)	2070 (2040 to 2100)	5270 (5200 to 5340)	7340 (7260 to 7420)	−2·3% (−3·9 to 1·0)	1·5% (−5·6 to 3·6)	−6·2% (−6·3 to −6·2)	−6·0% (−6·2 to −5·9)
Southern Latin America		6630 (3020 to 12 300)	10 500 (5830 to 17 100)	17 100 (9040 to 28 300)	673 (657 to 690)	1730 (1700 to 1770)	2400 (2370 to 2440)	1·7% (0·3 to 3·2)	1·8% (0·8 to 3·0)	5·0% (4·9 to 5·1)	−0·9% (−1·1 to −0·7)
	Argentina	5750 (2490 to 11 300)	6430 (3330 to 10 900)	12 200 (6000 to 21 000)	548 (533 to 565)	1170 (1140 to 1200)	1720 (1680 to 1750)	1·0% (−0·6 to 2·6)	1·0% (−0·3 to 2·7)	4·7% (4·6 to 4·8)	−1·4% (−1·7 to −1·2)
	Chile	682 (339 to 1180)	3440 (1850 to 5490)	4120 (2220 to 6690)	85·3 (82·9 to 87·7)	419 (407 to 430)	505 (493 to 516)	5·0% (3·8 to 6·2)	3·7% (2·8 to 4·7)	6·3% (6·1 to 6·5)	0·6% (0·3 to 0·9)
	Uruguay	193 (131 to 279)	635 (527 to 781)	828 (671 to 1040)	39·8 (38·8 to 40·8)	141 (137 to 144)	181 (177 to 184)	0·4% (−6·0 to 4·8)	5·9% (3·5 to 8·5)	7·1% (7·0 to 7·3)	−0·5% (−0·8 to −0·2)
Western Europe		11 200 (6590 to 16 200)	35 500 (21 200 to 51 600)	46 700 (27 800 to 67 200)	836 (818 to 856)	2610 (2550 to 2670)	3450 (3390 to 3510)	−2·7% (−4·8 to −1·1)	2·0% (0·2 to 3·4)	−4·9% (−5·0 to −4·8)	−5·4% (−5·6 to −5·2)
	Andorra	0·6 (0·0 to 2·8)	4·0 (0·2 to 19·1)	4·6 (0·3 to 21·4)	0·1 (0·0 to 0·4)	0·3 (0·0 to 1·5)	0·4 (0·0 to 1·9)	−0·6% (−11·1 to 7·0)	0·4% (−6·3 to 7·3)	−3·6% (−10·2 to 1·4)	−8·5% (−12·8 to −3·2)
	Austria	169 (93·4 to 248)	518 (273 to 798)	688 (369 to 1030)	8·5 (8·0 to 9·0)	29·5 (27·9 to 31·2)	38·0 (36·3 to 39·8)	−0·0% (−6·1 to 65·4)	0·8% (−2·0 to 4·5)	−2·0% (−2·4 to −1·6)	−4·7% (−5·4 to −4·1)
	Belgium	350 (153 to 541)	787 (298 to 1310)	1140 (444 to 1850)	20·0 (19·0 to 21·2)	41·8 (39·7 to 44·3)	61·9 (59·4 to 64·7)	1·6% (−0·9 to 3·6)	−0·2% (−6·0 to 3·4)	−4·3% (−4·6 to −4·0)	−2·5% (−3·0 to −1·9)
	Cyprus	15·2 (10·4 to 21·0)	96·0 (68·3 to 138)	111 (79·5 to 158)	0·4 (0·4 to 0·5)	1·4 (1·2 to 1·7)	1·9 (1·6 to 2·1)	6·1% (3·6 to 9·2)	5·0% (2·7 to 7·6)	4·4% (2·5 to 6·0)	−3·7% (−5·2 to −2·1)
	Denmark	155 (53·8 to 264)	253 (125 to 457)	408 (206 to 643)	7·7 (7·2 to 8·2)	19·2 (18·1 to 20·2)	26·9 (25·8 to 28·1)	−2·3% (−4·0 to −0·8)	1·1% (−5·0 to 3·8)	−5·9% (−6·3 to −5·5)	−3·4% (−4·1 to −2·9)
	Finland	34·5 (9·7 to 59·1)	127 (34·0 to 223)	161 (43·7 to 280)	1·7 (1·6 to 1·8)	4·8 (4·5 to 5·1)	6·5 (6·2 to 6·8)	2·6% (0·5 to 4·7)	−1·2% (−10·7 to 4·0)	−0·2% (−0·6 to 0·1)	−10·2% (−10·8 to −9·6)
	France	1620 (734 to 2880)	3570 (1660 to 6480)	5190 (2670 to 8610)	134 (126 to 142)	358 (337 to 380)	491 (469 to 514)	−3·1% (−6·1 to −1·1)	0·3% (−1·6 to 2·0)	−8·7% (−8·9 to −8·5)	−6·1% (−6·7 to −5·6)
	Germany	1460 (884 to 2050)	4980 (2920 to 7180)	6430 (3810 to 9130)	102 (96·3 to 107)	363 (345 to 381)	465 (446 to 483)	1·3% (−0·2 to 2·2)	2·7% (−0·2 to 6·7)	−7·0% (−7·2 to −6·7)	−2·5% (−3·0 to −2·1)
	Greece	54·8 (35·3 to 75·5)	459 (280 to 805)	514 (326 to 854)	6·0 (5·6 to 6·4)	23·6 (22·3 to 24·8)	29·6 (28·2 to 30·9)	1·9% (−0·1 to 4·0)	0·1% (−2·1 to 2·6)	−4·0% (−4·4 to −3·6)	3·3% (2·7 to 3·9)
	Iceland	0·0 (0·0 to 0·1)	20·1 (8·9 to 30·3)	20·1 (8·9 to 30·3)	0·0 (0·0 to 0·0)	0·8 (0·8 to 0·9)	0·9 (0·8 to 0·9)	−0·1% (−2·5 to 1·9)	−1·2% (−8·5 to 3·0)	−4·3% (−4·8 to −4·0)	−4·4% (−5·2 to −3·7)
	Ireland	148 (56·6 to 225)	414 (153 to 665)	562 (206 to 884)	2·7 (2·5 to 2·9)	7·1 (6·7 to 7·6)	9·8 (9·3 to 10·3)	−0·9% (−2·8 to 0·9)	4·1% (−4·3 to 6·8)	−0·5% (−0·9 to −0·1)	−5·4% (−6·0 to −4·7)
	Israel	202 (106 to 288)	485 (253 to 695)	688 (377 to 970)	12·8 (12·2 to 13·5)	28·6 (27·1 to 30·1)	41·4 (39·8 to 43·0)	3·1% (0·6 to 5·8)	0·0% (−4·8 to 4·8)	−1·2% (−1·5 to −0·9)	−4·7% (−5·3 to −4·2)
	Italy	1580 (962 to 2300)	5560 (3360 to 8330)	7140 (4260 to 10 600)	156 (146 to 166)	505 (479 to 533)	661 (634 to 692)	−5·5% (−11·6 to 2·7)	3·7% (0·8 to 7·0)	−5·3% (−5·6 to −5·1)	−5·1% (−5·6 to −4·6)
	Luxembourg	19·7 (9·6 to 28·9)	75·9 (31·9 to 115)	95·6 (41·5 to 141)	0·6 (0·6 to 0·7)	1·8 (1·7 to 1·9)	2·4 (2·3 to 2·5)	−3·7% (−5·6 to −2·3)	2·5% (−4·8 to 5·9)	−4·8% (−5·2 to −4·3)	−4·8% (−5·5 to −4·2)
	Malta	16·7 (9·1 to 23·9)	81·7 (43·7 to 133)	98·4 (53·3 to 155)	0·4 (0·4 to 0·5)	1·2 (1·1 to 1·3)	1·6 (1·6 to 1·7)	3·7% (1·6 to 5·1)	9·1% (7·7 to 10·2)	−2·1% (−2·5 to −1·8)	−2·7% (−3·4 to −2·0)
	Netherlands	187 (95·1 to 283)	344 (166 to 526)	531 (260 to 804)	17·1 (16·1 to 18·1)	38·5 (36·5 to 40·7)	55·5 (53·3 to 58·0)	3·9% (2·5 to 5·2)	−7·3% (−13·2 to −3·3)	−8·5% (−8·8 to −8·2)	−4·7% (−5·3 to −4·2)
	Norway	95·3 (50·8 to 141)	197 (99·0 to 300)	292 (150 to 441)	4·3 (4·2 to 4·4)	10·5 (10·2 to 10·7)	14·8 (14·5 to 15·1)	−1·0% (−3·4 to 0·9)	0·7% (−2·8 to 3·6)	−5·2% (−5·4 to −5·0)	−1·0% (−1·3 to −0·8)
	Portugal	1550 (776 to 2320)	6080 (2930 to 9150)	7630 (3730 to 11 200)	108 (102 to 115)	394 (372 to 418)	502 (479 to 528)	2·5% (−0·5 to 5·3)	2·5% (−2·1 to 6·1)	7·9% (7·6 to 8·2)	−6·2% (−6·8 to −5·7)
	Spain	1150 (464 to 2120)	5380 (2160 to 9960)	6530 (2820 to 11 800)	146 (136 to 157)	563 (531 to 598)	708 (675 to 743)	−10·3% (−12·1 to −8·6)	5·8% (1·8 to 8·4)	−4·7% (−5·0 to −4·5)	−7·3% (−7·9 to −6·8)
	Sweden	243 (109 to 379)	383 (185 to 593)	626 (305 to 962)	8·2 (7·8 to 8·6)	14·6 (14·0 to 15·1)	22·8 (22·1 to 23·4)	−5·1% (−7·3 to −3·5)	1·5% (−3·9 to 3·6)	−4·6% (−4·8 to −4·3)	−5·3% (−5·7 to −5·0)
	Switzerland	194 (74·5 to 288)	460 (152 to 704)	654 (226 to 987)	10·5 (9·9 to 11·1)	27·9 (26·4 to 29·6)	38·3 (36·7 to 40·2)	−7·0% (−9·3 to −4·0)	−0·1% (−6·7 to 2·1)	−7·0% (−7·4 to −6·6)	−6·4% (−7·0 to −5·8)
	UK	1950 (1120 to 2800)	5200 (3010 to 7410)	7150 (4110 to 10 100)	89·2 (88·5 to 89·9)	175 (174 to 176)	264 (263 to 266)	3·7% (1·7 to 5·1)	1·1% (−0·8 to 2·5)	−2·4% (−2·5 to −2·4)	−3·9% (−3·9 to −3·8)
**Latin America and Caribbean**		**52 200 (44 900 to 60 600)**	**110 000 (94 300 to 129 000)**	**162 000 (140 000 to 188 000)**	**14 100 (12 800 to 16 400)**	**28 400 (26 700 to 31 100)**	**42 500 (39 800 to 47 300)**	**−0·5% (−1·8 to 0·6)**	**2·1% (0·6 to 3·2)**	**4·1% (3·3 to 4·5)**	**−2·9% (−3·4 to −2·1)**
Andean Latin America		3950 (2520 to 6980)	8440 (5850 to 13 200)	12 400 (8420 to 19 800)	1600 (803 to 3610)	3060 (1830 to 5640)	4660 (2630 to 9190)	5·6% (1·5 to 8·4)	2·0% (−0·2 to 4·5)	6·6% (3·1 to 9·6)	0·9% (−2·8 to 4·6)
	Bolivia	905 (77·5 to 3880)	1590 (168 to 5750)	2490 (260 to 9880)	458 (11·3 to 2490)	709 (31·3 to 3220)	1170 (46·3 to 5690)	0·2% (−10·4 to 8·7)	2·3% (−4·8 to 7·0)	4·0% (−4·3 to 15·5)	0·5% (−20·2 to 7·9)
	Ecuador	1050 (708 to 1410)	2060 (1740 to 2580)	3100 (2500 to 3980)	271 (252 to 293)	822 (775 to 874)	1090 (1040 to 1150)	9·2% (4·5 to 12·0)	1·4% (0·2 to 2·6)	11·9% (11·6 to 12·2)	0·4% (−0·2 to 1·0)
	Peru	1990 (1390 to 3030)	4790 (3320 to 7700)	6780 (4730 to 10 600)	868 (430 to 1320)	1530 (830 to 2370)	2400 (1270 to 3710)	7·1% (5·1 to 8·4)	2·0% (−0·9 to 5·7)	6·4% (5·0 to 7·9)	1·1% (−3·7 to 4·0)
Caribbean		8830 (6730 to 11 000)	9990 (8020 to 12 200)	18 800 (15 600 to 22 300)	3460 (2670 to 4410)	5100 (4300 to 6200)	8560 (7470 to 9950)	−4·4% (−5·8 to −2·9)	−2·5% (−4·1 to −0·9)	6·5% (4·1 to 8·1)	−7·5% (−8·4 to −6·6)
	Antigua and Barbuda	2·6 (2·1 to 3·4)	6·3 (5·3 to 8·2)	8·9 (7·5 to 11·6)	2·3 (2·2 to 2·3)	5·2 (5·1 to 5·4)	7·5 (7·4 to 7·6)	−6·8% (−8·4 to −5·6)	2·7% (0·4 to 5·8)	−2·6% (−2·8 to −2·5)	−0·8% (−1·1 to −0·6)
	The Bahamas	137 (115 to 186)	227 (186 to 286)	365 (321 to 463)	47·2 (46·1 to 48·5)	67·0 (65·3 to 68·8)	114 (112 to 116)	−3·2% (−4·2 to −1·9)	3·4% (2·4 to 5·0)	1·8% (1·6 to 2·0)	−3·0% (−3·3 to −2·8)
	Barbados	26·1 (18·7 to 37·2)	43·2 (30·3 to 61·5)	69·3 (49·6 to 98·4)	8·8 (8·6 to 8·9)	16·2 (15·9 to 16·6)	25·0 (24·6 to 25·4)	−1·5% (−2·6 to 1·0)	−0·6% (−4·0 to 3·2)	−1·9% (−2·1 to −1·8)	−3·6% (−3·9 to −3·4)
	Belize	113 (86·6 to 148)	138 (122 to 167)	251 (210 to 313)	28·6 (27·9 to 29·3)	52·6 (51·2 to 53·9)	81·2 (79·5 to 82·6)	0·3% (−1·0 to 1·8)	3·7% (2·5 to 5·2)	0·3% (0·1 to 0·5)	−1·1% (−1·4 to −0·9)
	Bermuda	1·4 (1·2 to 1·6)	4·4 (3·9 to 5·0)	5·8 (5·1 to 6·6)	1·6 (1·5 to 1·6)	5·3 (5·2 to 5·4)	6·8 (6·7 to 7·0)	−8·3% (−9·5 to −7·5)	1·0% (−0·8 to 3·4)	−4·6% (−4·7 to −4·4)	−2·0% (−2·3 to −1·7)
	Cuba	684 (295 to 1180)	2130 (1140 to 3350)	2810 (1540 to 4330)	58·6 (57·3 to 60·0)	303 (295 to 310)	361 (354 to 369)	11·5% (9·8 to 13·1)	5·8% (4·1 to 7·3)	4·5% (4·4 to 4·7)	7·7% (7·5 to 8·0)
	Dominica	2·0 (1·4 to 3·2)	4·7 (3·8 to 6·2)	6·6 (5·3 to 9·4)	1·2 (1·2 to 1·2)	3·9 (3·8 to 3·9)	5·0 (4·9 to 5·1)	−5·3% (−6·4 to −4·4)	1·8% (−0·1 to 4·4)	−1·4% (−1·6 to −1·2)	−1·0% (−1·3 to −0·7)
	Dominican Republic	1740 (787 to 2930)	2220 (1210 to 3600)	3960 (2410 to 6000)	853 (332 to 1510)	1710 (1140 to 2520)	2560 (1800 to 3630)	0·2% (−2·4 to 3·0)	−3·1% (−5·7 to −0·5)	21·5% (17·9 to 24·9)	−6·2% (−8·1 to −4·5)
	Grenada	1·9 (1·4 to 2·6)	4·6 (4·0 to 5·7)	6·5 (5·5 to 8·2)	1·7 (1·6 to 1·7)	4·5 (4·4 to 4·6)	6·2 (6·0 to 6·3)	−6·2% (−7·6 to −5·0)	−0·1% (−1·8 to 2·1)	−2·8% (−3·0 to −2·7)	−0·8% (−1·1 to −0·5)
	Guyana	335 (236 to 426)	265 (190 to 354)	600 (431 to 756)	79·4 (77·6 to 81·2)	116 (113 to 120)	196 (192 to 199)	5·0% (1·2 to 8·1)	−4·2% (−5·7 to −2·3)	5·9% (5·7 to 6·0)	−3·4% (−3·7 to −3·2)
	Haiti	4590 (2990 to 6280)	3320 (2170 to 4910)	7910 (5770 to 10 500)	1860 (1290 to 2550)	1990 (1570 to 2720)	3850 (3150 to 4870)	−8·5% (−10·1 to −6·5)	−6·1% (−8·3 to −4·2)	6·4% (2·5 to 9·7)	−12·6% (−14·0 to −11·2)
	Jamaica	529 (369 to 759)	751 (590 to 968)	1280 (965 to 1700)	187 (182 to 191)	253 (247 to 259)	440 (432 to 447)	−0·5% (−5·6 to 2·9)	1·2% (−1·6 to 3·7)	3·3% (3·2 to 3·5)	−2·4% (−2·6 to −2·1)
	Puerto Rico	53·7 (45·2 to 62·8)	144 (118 to 171)	198 (165 to 231)	66·8 (65·3 to 68·4)	171 (167 to 176)	238 (234 to 243)	−14·6% (−16·0 to −13·7)	2·8% (1·1 to 5·0)	−5·7% (−5·8 to −5·5)	−5·9% (−6·2 to −5·6)
	Saint Lucia	3·4 (2·7 to 4·8)	5·4 (4·5 to 7·1)	8·8 (7·3 to 11·7)	2·9 (2·8 to 3·0)	4·9 (4·8 to 5·1)	7·8 (7·7 to 8·0)	−6·8% (−8·1 to −5·7)	1·6% (−0·5 to 4·3)	−2·2% (−2·3 to −2·0)	−2·5% (−2·7 to −2·2)
	Saint Vincent and the Grenadines	7·2 (5·9 to 9·7)	13·7 (11·6 to 17·5)	20·9 (17·6 to 27·0)	6·8 (6·6 to 7·0)	13·3 (13·0 to 13·7)	20·1 (19·8 to 20·5)	−6·2% (−8·1 to −4·4)	1·2% (−0·7 to 3·8)	0·8% (0·7 to 1·0)	−2·3% (−2·5 to −2·0)
	Suriname	93·8 (74·7 to 127)	114 (91·2 to 148)	207 (169 to 271)	40·2 (39·3 to 41·2)	61·6 (60·0 to 63·1)	102 (99·9 to 104)	−0·7% (−4·6 to 3·7)	0·2% (−1·9 to 3·1)	1·9% (1·8 to 2·1)	−3·9% (−4·1 to −3·6)
	Trinidad and Tobago	187 (151 to 250)	227 (192 to 280)	414 (354 to 512)	83·8 (81·6 to 85·9)	136 (132 to 139)	219 (216 to 224)	−1·0% (−2·9 to 1·5)	−1·1% (−2·7 to 0·9)	4·3% (4·2 to 4·5)	−2·3% (−2·6 to −2·1)
	Virgin Islands	3·9 (2·8 to 5·9)	10·7 (7·2 to 17·0)	14·7 (10·1 to 22·8)	3·0 (2·9 to 3·0)	6·0 (5·8 to 6·2)	9·0 (8·8 to 9·1)	−4·1% (−8·2 to −1·5)	4·4% (1·4 to 7·4)	0·3% (0·1 to 0·4)	−0·6% (−0·9 to −0·4)
Central Latin America		10 500 (7800 to 14 100)	35 300 (27 800 to 45 900)	45 800 (35 700 to 58 900)	3330 (3140 to 3480)	9770 (9420 to 10 100)	13 100 (12 600 to 13 500)	−0·9% (−2·4 to 0·6)	2·7% (1·0 to 4·2)	4·6% (4·5 to 4·8)	−1·8% (−2·2 to −1·6)
	Colombia	2430 (1640 to 3480)	7090 (5400 to 9580)	9520 (7040 to 12 900)	661 (627 to 697)	1980 (1890 to 2070)	2640 (2530 to 2740)	2·9% (0·4 to 5·4)	5·0% (2·4 to 7·6)	8·4% (8·1 to 8·7)	−2·3% (−2·8 to −1·9)
	Costa Rica	91·4 (62·4 to 121)	295 (217 to 406)	386 (283 to 526)	38·5 (36·5 to 40·6)	115 (108 to 122)	153 (146 to 160)	0·9% (0·1 to 1·9)	−1·5% (−3·9 to 0·3)	2·2% (1·9 to 2·6)	−0·4% (−0·9 to 0·2)
	El Salvador	458 (276 to 660)	789 (487 to 1080)	1250 (770 to 1690)	247 (86·2 to 378)	481 (235 to 685)	728 (320 to 1050)	7·1% (3·0 to 10·5)	−4·9% (−7·7 to −2·4)	11·5% (9·9 to 12·9)	−1·0% (−6·9 to 1·3)
	Guatemala	830 (375 to 1910)	1480 (709 to 3230)	2310 (1100 to 5120)	228 (217 to 240)	472 (449 to 496)	700 (676 to 727)	2·7% (−2·8 to 6·8)	−1·2% (−6·9 to 3·7)	3·2% (2·9 to 3·5)	−5·4% (−5·9 to −5·0)
	Honduras	211 (127 to 334)	268 (179 to 393)	479 (320 to 683)	25·4 (17·4 to 39·1)	37·5 (29·1 to 47·8)	62·9 (47·0 to 83·2)	0·5% (−1·9 to 2·7)	−3·0% (−7·1 to 0·3)	−0·9% (−3·2 to 3·2)	−8·2% (−11·8 to −4·8)
	Mexico	3930 (2930 to 5560)	15 900 (11 700 to 21 400)	19 800 (14 600 to 26 000)	1250 (1230 to 1260)	4330 (4270 to 4380)	5580 (5520 to 5630)	−3·8% (−5·2 to −2·4)	2·8% (0·7 to 4·8)	3·7% (3·6 to 3·8)	−2·4% (−2·5 to −2·2)
	Nicaragua	718 (490 to 1030)	1240 (794 to 1750)	1960 (1320 to 2680)	195 (103 to 275)	395 (229 to 582)	590 (333 to 835)	14·3% (10·7 to 16·5)	6·1% (3·1 to 9·1)	11·3% (9·8 to 12·8)	9·5% (4·6 to 13·4)
	Panama	426 (338 to 538)	1440 (1120 to 2000)	1870 (1490 to 2510)	128 (121 to 135)	398 (377 to 420)	526 (505 to 548)	−1·0% (−2·2 to 0·8)	4·8% (3·3 to 6·5)	3·4% (3·0 to 3·8)	−1·0% (−1·6 to −0·5)
	Venezuela	1370 (979 to 1930)	6830 (5480 to 8270)	8200 (6490 to 10 100)	557 (522 to 593)	1570 (1480 to 1670)	2130 (2030 to 2230)	3·9% (3·0 to 5·2)	2·8% (0·9 to 4·5)	3·1% (2·9 to 3·3)	−0·1% (−0·7 to 0·4)
Tropical Latin America		29 000 (20 800 to 35 000)	56 000 (41 500 to 66 700)	85 000 (62 600 to 101 000)	5720 (5530 to 5880)	10 500 (10 200 to 10 700)	16 200 (15 700 to 16 500)	2·7% (1·2 to 4·0)	3·4% (1·8 to 4·2)	1·7% (1·6 to 1·9)	−0·9% (−1·2 to −0·7)
	Brazil	28 400 (20 400 to 34 300)	55 000 (40 700 to 65 400)	83 300 (61 500 to 99 100)	5430 (5330 to 5540)	9970 (9800 to 10 100)	15 400 (15 200 to 15 600)	2·6% (1·1 to 3·9)	3·5% (1·9 to 4·3)	1·6% (1·5 to 1·7)	−1·2% (−1·4 to −1·0)
	Paraguay	638 (412 to 896)	1000 (648 to 1350)	1640 (1070 to 2220)	283 (140 to 397)	478 (261 to 666)	762 (412 to 1060)	11·8% (4·3 to 16·4)	−0·1% (−2·7 to 2·4)	11·9% (9·5 to 16·0)	5·4% (1·2 to 8·7)
**North Africa and Middle East**		**8300 (4650 to 15 300)**	**9240 (5490 to 18 900)**	**17 500 (10 700 to 32 500)**	**4690 (3410 to 6700)**	**4750 (3380 to 7020)**	**9440 (7180 to 13 100)**	**4·2% (1·4 to 7·7)**	**−0·4% (−4·0 to 3·0)**	**10·5% (8·1 to 12·7)**	**0·5% (−1·6 to 2·4)**
	Afghanistan	353 (12·2 to 1650)	569 (26·6 to 2360)	922 (41·7 to 3660)	108 (1·3 to 592)	194 (3·4 to 961)	302 (5·7 to 1450)	2·0% (−8·6 to 12·2)	8·4% (−2·2 to 16·0)	4·3% (−4·5 to 14·9)	2·6% (−21·8 to 11·3)
	Algeria	396 (4·8 to 1800)	505 (6·2 to 1970)	901 (11·1 to 3490)	130 (8·6 to 988)	197 (7·2 to 939)	327 (14·9 to 1860)	8·7% (2·3 to 14·1)	−1·0% (−42·9 to 7·8)	8·9% (1·7 to 13·1)	−2·2% (−17·4 to 4·7)
	Bahrain	4·6 (3·8 to 5·9)	15·5 (13·2 to 18·4)	20·2 (17·3 to 23·9)	3·0 (2·4 to 3·7)	8·5 (7·4 to 10·0)	11·5 (9·8 to 13·6)	−4·3% (−6·1 to −2·7)	−0·6% (−1·9 to 1·0)	1·8% (0·9 to 3·1)	−6·4% (−7·5 to −5·4)
	Egypt	154 (111 to 223)	403 (261 to 552)	557 (394 to 723)	22·5 (14·3 to 39·9)	42·3 (28·7 to 62·7)	64·8 (45·6 to 92·3)	0·6% (−1·7 to 2·7)	6·8% (3·5 to 9·3)	1·3% (−0·6 to 4·6)	−7·2% (−10·2 to −3·6)
	Iran	1540 (937 to 2200)	1610 (1110 to 2270)	3150 (2130 to 4190)	322 (273 to 371)	470 (428 to 521)	792 (707 to 889)	8·7% (1·1 to 14·5)	10·3% (6·7 to 12·5)	10·2% (9·3 to 11·4)	5·3% (4·2 to 6·4)
	Iraq	124 (61·7 to 241)	104 (52·3 to 201)	229 (115 to 443)	60·6 (41·0 to 94·3)	55·1 (36·1 to 86·5)	116 (77·9 to 181)	4·9% (0·4 to 8·1)	5·0% (0·6 to 9·1)	9·3% (6·6 to 11·6)	2·7% (−1·0 to 5·9)
	Jordan	18·5 (12·3 to 26·7)	35·2 (23·9 to 47·1)	53·7 (37·1 to 69·7)	8·5 (7·1 to 9·9)	14·5 (9·1 to 20·7)	22·9 (16·9 to 29·7)	5·8% (2·8 to 8·2)	1·1% (−2·1 to 3·7)	6·9% (6·0 to 8·1)	2·6% (−1·5 to 5·4)
	Kuwait	4·8 (4·0 to 6·4)	10·6 (7·9 to 13·5)	15·4 (12·3 to 19·2)	2·9 (2·4 to 3·4)	2·5 (2·3 to 2·8)	5·4 (4·9 to 6·0)	−3·1% (−5·0 to −1·7)	1·4% (−1·3 to 4·0)	−2·2% (−3·0 to −1·3)	−5·1% (−6·6 to −3·4)
	Lebanon	92·2 (4·0 to 403)	162 (8·5 to 802)	254 (13·1 to 1090)	48·6 (0·8 to 210)	63·9 (2·1 to 368)	113 (3·3 to 578)	−2·4% (−11·3 to 5·4)	3·7% (−6·8 to 11·7)	−0·6% (−10·4 to 5·5)	−1·2% (−13·0 to 8·4)
	Libya	95·0 (4·8 to 421)	93·9 (5·1 to 479)	189 (10·9 to 845)	47·3 (3·5 to 201)	56·0 (4·2 to 261)	103 (8·8 to 436)	5·4% (−4·9 to 14·2)	5·2% (−5·5 to 13·1)	9·1% (0·9 to 16·7)	3·2% (−5·7 to 10·4)
	Morocco	557 (28·7 to 2580)	551 (28·5 to 2790)	1110 (59·0 to 4860)	246 (6·2 to 1740)	356 (8·4 to 2260)	602 (16·0 to 3660)	5·8% (−4·3 to 13·6)	−4·0% (−15·1 to 3·9)	10·7% (−0·8 to 17·5)	−4·6% (−19·4 to 2·6)
	Oman	54·4 (30·7 to 79·9)	541 (241 to 1040)	595 (273 to 1120)	11·4 (5·8 to 19·2)	123 (62·3 to 181)	135 (70·0 to 199)	9·8% (7·1 to 11·5)	4·3% (−2·1 to 8·4)	9·9% (8·6 to 11·1)	7·3% (0·1 to 10·1)
	Palestine	9·2 (5·7 to 14·5)	10·8 (7·0 to 16·3)	20·0 (12·7 to 30·8)	4·6 (3·8 to 5·5)	5·7 (4·8 to 6·7)	10·3 (8·6 to 12·1)	4·2% (2·5 to 5·5)	2·3% (−0·3 to 4·5)	9·9% (8·8 to 11·4)	0·7% (−0·5 to 1·9)
	Qatar	2·4 (1·8 to 3·1)	8·1 (6·4 to 10·5)	10·5 (8·5 to 13·3)	2·2 (1·5 to 2·9)	4·7 (4·1 to 5·7)	7·0 (5·8 to 8·1)	−8·0% (−9·6 to −6·5)	−2·4% (−3·9 to −0·7)	−2·4% (−3·8 to −1·0)	−5·0% (−6·3 to −2·9)
	Saudi Arabia	331 (187 to 706)	342 (198 to 652)	673 (397 to 1270)	245 (191 to 324)	281 (209 to 353)	526 (402 to 676)	1·0% (−3·6 to 4·2)	−0·2% (−4·0 to 5·2)	6·4% (4·0 to 8·5)	−1·4% (−4·5 to 0·8)
	Sudan	3760 (1610 to 7140)	2810 (1410 to 5420)	6570 (3440 to 12 200)	3110 (2130 to 4230)	2190 (1650 to 3010)	5300 (4150 to 6740)	3·7% (0·7 to 8·7)	−5·7% (−11·3 to −0·3)	15·6% (12·3 to 18·0)	0·3% (−1·4 to 2·0)
	Syria	11·0 (7·2 to 16·8)	30·8 (16·5 to 52·2)	41·8 (28·7 to 62·9)	6·6 (4·8 to 9·7)	4·7 (3·2 to 8·1)	11·3 (8·2 to 17·7)	2·6% (−0·1 to 4·2)	1·6% (−1·1 to 5·7)	3·7% (2·2 to 4·7)	−4·1% (−7·0 to −0·1)
	Tunisia	180 (19·3 to 750)	251 (26·5 to 905)	431 (50·0 to 1630)	80·4 (2·4 to 392)	113 (2·8 to 455)	194 (5·4 to 790)	9·8% (2·6 to 17·1)	4·3% (−4·4 to 11·1)	11·0% (2·9 to 16·5)	5·9% (−8·0 to 11·4)
	Turkey	171 (114 to 255)	303 (203 to 438)	474 (325 to 689)	70·6 (55·8 to 85·2)	130 (102 to 156)	200 (159 to 236)	12·3% (6·9 to 50·5)	1·5% (−1·1 to 3·5)	13·5% (9·0 to 43·3)	6·5% (3·3 to 9·1)
	United Arab Emirates	42·0 (2·1 to 191)	195 (8·7 to 1210)	237 (11·2 to 1290)	23·5 (1·9 to 98·8)	181 (11·0 to 967)	205 (12·5 to 1020)	11·2% (−3·9 to 22·2)	1·8% (−9·9 to 12·3)	10·2% (1·3 to 18·2)	20·3% (−5·0 to 34·3)
	Yemen	393 (13·5 to 1820)	689 (25·9 to 3110)	1080 (43·2 to 4940)	135 (1·9 to 699)	252 (4·7 to 1320)	387 (7·3 to 1940)	1·6% (−9·9 to 13·9)	5·5% (−7·6 to 13·7)	3·9% (−5·5 to 16·3)	−0·0% (−22·1 to 9·6)
**South Asia**		**49 900 (29 500 to 82 500)**	**67 700 (39 100 to 116 000)**	**118 000 (69 100 to 195 000)**	**26 200 (22 500 to 35 100)**	**34 500 (28 300 to 51 000)**	**60 700 (51 400 to 84 900)**	**6·1% (1·1 to 11·6)**	**−1·8% (−5·4 to 2·1)**	**30·2% (23·9 to 35·9)**	**−12·4% (−14·0 to −9·4)**
	Bangladesh	414 (6·6 to 1980)	542 (15·9 to 2330)	956 (21·7 to 4120)	266 (0·8 to 1520)	361 (1·8 to 1850)	627 (2·4 to 3170)	52·2% (31·4 to 60·7)	−1·8% (−11·7 to 8·9)	43·8% (19·6 to 53·6)	8·8% (−11·1 to 18·6)
	Bhutan	41·0 (0·7 to 186)	94·2 (2·6 to 420)	135 (3·3 to 597)	24·1 (0·1 to 122)	57·2 (1·2 to 254)	81·3 (1·4 to 363)	3·9% (−10·9 to 19·1)	1·6% (−8·1 to 11·1)	8·9% (−2·9 to 22·1)	4·9% (−7·7 to 16·5)
	India	43 600 (27 600 to 65 800)	55 800 (34 600 to 82 900)	99 400 (62 900 to 148 000)	23 400 (21 800 to 25 100)	28 700 (27 100 to 30 500)	52 100 (49 200 to 55 400)	5·9% (1·1 to 11·4)	−2·3% (−5·7 to 1·8)	34·0% (30·3 to 37·4)	−13·6% (−14·3 to −12·9)
	Nepal	824 (12·9 to 3920)	1420 (37·3 to 6520)	2240 (52·3 to 9930)	762 (3·4 to 4880)	1860 (12·8 to 9710)	2620 (17·3 to 13 500)	30·1% (16·4 to 44·9)	−11·1% (−21·4 to −1·3)	65·3% (52·9 to 81·6)	−2·2% (−19·4 to 6·0)
	Pakistan	4990 (81·4 to 21 300)	9930 (306 to 42 500)	14 900 (445 to 62 900)	1780 (7·5 to 9830)	3530 (23·1 to 16 500)	5310 (31·3 to 25 900)	6·3% (−7·3 to 21·3)	7·8% (−1·7 to 18·8)	4·4% (−7·9 to 18·3)	12·9% (−11·2 to 25·5)
**Southeast Asia, east Asia, and Oceania**		**39 200 (30 600 to 50 500)**	**92 200 (69 600 to 127 000)**	**131 000 (103 000 to 175 000)**	**26 600 (22 900 to 32 800)**	**62 400 (58 300 to 68 100)**	**89 000 (81 900 to 99 600)**	**1·8% (0·3 to 3·8)**	**−3·2% (−4·9 to −1·5)**	**13·5% (12·6 to 14·8)**	**−0·6% (−1·8 to 0·5)**
East Asia		7320 (3270 to 12 800)	28 000 (11 300 to 48 800)	35 300 (14 500 to 61 500)	9070 (8330 to 10 400)	27 400 (25 400 to 30 500)	36 500 (33 800 to 40 900)	9·3% (5·8 to 11·9)	−7·0% (−13·3 to −4·6)	8·6% (7·3 to 10·3)	4·7% (3·9 to 5·3)
	China	6860 (3020 to 11 400)	26 500 (10 900 to 45 900)	33 300 (13 800 to 57 300)	8640 (8080 to 9140)	26 200 (24 500 to 27 600)	34 800 (32 600 to 36 600)	9·2% (5·5 to 11·9)	−7·2% (−13·3 to −4·9)	8·5% (7·3 to 10·2)	4·7% (3·9 to 5·2)
	North Korea	321 (2·4 to 1970)	869 (6·6 to 5270)	1190 (9·4 to 7690)	263 (3·1 to 1460)	634 (9·2 to 3590)	897 (13·6 to 5260)	11·0% (−0·2 to 27·3)	−1·6% (−16·8 to 8·3)	14·5% (1·9 to 40·9)	5·7% (−2·6 to 15·8)
	Taiwan (province of China)	21·7 (10·1 to 35·8)	189 (94·4 to 340)	210 (106 to 378)	21·8 (19·8 to 23·9)	176 (161 to 193)	198 (182 to 215)	8·8% (5·3 to 11·8)	−7·8% (−13·5 to −5·2)	7·0% (6·4 to 7·5)	4·0% (3·0 to 5·1)
Oceania		1980 (247 to 7710)	1550 (214 to 6120)	3530 (461 to 13 200)	778 (391 to 2400)	729 (342 to 2050)	1510 (747 to 4310)	18·8% (9·9 to 26·3)	−4·7% (−16·2 to 2·1)	27·4% (21·1 to 33·9)	−7·9% (−11·8 to −3·6)
	American Samoa	0·2 (0·1 to 0·4)	0·5 (0·3 to 0·8)	0·7 (0·4 to 1·1)	0·1 (0·1 to 0·1)	0·2 (0·2 to 0·3)	0·3 (0·3 to 0·4)	−1·8% (−8·6 to 4·5)	3·2% (−0·4 to 7·2)	7·0% (2·1 to 15·3)	0·6% (−1·8 to 2·8)
	Federated States of Micronesia	34·5 (0·2 to 235)	39·6 (0·4 to 246)	74·1 (0·6 to 434)	12·2 (0·1 to 51·6)	18·3 (0·3 to 83·6)	30·4 (0·5 to 131)	10·2% (−3·0 to 26·1)	7·3% (−7·3 to 18·0)	9·5% (−1·5 to 27·8)	12·7% (2·5 to 25·8)
	Fiji	24·8 (18·1 to 34·7)	22·1 (16·2 to 30·7)	46·9 (35·9 to 64·6)	5·5 (3·5 to 9·6)	4·9 (3·0 to 8·3)	10·4 (7·0 to 18·1)	2·5% (−3·9 to 7·4)	4·3% (2·8 to 6·0)	8·9% (5·8 to 14·1)	−3·2% (−6·1 to 1·4)
	Guam	0·8 (0·4 to 1·4)	6·7 (3·8 to 11·3)	7·5 (4·2 to 12·6)	0·6 (0·4 to 0·8)	3·7 (2·9 to 4·7)	4·3 (3·3 to 5·5)	−3·6% (−10·8 to 2·9)	4·0% (0·4 to 7·8)	5·0% (−0·0 to 13·9)	0·6% (−1·9 to 2·5)
	Kiribati	0·3 (0·2 to 0·7)	0·4 (0·2 to 0·6)	0·7 (0·4 to 1·3)	0·2 (0·2 to 0·3)	0·3 (0·2 to 0·3)	0·5 (0·4 to 0·6)	−6·6% (−8·0 to −5·0)	0·9% (−3·5 to 6·5)	1·3% (−1·3 to 4·1)	−3·5% (−4·3 to −2·6)
	Marshall Islands	3·9 (0·0 to 24·5)	3·8 (0·0 to 26·8)	7·7 (0·1 to 50·4)	1·4 (0·0 to 7·9)	1·3 (0·0 to 8·2)	2·7 (0·0 to 15·8)	0·1% (−11·7 to 15·0)	7·9% (−5·4 to 16·2)	5·8% (−4·2 to 20·9)	4·2% (−7·5 to 16·3)
	Northern Mariana Islands	0·2 (0·1 to 0·3)	0·6 (0·3 to 0·9)	0·7 (0·4 to 1·2)	0·1 (0·1 to 0·2)	0·3 (0·2 to 0·4)	0·4 (0·3 to 0·5)	−0·0% (−6·6 to 5·8)	3·9% (0·5 to 7·5)	8·5% (4·0 to 16·3)	1·0% (−1·2 to 3·3)
	Papua New Guinea	1730 (155 to 7240)	1320 (108 to 5660)	3050 (268 to 12 000)	688 (339 to 2200)	636 (285 to 1880)	1320 (627 to 4030)	25·2% (19·0 to 34·9)	−5·9% (−19·4 to 1·3)	48·6% (40·7 to 65·2)	−9·1% (−13·0 to −4·4)
	Samoa	13·2 (0·1 to 85·8)	13·2 (0·1 to 95·2)	26·4 (0·3 to 172)	4·6 (0·1 to 25·7)	4·5 (0·1 to 26·8)	9·1 (0·1 to 55·5)	0·7% (−11·3 to 15·9)	8·1% (−5·0 to 16·3)	6·1% (−4·2 to 21·4)	4·6% (−6·7 to 17·0)
	Solomon Islands	42·8 (0·4 to 278)	37·0 (0·4 to 268)	79·8 (0·9 to 519)	15·1 (0·2 to 91·6)	13·0 (0·2 to 77·9)	28·1 (0·4 to 166)	−0·1% (−12·2 to 14·5)	7·5% (−5·3 to 15·8)	5·9% (−4·0 to 20·8)	3·7% (−7·5 to 15·9)
	Tonga	1·1 (0·6 to 2·0)	1·5 (0·8 to 2·5)	2·6 (1·4 to 4·4)	0·5 (0·4 to 0·6)	0·7 (0·6 to 0·9)	1·2 (0·9 to 1·6)	−0·2% (−7·0 to 5·8)	4·0% (0·5 to 8·1)	8·4% (3·7 to 16·3)	1·5% (−1·0 to 3·4)
	Vanuatu	20·7 (0·2 to 137)	17·7 (0·2 to 123)	38·3 (0·4 to 254)	7·0 (0·1 to 39·1)	6·4 (0·1 to 36·7)	13·4 (0·2 to 78·5)	3·3% (−9·0 to 18·4)	2·4% (−19·4 to 15·7)	6·8% (−3·5 to 22·4)	4·0% (−5·8 to 16·0)
Southeast Asia		29 800 (24 100 to 38 400)	62 700 (52 500 to 81 000)	92 500 (78 300 to 116 000)	16 800 (13 300 to 22 800)	34 300 (30 700 to 38 900)	51 000 (44 700 to 60 300)	−1·5% (−4·2 to 1·6)	−2·2% (−3·8 to 0·5)	16·4% (15·0 to 18·0)	−3·4% (−5·5 to −1·7)
	Cambodia	561 (108 to 1390)	485 (94·4 to 1230)	1050 (208 to 2520)	502 (303 to 893)	876 (546 to 1380)	1380 (884 to 1990)	2·3% (−3·8 to 9·6)	−10·4% (−23·8 to −2·7)	36·8% (32·1 to 42·2)	−15·2% (−18·6 to −11·4)
	Indonesia	6480 (5090 to 8730)	11 300 (8800 to 15 600)	17 700 (14 100 to 24 100)	2500 (2190 to 3060)	4270 (3850 to 4870)	6770 (6090 to 7680)	5·8% (3·1 to 64·4)	3·2% (1·8 to 4·8)	32·1% (30·8 to 34·4)	6·9% (6·3 to 7·6)
	Laos	223 (1·6 to 1370)	579 (6·0 to 3920)	802 (8·0 to 5270)	138 (0·3 to 1120)	388 (1·2 to 2780)	526 (1·5 to 4300)	25·4% (14·4 to 38·9)	−6·6% (−17·3 to 1·9)	56·2% (40·6 to 67·6)	1·6% (−14·3 to 10·0)
	Malaysia	1200 (904 to 1540)	3180 (2060 to 3920)	4370 (3010 to 5230)	615 (328 to 844)	964 (744 to 1270)	1580 (1120 to 2070)	−0·7% (−3·5 to 2·3)	0·2% (−2·1 to 1·4)	19·4% (16·7 to 22·2)	−3·2% (−6·7 to −1·1)
	Maldives	0·9 (0·7 to 1·3)	0·3 (0·2 to 0·4)	1·2 (0·9 to 1·7)	0·6 (0·6 to 0·7)	0·1 (0·1 to 0·1)	0·8 (0·7 to 0·8)	4·2% (1·8 to 6·9)	−2·3% (−4·9 to 0·3)	6·5% (4·8 to 8·4)	0·5% (−2·2 to 3·3)
	Mauritius	104 (71·7 to 140)	194 (145 to 269)	298 (233 to 382)	16·4 (15·3 to 17·5)	79·4 (73·9 to 85·3)	95·8 (90·2 to 102)	20·9% (18·9 to 21·8)	3·1% (0·3 to 5·8)	6·5% (6·0 to 7·0)	5·3% (4·4 to 6·2)
	Myanmar	4970 (2730 to 7240)	4740 (2970 to 7960)	9710 (7320 to 13 300)	2860 (1640 to 4270)	2490 (2010 to 3690)	5350 (4330 to 6700)	4·6% (3·0 to 6·0)	−7·3% (−8·4 to −6·2)	34·7% (31·7 to 37·7)	−12·6% (−14·4 to −10·4)
	Philippines	3170 (1880 to 5090)	11 100 (6600 to 18 300)	14 300 (8480 to 23 200)	1390 (1330 to 1460)	2790 (2650 to 2930)	4180 (4030 to 4340)	0·0% (−8·4 to 9·3)	5·1% (1·6 to 8·6)	6·5% (6·3 to 6·7)	−3·1% (−3·5 to −2·7)
	Sri Lanka	101 (63·8 to 153)	190 (103 to 289)	291 (171 to 432)	25·2 (17·0 to 35·6)	30·6 (19·9 to 46·5)	55·8 (37·4 to 81·6)	−0·6% (−9·6 to 10·8)	7·8% (2·3 to 11·9)	4·3% (1·8 to 8·8)	−4·1% (−8·4 to −0·0)
	Seychelles	2·4 (1·9 to 3·3)	3·4 (2·6 to 4·8)	5·8 (4·5 to 8·1)	2·4 (2·2 to 2·7)	3·4 (3·1 to 3·9)	5·8 (5·3 to 6·5)	−5·1% (−6·3 to −3·7)	−1·4% (−3·3 to 0·3)	12·5% (9·1 to 15·3)	−0·3% (−1·0 to 0·4)
	Thailand	9550 (6490 to 15 600)	15 000 (12 300 to 20 900)	24 500 (19 300 to 34 300)	7660 (4790 to 13 900)	11 700 (9750 to 14 500)	19 400 (14 900 to 27 600)	−5·3% (−10·1 to −1·4)	−3·6% (−6·0 to 2·2)	14·6% (12·1 to 18·1)	−1·5% (−6·4 to 2·3)
	Timor-Leste	97·3 (0·9 to 580)	153 (1·8 to 1000)	250 (2·8 to 1540)	90·9 (1·0 to 500)	132 (2·5 to 756)	223 (3·8 to 1280)	−8·1% (−19·3 to 5·1)	−4·4% (−14·7 to 3·7)	15·1% (7·5 to 26·8)	−1·9% (−11·0 to 7·6)
	Vietnam	3360 (2330 to 6080)	15 700 (12 700 to 21 000)	19 100 (15 200 to 25 900)	933 (458 to 2630)	10 500 (8570 to 13 100)	11 400 (9060 to 15 200)	5·8% (3·9 to 8·1)	−2·3% (−3·8 to −0·5)	16·7% (14·6 to 18·5)	−1·3% (−3·0 to 1·1)
**Sub-Saharan Africa**		**723 000 (558 000 to 927 000)**	**487 000 (371 000 to 621 000)**	**1 210 000 (929 000 to 1 540 000)**	**362 000 (333 000 to 392 000)**	**349 000 (327 000 to 375 000)**	**712 000 (669 000 to 759 000)**	**−2·8% (−3·8 to −1·9)**	**−5·9% (−8·2 to −3·6)**	**7·8% (7·0 to 8·7)**	**−11·1% (−11·5 to −10·6)**
Central sub-Saharan Africa		32 100 (17 800 to 52 300)	17 200 (10 100 to 27 500)	49 300 (27 900 to 80 100)	27 700 (24 200 to 31 800)	16 600 (14 600 to 18 700)	44 300 (39 000 to 50 400)	−2·9% (−4·6 to −1·3)	−7·1% (−12·3 to −2·9)	3·4% (2·1 to 4·7)	−9·1% (−10·2 to −7·8)
	Angola	12 600 (4910 to 22 200)	6380 (2680 to 11 300)	19 000 (7540 to 32 800)	9750 (7300 to 12 700)	4840 (3670 to 6290)	14 600 (11 100 to 18 900)	12·4% (6·2 to 20·4)	−6·3% (−16·5 to 0·6)	21·7% (17·2 to 24·7)	0·3% (−1·0 to 1·7)
	Central African Republic	3470 (705 to 7620)	1520 (325 to 3400)	4990 (1070 to 11 000)	3050 (2480 to 3810)	2270 (1880 to 2770)	5320 (4490 to 6520)	−7·8% (−11·0 to −5·5)	−3·1% (−18·5 to 4·5)	5·4% (3·4 to 7·9)	−10·4% (−11·9 to −8·9)
	Congo (Brazzaville)	4390 (3070 to 5940)	2570 (1820 to 3450)	6970 (4980 to 9250)	3310 (2570 to 4140)	2260 (1850 to 2780)	5570 (4460 to 6850)	−4·5% (−6·6 to −1·7)	−2·2% (−4·3 to −0·4)	0·9% (−1·1 to 2·9)	−4·4% (−5·7 to −3·4)
	Democratic Republic of the Congo	9590 (1920 to 21 200)	5630 (1590 to 12 000)	15 200 (3500 to 33 400)	10 100 (7970 to 12 900)	6300 (5080 to 7870)	16 400 (13 200 to 20 600)	−5·3% (−7·5 to −2·8)	−10·3% (−26·0 to −2·9)	2·0% (0·6 to 3·6)	−14·0% (−15·7 to −11·6)
	Equatorial Guinea	1510 (381 to 3510)	853 (248 to 2010)	2360 (618 to 5390)	1090 (808 to 1500)	665 (481 to 921)	1750 (1320 to 2380)	8·2% (2·5 to 15·5)	−8·3% (−22·7 to −0·3)	17·5% (11·9 to 21·1)	−0·8% (−3·5 to 1·9)
	Gabon	497 (277 to 872)	228 (128 to 411)	725 (404 to 1300)	410 (282 to 641)	234 (162 to 355)	644 (465 to 963)	0·3% (−4·0 to 3·7)	−12·8% (−16·9 to −7·1)	11·4% (9·4 to 13·5)	−17·2% (−20·4 to −14·0)
Eastern sub-Saharan Africa		259 000 (187 000 to 361 000)	175 000 (128 000 to 243 000)	434 000 (314 000 to 603 000)	121 000 (111 000 to 134 000)	122 000 (113 000 to 132 000)	242 000 (226 000 to 263 000)	−5·8% (−6·9 to −4·9)	−6·2% (−9·3 to −2·5)	5·1% (4·1 to 6·3)	−13·9% (−14·4 to −13·2)
	Burundi	1880 (410 to 4450)	1160 (271 to 2610)	3040 (683 to 7270)	1160 (859 to 1600)	1460 (1210 to 1800)	2620 (2150 to 3300)	−17·8% (−24·1 to −10·7)	2·8% (−11·6 to 11·2)	10·5% (5·8 to 14·1)	−19·9% (−22·1 to −16·8)
	Comoros	3·3 (0·1 to 17·7)	10·2 (0·2 to 58·5)	13·4 (0·3 to 72·3)	0·3 (0·0 to 3·1)	1·3 (0·0 to 10·0)	1·6 (0·0 to 11·9)	10·4% (−4·6 to 35·2)	12·8% (0·7 to 23·5)	15·2% (2·6 to 41·0)	2·7% (−17·5 to 12·5)
	Djibouti	474 (132 to 1030)	345 (99·6 to 744)	820 (237 to 1720)	415 (298 to 588)	308 (222 to 432)	724 (535 to 987)	14·9% (3·8 to 26·9)	−1·5% (−12·7 to 5·0)	44·0% (32·6 to 59·1)	−6·5% (−8·7 to −4·3)
	Eritrea	1070 (267 to 2190)	695 (202 to 1380)	1770 (479 to 3570)	944 (724 to 1220)	577 (448 to 748)	1520 (1190 to 1950)	−8·5% (−14·8 to −2·3)	1·6% (−10·6 to 9·4)	15·2% (11·4 to 18·0)	−11·7% (−13·5 to −9·8)
	Ethiopia	9080 (4950 to 14 600)	5400 (3190 to 8540)	14 500 (8280 to 23 000)	9180 (7760 to 10 800)	8000 (6850 to 9360)	17 200 (14 600 to 20 100)	−18·9% (−21·5 to −16·3)	−1·3% (−7·6 to 4·1)	7·4% (4·6 to 9·6)	−19·7% (−20·9 to −18·4)
	Kenya	55 900 (38 000 to 81 100)	36 900 (24 300 to 57 800)	92 800 (64 000 to 136 000)	24 700 (22 400 to 27 800)	23 800 (21 800 to 26 500)	48 500 (44 600 to 54 000)	−6·1% (−7·5 to −4·8)	−3·5% (−6·3 to −0·5)	10·1% (9·0 to 11·1)	−11·0% (−11·7 to −10·1)
	Madagascar	5230 (585 to 24 800)	3570 (438 to 17 500)	8800 (1020 to 42 100)	1450 (1090 to 2180)	1080 (822 to 1680)	2530 (1920 to 3820)	18·4% (8·0 to 31·5)	9·8% (−10·5 to 24·4)	46·0% (36·8 to 64·4)	−3·6% (−6·5 to −0·5)
	Malawi	16 600 (7920 to 26 500)	12 000 (6410 to 18 600)	28 600 (14 300 to 45 200)	10 700 (8750 to 13 300)	11 200 (9620 to 13 200)	21 900 (18 900 to 25 900)	−7·0% (−8·5 to −5·5)	−10·8% (−18·9 to −5·3)	8·5% (6·0 to 11·4)	−17·2% (−18·5 to −14·9)
	Mozambique	79 100 (51 900 to 117 000)	54 000 (35 400 to 76 500)	133 000 (88 800 to 192 000)	31 700 (24 300 to 42 000)	30 400 (25 400 to 38 200)	62 100 (50 600 to 78 100)	5·4% (3·5 to 7·5)	−5·2% (−9·5 to −1·7)	18·4% (16·0 to 20·6)	−8·0% (−9·2 to −6·7)
	Rwanda	3930 (2540 to 5920)	2390 (1490 to 3730)	6320 (4030 to 9450)	1660 (1320 to 2120)	1300 (1070 to 1690)	2960 (2460 to 3710)	−6·1% (−9·3 to −2·1)	−7·2% (−11·7 to −2·5)	10·2% (6·4 to 12·9)	−17·0% (−18·1 to −15·3)
	Somalia	1320 (55·4 to 5050)	1060 (50·4 to 4390)	2380 (106 to 9370)	1280 (465 to 4060)	1110 (402 to 3280)	2390 (867 to 7270)	−1·4% (−10·6 to 7·8)	−5·8% (−23·7 to 1·7)	15·6% (8·7 to 21·7)	−7·0% (−9·8 to −4·6)
	South Sudan	2590 (105 to 9770)	1610 (60·5 to 5990)	4200 (162 to 15 700)	2070 (766 to 6590)	1640 (536 to 5250)	3710 (1320 to 11 600)	0·2% (−5·4 to 9·5)	−5·7% (−15·6 to −2·9)	13·0% (8·9 to 18·2)	−6·6% (−10·2 to −3·5)
	Tanzania	31 000 (1470 to 97 700)	19 400 (1350 to 61 800)	50 500 (2820 to 159 000)	12 600 (9670 to 16 800)	15 700 (12 200 to 20 000)	28 300 (22 700 to 35 200)	−8·3% (−10·8 to −6·0)	−6·6% (−41·9 to 7·7)	4·8% (3·1 to 6·6)	−17·7% (−19·5 to −15·3)
	Uganda	24 100 (3410 to 84 500)	16 200 (3020 to 52 600)	40 300 (6330 to 133 000)	12 300 (10 000 to 14 900)	13 600 (11 600 to 16 500)	25 900 (22 300 to 30 500)	−3·8% (−7·7 to 0·2)	−11·4% (−35·9 to 2·5)	−3·4% (−5·0 to −1·2)	−15·7% (−16·9 to −13·8)
	Zambia	26 600 (17 800 to 38 700)	20 200 (13 400 to 29 200)	46 800 (31 600 to 67 500)	10 300 (8110 to 13 000)	11 500 (9670 to 14 200)	21 800 (18 500 to 26 000)	−5·4% (−6·7 to −4·2)	−8·3% (−12·2 to −4·2)	5·8% (3·4 to 8·5)	−15·5% (−16·7 to −13·7)
Southern sub-Saharan Africa		213 000 (157 000 to 281 000)	153 000 (110 000 to 201 000)	366 000 (269 000 to 470 000)	81 100 (70 600 to 93 700)	87 300 (77 700 to 98 900)	168 000 (152 000 to 189 000)	2·4% (0·6 to 3·9)	−5·6% (−8·5 to −3·0)	15·0% (12·3 to 17·5)	−12·1% (−13·1 to −11·1)
	Botswana	5230 (3610 to 7200)	3860 (2580 to 5530)	9100 (6370 to 12 400)	1650 (1230 to 2460)	2450 (1620 to 3760)	4100 (3180 to 5550)	−3·1% (−4·5 to −1·8)	−5·3% (−9·1 to −1·7)	9·2% (6·7 to 12·2)	−14·7% (−17·2 to −12·1)
	eSwatini	1360 (324 to 2570)	1000 (292 to 1850)	2360 (620 to 4360)	1240 (1030 to 1460)	1270 (1060 to 1550)	2510 (2200 to 2850)	14·2% (8·5 to 19·5)	−21·8% (−36·1 to −15·1)	35·5% (30·6 to 38·5)	−15·4% (−17·3 to −12·5)
	Lesotho	7280 (5080 to 9660)	5390 (3740 to 7310)	12 700 (9140 to 16 700)	3220 (2620 to 4060)	3110 (2510 to 4050)	6330 (5330 to 7830)	2·8% (1·1 to 4·5)	−7·1% (−10·7 to −4·4)	17·9% (15·6 to 20·5)	−10·9% (−11·9 to −9·2)
	Namibia	5450 (3180 to 8970)	3950 (2320 to 6350)	9400 (5550 to 15 100)	2090 (1390 to 3130)	1870 (1400 to 2540)	3960 (3020 to 5290)	2·4% (0·8 to 5·1)	−5·8% (−10·8 to −1·2)	13·2% (8·9 to 16·4)	−12·4% (−14·6 to −10·1)
	South Africa	160 000 (120 000 to 205 000)	116 000 (85 900 to 151 000)	276 000 (211 000 to 346 000)	64 600 (54 900 to 77 300)	70 800 (61 300 to 82 200)	135 000 (119 000 to 156 000)	12·8% (11·3 to 14·2)	−5·7% (−7·9 to −3·8)	34·0% (32·5 to 35·3)	−10·5% (−11·6 to −9·4)
	Zimbabwe	33 900 (1130 to 76 700)	22 300 (1100 to 51 200)	56 300 (2210 to 124 000)	8300 (6710 to 10 300)	7760 (6530 to 9630)	16 100 (13 900 to 18 900)	−8·5% (−11·0 to −6·3)	−3·0% (−40·1 to 7·9)	7·5% (5·0 to 10·0)	−20·8% (−22·9 to −17·4)
Western sub-Saharan Africa		218 000 (152 000 to 290 000)	142 000 (99 500 to 190 000)	360 000 (258 000 to 470 000)	133 000 (111 000 to 160 000)	124 000 (106 000 to 145 000)	257 000 (223 000 to 294 000)	−1·6% (−3·1 to −0·2)	−4·9% (−7·4 to −2·5)	10·2% (8·0 to 11·8)	−6·7% (−7·3 to −6·1)
	Benin	2090 (1140 to 3250)	1320 (743 to 2090)	3420 (1870 to 5220)	1090 (801 to 1500)	719 (550 to 951)	1810 (1380 to 2390)	4·2% (0·5 to 13·5)	−5·0% (−10·2 to −0·6)	24·8% (21·8 to 27·2)	−13·2% (−15·0 to −11·3)
	Burkina Faso	1890 (404 to 3830)	1240 (314 to 2530)	3140 (725 to 6210)	1060 (775 to 1460)	1580 (1240 to 2010)	2640 (2080 to 3380)	−15·1% (−17·7 to −12·9)	−6·8% (−25·2 to 1·7)	−1·3% (−3·2 to 1·2)	−16·9% (−18·4 to −15·0)
	Cameroon	18 800 (5450 to 30 900)	10 400 (3390 to 17 400)	29 100 (8970 to 47 100)	12 300 (9480 to 16 200)	10 500 (8550 to 13 400)	22 800 (18 600 to 28 800)	3·6% (1·8 to 5·3)	−6·7% (−18·4 to −2·1)	14·0% (12·2 to 15·6)	−7·9% (−9·5 to −6·4)
	Cape Verde	170 (114 to 239)	155 (103 to 227)	324 (229 to 459)	57·2 (30·8 to 95·5)	67·6 (47·5 to 98·3)	125 (83·5 to 187)	−3·1% (−5·0 to −1·2)	−0·1% (−1·8 to 1·5)	8·2% (6·5 to 9·8)	−6·5% (−8·9 to −4·3)
	Chad	5040 (1940 to 8830)	3520 (1330 to 5960)	8560 (3260 to 14 700)	2500 (1710 to 3620)	2430 (1830 to 3270)	4930 (3660 to 6780)	−5·4% (−8·7 to −2·1)	−4·5% (−15·0 to 0·7)	9·3% (5·4 to 12·5)	−10·6% (−12·2 to −9·0)
	Côte d'Ivoire	27 300 (4380 to 48 500)	18 000 (3000 to 31 800)	45 200 (7400 to 80 300)	8520 (5650 to 11 900)	10 300 (8000 to 13 400)	18 800 (13 700 to 25 000)	−7·6% (−11·0 to 0·6)	−0·4% (−17·4 to 7·5)	2·3% (−2·1 to 6·1)	−9·3% (−11·4 to −7·0)
	The Gambia	718 (225 to 1300)	492 (153 to 899)	1210 (375 to 2150)	424 (285 to 633)	458 (342 to 632)	883 (644 to 1220)	9·2% (6·5 to 12·3)	−8·3% (−19·6 to −3·3)	21·0% (18·4 to 23·3)	−4·3% (−6·2 to −2·5)
	Ghana	15 900 (8840 to 26 000)	7210 (3910 to 12 300)	23 100 (12 700 to 37 900)	8190 (6480 to 10 600)	5690 (4540 to 7190)	13 900 (11 200 to 17 500)	−4·4% (−6·0 to −2·8)	−3·0% (−8·5 to 1·6)	6·7% (3·5 to 9·6)	−8·2% (−9·3 to −7·2)
	Guinea	3720 (2010 to 6010)	1980 (1100 to 3180)	5700 (3150 to 9090)	1750 (1280 to 2400)	1240 (950 to 1620)	2990 (2280 to 3920)	4·3% (1·5 to 7·5)	−8·2% (−13·8 to −4·5)	13·2% (10·2 to 15·6)	−8·7% (−10·5 to −6·9)
	Guinea-Bissau	1460 (516 to 2760)	875 (314 to 1670)	2330 (847 to 4320)	771 (536 to 1140)	669 (478 to 949)	1440 (1040 to 2040)	8·3% (6·5 to 10·4)	−7·8% (−17·0 to −2·7)	14·4% (12·7 to 16·1)	−5·0% (−7·0 to −2·8)
	Liberia	1410 (592 to 2500)	950 (393 to 1740)	2360 (1030 to 4090)	892 (682 to 1180)	948 (780 to 1170)	1840 (1500 to 2310)	−7·8% (−17·1 to 4·7)	2·2% (−5·5 to 10·7)	14·5% (7·7 to 19·8)	−8·9% (−11·3 to −6·3)
	Mali	4610 (3100 to 6430)	3170 (2130 to 4390)	7780 (5300 to 10 500)	3100 (2260 to 4070)	2710 (2180 to 3390)	5810 (4580 to 7350)	−0·3% (−6·7 to 6·0)	−4·9% (−7·2 to −2·9)	13·1% (8·5 to 16·3)	−5·3% (−6·9 to −4·1)
	Mauritania	17·4 (0·4 to 101)	51·9 (1·1 to 311)	69·3 (1·8 to 377)	3·3 (0·1 to 13·5)	16·3 (0·2 to 161)	19·6 (0·4 to 179)	−5·1% (−20·1 to 13·7)	−1·8% (−11·4 to 6·9)	−5·8% (−18·2 to 8·6)	−5·0% (−20·5 to 3·9)
	Niger	1140 (606 to 1810)	808 (434 to 1340)	1950 (1080 to 3020)	910 (683 to 1170)	1040 (867 to 1300)	1950 (1650 to 2360)	−4·8% (−8·7 to −1·7)	−4·5% (−9·3 to 0·2)	16·1% (14·1 to 17·6)	−12·6% (−14·2 to −10·6)
	Nigeria	128 000 (88 000 to 172 000)	88 800 (61 800 to 121 000)	217 000 (157 000 to 284 000)	87 500 (67 200 to 111 000)	81 600 (64 000 to 102 000)	169 000 (137 000 to 206 000)	0·3% (−1·5 to 1·9)	−5·1% (−7·4 to −2·7)	15·3% (13·1 to 17·9)	−5·2% (−6·0 to −4·5)
	São Tomé and Principe	0·4 (0·1 to 0·9)	1·2 (0·2 to 2·7)	1·5 (0·4 to 3·5)	0·0 (0·0 to 0·1)	0·2 (0·1 to 0·3)	0·2 (0·1 to 0·4)	−5·2% (−16·5 to 4·7)	−0·5% (−10·6 to 7·7)	0·5% (−5·2 to 8·0)	−10·8% (−15·5 to −5·5)
	Senegal	1830 (1090 to 2770)	1040 (623 to 1560)	2870 (1740 to 4320)	983 (721 to 1330)	821 (655 to 1060)	1800 (1410 to 2350)	0·4% (−1·8 to 2·4)	−5·3% (−8·8 to −2·3)	14·2% (12·1 to 16·0)	−9·1% (−10·9 to −7·6)
	Sierra Leone	1800 (222 to 4070)	1160 (134 to 2710)	2960 (371 to 6670)	1140 (807 to 1580)	1050 (818 to 1400)	2190 (1720 to 2850)	18·5% (8·6 to 35·2)	−11·4% (−34·2 to −4·0)	28·6% (22·0 to 32·1)	−5·3% (−8·2 to −2·4)
	Togo	2430 (423 to 5400)	1260 (272 to 2630)	3680 (695 to 7790)	1920 (1460 to 2590)	1950 (1530 to 2450)	3870 (3110 to 4960)	−1·0% (−3·6 to 1·4)	−10·2% (−27·8 to −2·4)	16·4% (13·9 to 18·8)	−13·0% (−14·7 to −11·0)

Data are n (95% UI) or % (95% UI). SDI=socio-demographic index. UI=uncertainty interval. GBD=Global Burden of Diseases, Injuries, and Risk Factors study.

**Figure 2:**
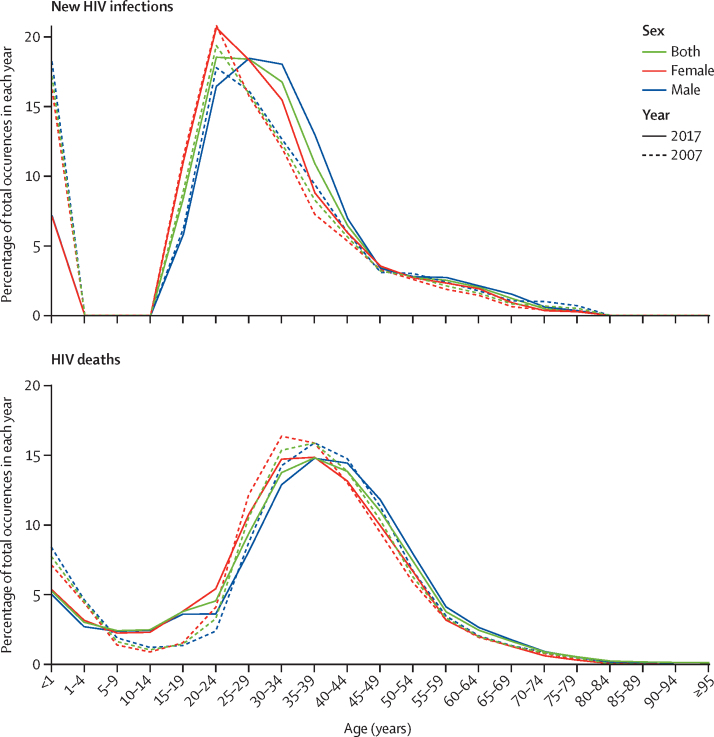
Percentage of deaths and new cases of HIV occurring in each age group, by sex, 2007 and 2017

In both 2007 and 2017, younger adults (aged 25–49 years) comprised a high percentage of all HIV deaths compared with all other age groups (figure 2). Our estimates highlight differences in HIV burden between males and females. Females aged 30–34 years had the highest percentage of HIV deaths of all female age groups, 16·4% (95% UI 15·7–16·9) in 2007 and 14·9% (13·8–15·7) in 2017, whereas males aged 35–39 years had the highest percentage of HIV deaths of all male age groups, 15·9% (14·9–16·8) in 2007 and 14·8% (13·8–15·8) in 2017. New infections among women were mostly among younger adults, with 20·8% (19·2–22·4) of new infections occurring among females aged 20–24 years in 2017, relatively unchanged from the incidence in 2007 (20·9%, 19·8–22·1). In 2017, males aged 25–29 years had the highest incidence of all male age groups, accounting for 18·6% (16·1–23·1) of new infections that year, which is a substantial change from 2007, when the highest incidence in males occurred among those younger than 1 year. Although HIV infections in children have decreased substantially with the scale-up interventions for prevention of mother-to-child transmission, in 2017, 139 555 (121 893–159 064) new infections were in children younger than 1 year, and 122 254 (112 228–132 591) HIV deaths were in children younger than 15 years. Most HIV deaths in people younger than 15 years are in children younger than 5 years, but this proportion has decreased from 82·1% (82·0–82·1) in 2007 to 63·4% (60·8–65·8) in 2017, showing the increase in lifespan for children who are HIV positive.

**Figure 3:**
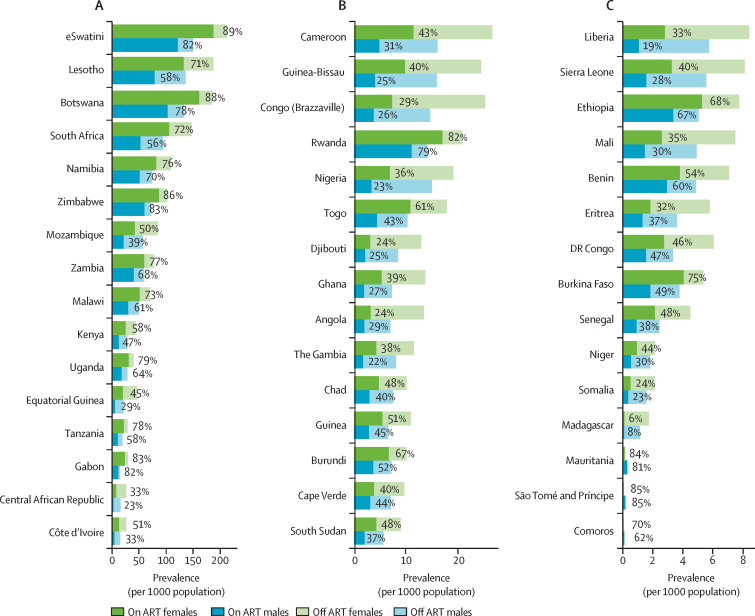
Proportion of people living with HIV who are on and off ART in sub-Saharan Africa, 2017, by country and sex Countries are subdivided by total HIV prevalences of 27 to 213 per 1000 population (A), 9 to <27 per 1000 population (B), and 0 to <9 per 1000 population (C). Proportions given in bar charts are for those on ART. ART=antiretroviral therapy.

**Figure 4:**
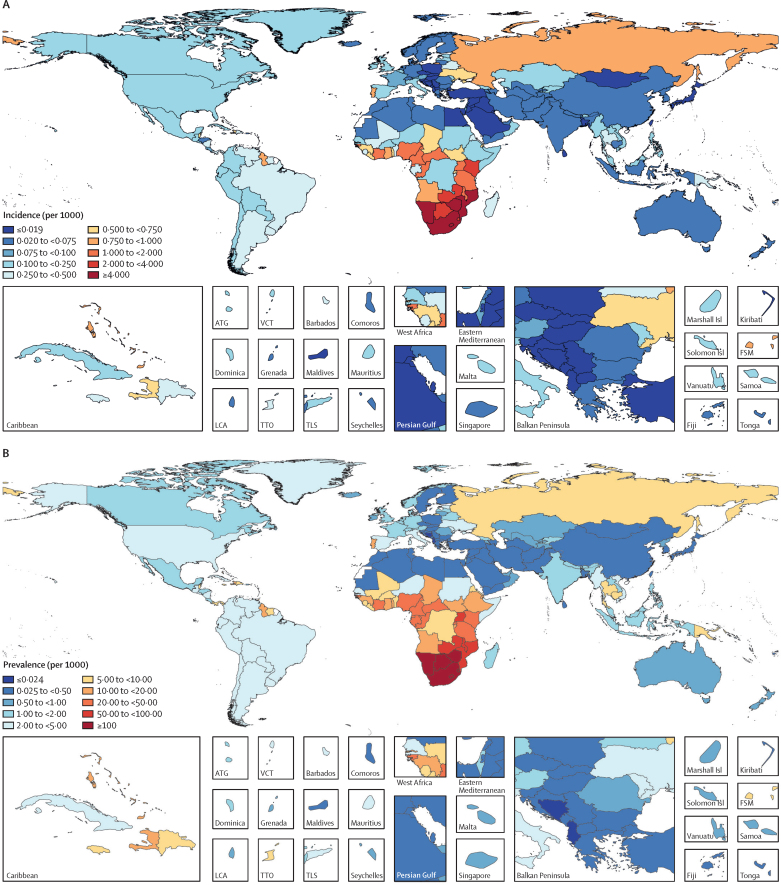

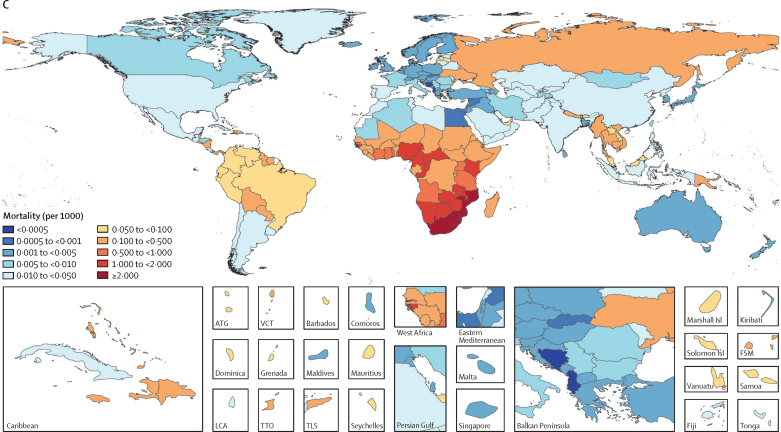
Age-standardised HIV incidence (A), prevalence (B), and mortality (C) for both sexes, 2017 ATG=Antigua and Barbuda. VCT=Saint Vincent and the Grenadines. Isl=islands. FSM=Federated States of Micronesia. LCA=Saint Lucia. TTO=Trinidad and Tobago. TLS=Timor-Leste.

**Figure 5:**
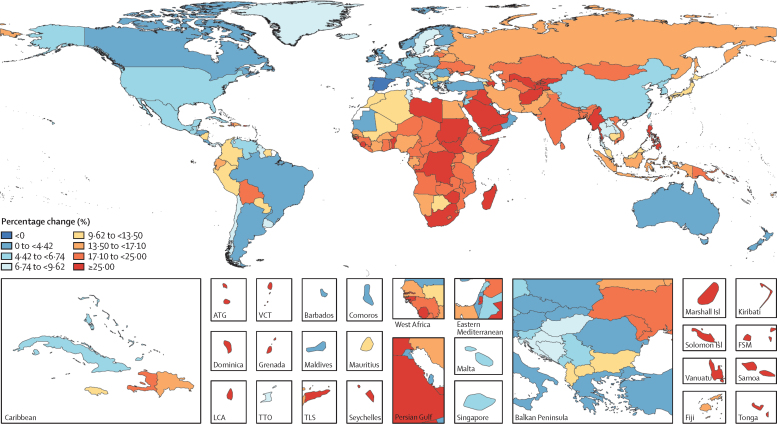
All-age annualised rate of change of ART coverage between 2007 and 2017, both sexes ART=antiretroviral therapy. ATG=Antigua and Barbuda. VCT=Saint Vincent and the Grenadines. Isl=islands. FSM=Federated States of Micronesia. LCA=Saint Lucia. TTO=Trinidad and Tobago. TLS=Timor-Leste.

Along with substantial variation in ART coverage (figure 3), sub-Saharan Africa had the highest prevalence of HIV in 2017 (figure 4). Males in three countries (Gabon, eSwatini, and Zimbabwe) and females in five countries (Gabon, Rwanda, Botswana, eSwatini, and Zimbabwe) with HIV prevalences of greater than one per 1000 population reached ART coverage of 81% or higher in 2017, reflecting early attainment of the second 90 of the UNAIDS 90-90-90 targets (figure 3). Countries across southern sub-Saharan Africa achieved higher proportions of treatment coverage than those in other sub-Saharan African regions, ranging from 66% in Lesotho to 86% in eSwatini. The estimated proportion of people living with HIV who were on treatment was higher among women than men in all but eight countries in sub-Saharan Africa (Angola, Benin, Cape Verde, Democratic Republic of the Congo, Djibouti, Eritrea, Madagascar, and São Tomé and Príncipe). Additional results detailing proportions of males and females on and off ART by GBD super-region are in appendix 2 (pp 25–31). Overall ART coverage has increased substantially over the past decade in some countries (figure 5). Between 2007 and 2017, 42 countries had annualised rates of change in ART coverage greater than 25%.

In 2017, South Africa had a higher number of new infections than all other countries, with 0·28 million (95% UI 0·21 to 0·35) new cases (table). The incidence for both sexes was highest in Lesotho in 2017, where the age-standardised rate of new cases was 6·2 (4·5 to 8·2) per 1000 population (figure 4). From 2007 to 2017, the annualised rate of change in incidence in Lesotho was −7·1% (95% UI −10·7 to −4·4), which is a drastic change from the positive annualised rate of change in incidence from 1990 to 2007 (2·8%, 1·1 to 4·5; table). Substantial progress has been made by most countries in sub-Saharan Africa, and Comoros was the only country that had an increase in incidence from 2007 to 2017, with an annualised rate of change of 12·8% (0·7 to 23·5). By contrast, many countries in eastern Europe and central Asia saw a sharp increase in the number of new infections in the past decade, with the highest annualised rate of change seen in Russia at 13·2% (10·3 to 15·5). Most countries in western Europe and North America also showed stagnant or increasing annualised rates of change in incidence (table).

Between 2007 and 2017, 122 of 195 countries had a decrease in the rate of change in age-standardised HIV mortality. Notably, the countries that achieved the most rapidly decreasing annual rates of change were Ethiopia (−19·7%, 95% UI −20·9 to −18·4), Burundi (−19·9%, −22·1 to −16·8), and Zimbabwe (−20·8%, −22·9 to −17·4; table). Lesotho had the highest age-standardised mortality in 2017, with mortality for both sexes combined being 3·4 (95% UI 3·0 to 4·0) per 1000 population (figure 4). The annualised rate of change in mortality between 2007 and 2017 in Lesotho was −10·9% (−11·9 to −9·2), and prevalence in Lesotho increased from 153·2 (142·7 to 164·7) per 1000 population in 2007 to 177·8 (168·9 to 187·8) per 1000 population in 2017 (data available from GBD datahub).

**Figure 6:**
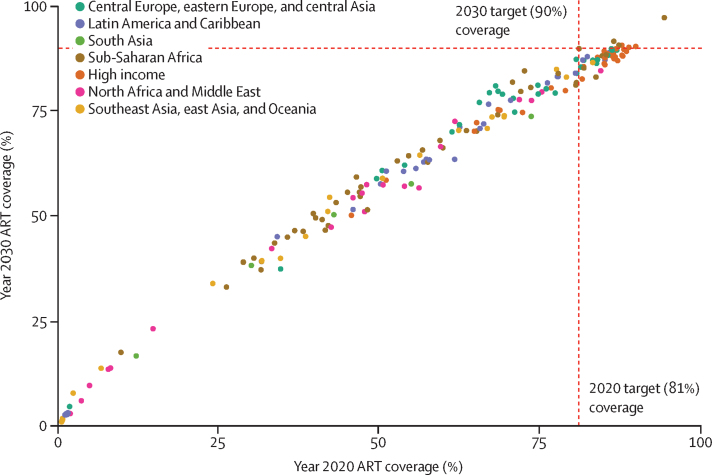
Forecast of percentage ART coverage in 2020 and 2030, by country Each datapoint is a country. ART=antiretroviral therapy.

In our forecasting, we found that progress towards meeting the ART coverage target is more optimistic than progress towards the incidence or mortality targets. Global ART coverage was forecast to be 64·8% (95% UI 61·1–67·0) in 2020 and 71·9% (68·2–75·0) in 2030 (appendix 2 pp 3–12). A substantial number of countries are predicted to meet ART coverage targets (figure 6), with 54 countries expected to meet the 2020 target of 81% ART coverage (90% started, 90% retained) and 12 expected to meet the 2030 target of 90% ART coverage (95% started, 95% retained). Of the 54 countries forecast to meet the 2020 ART coverage target, 25 are in the high-income super-region; 11 are in central Europe, eastern Europe, and central Asia; ten are in sub-Saharan Africa; six are in Latin America and the Caribbean; and two are in other regions (Mauritius and Kuwait; figure 6; appendix 2 pp 3–12). Looking ahead, 38 countries had a forecast coverage of at least 85% but less than the 90% target in 2030.

**Figure 7:**
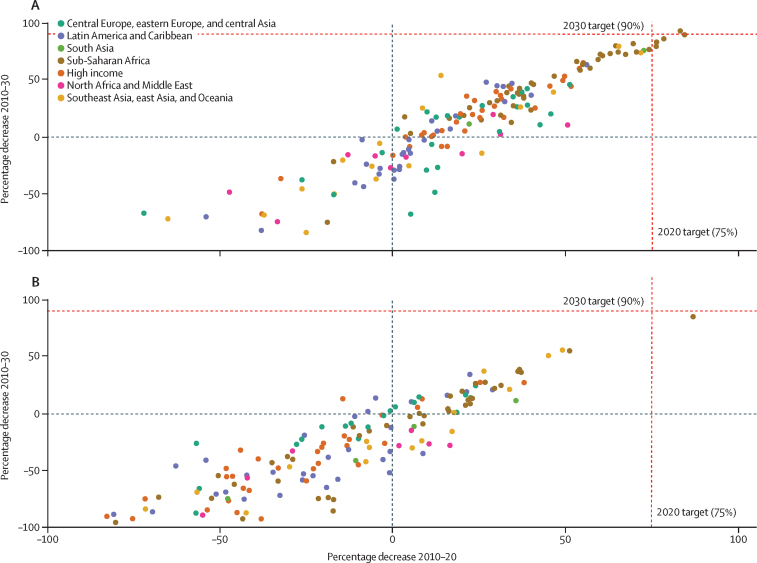
Forecast percentage decrease in HIV mortality (A) and incidence (B) from 2010 to 2020 and 2030, by country Each datapoint is a country. The plots are truncated at 100% increase, so in (A) 47 countries have been excluded and in (B) 35 countries have been excluded.

Compared with ART coverage, fewer countries are expected to achieve the UNAIDS 2020 or 2030 targets associated with mortality (figure 7; appendix 2 pp 13–18). Six countries (Burundi, Ethiopia, Gabon, eSwatini, Zambia, and Zimbabwe) were forecasted to achieve the 2020 target for mortality percentage reduction and two (Ethiopia and eSwatini) were forecasted to achieve the 2030 target. Although still short of the target, an additional eight countries (Botswana, Democratic Republic of the Congo, India, Cambodia, Myanmar, Togo, Tanzania, and South Africa) were forecasted to have a reduction in mortality of at least 65% between 2010 and 2020, and six (Burundi, Botswana, Gabon, Cambodia, South Africa, Zambia) were forecasted to have a reduction of at least 80% between 2010 and 2030. Forecasted trends in incidence show the least progress, with no countries meeting the UNAIDS 2030 target (figure 7; appendix 2 pp 19–24).

## Discussion

Globally, great progress has been made in reducing HIV-related incidence and mortality since their peaks earlier in the epidemic, consistent with the positive trend in ART coverage. Despite the scale-up of ART over time, in 2017, 40·5% (95% UI 37·8–43·7) of the 36·82 million (34·79–39·20) people estimated to be living with HIV globally were still not on treatment. In the same year, 1·94 million (1·63–2·29) people were newly infected with HIV and 0·95 million (0·91–1·01) people died from HIV-related causes. Trends in new infections and mortality are our primary indicators of progress, and over the past decade HIV incidence has been decreasing more slowly than mortality. Thus, decreases will need to be accelerated to achieve global targets. Although 54 countries are on track to meet the 2020 target of 81% ART coverage (90% started, 90% retained), only 12 countries are expected to meet the 2030 target of 90% ART coverage (95% started, 95% retained). Our forecasts show that fewer than ten countries will meet the mortality or incidence targets in 2020 and 2030.

Annualised rates of change in age-standardised HIV incidence and mortality between 2007 and 2017 varied considerably across countries. The substantial decreases in many sub-Saharan African countries underscore the enormous effort by governments and multilateral organisations to improve HIV prevention and provide effective treatment in these countries. For example, in Zimbabwe, high levels of personal exposure to AIDS deaths and prevention campaigns coupled with a relatively well educated population appear to have shifted social norms and catalysed partner reduction, which are thought to have contributed to steep decreases in HIV prevalence.^14^ eSwatini saw success after implementation of a multisectoral response with investments that prioritised ART scale-up, voluntary medical male circumcision, and prevention interventions aimed at adolescent women, mother-to-child transmission of HIV, and tuberculosis–HIV co-infection.^15^ Scale-up of a combination strategy of ART and medical male circumcision, funded by the President's Emergency Plan for AIDS Relief (PEPFAR), was found to have population-level impact in Uganda in reducing incidence.^16^ Decreases in HIV burden in Botswana have been attributed to nearly complete coverage of interventions for prevention of mother-to-child transmission and expansion of ART, which might also have reduced stigma.^17^

Conversely, between 2007 and 2017 many countries in eastern Europe and central Asia saw increasing rates of new HIV infections and persistently high mortality; Russia reached the highest annual increase in incidence at 13·2% (95% UI 10·3–15·5). The growing epidemic seen in much of eastern Europe and central Asia stems from multiple factors, including limited access to ART, inadequate access to harm-reduction services (eg, needle and syringe programmes, and opioid substitution therapy), and high levels of stigma.^18^ Opioid substitution treatment has been shown to lead to substantial reductions in risk of HIV infection among people who inject drugs; however, opioid substitution therapy remains unavailable in Russia.^19^ The particularly steep increases in new infections in Russia could also be linked to poor access to care for high-risk populations and elimination of Global Fund support.^20^

Our results showed stagnant or increasing annualised rates of change in incidence in many countries in western Europe and North America. In these countries, the HIV epidemic is largely driven by men who have sex with men (MSM).^21^ Challenges in the scale-up of HIV prevention programmes for MSM have been reported, including limited access to HIV testing and care and financial barriers.^22^ For example, the US Centers for Disease Control and Prevention estimated that MSM accounted for 66% of all HIV diagnoses in 2017 in the USA, and studies have shown high proportions of MSM are unaware of their status.^23^, ^24^ Increasing testing among high-risk populations is paramount to HIV prevention, because those who are aware of their status can decrease the risk of future transmission.^24^, ^25^ Additionally, increases in injection drug use in the USA have been linked with HIV outbreaks, and treatment efforts could be improved because fewer than 10% of those dependent on opioids are receiving substitution therapy.^26^

We found higher proportions of new infections among younger women (aged 20–24 years) than in men of the same age group in 2017. Higher risk of HIV infection among young women than in young men has been linked to several factors, including social factors such as poverty and low education, cultural factors such as transactional sex, laws that deter young women from accessing sexual and reproductive health services, and exposure to intimate partner violence.^27^, ^28^ Ambitious interventions that address multiple causes of young women's vulnerability to HIV infection in sub-Saharan African countries are underway.^29^

Few countries are on track to meet the global targets for incidence. Achieving reductions in incidence is complex because it requires increased coverage for both prevention and treatment interventions. A large body of literature has explored various intervention options.^30^ The HIV prevention cascade has been proposed as a novel framework to guide and monitor the design of interventions to maximise coverage at the population level. Krishnaratne and colleagues^30^ found that direct interventions, including overall pre-exposure prophylaxis medications and medical male circumcision, were efficacious for reducing incidence. Additionally, the consensus statement issued in 2016, and endorsed by over 760 organisations to date, states that undetectable viral load is equivalent to non-transmittable infection based on strong scientific evidence that HIV cannot be transmitted sexually by those with an undetectable viral load.^31^ This evidence suggests that increasing access to early treatment and interventions to improve adherence can support further decreases in incidence. Still, few countries are forecast to achieve the 2030 ART coverage target of 95% covered and 95% retained.

Although treatment access and prevention mechanisms exist and can be widely implemented, inadequate ART coverage and adherence could perpetuate the AIDS epidemic. Achieving exceptionally high ART coverage involves reaching groups that are difficult to target for testing and treatment. We estimate substantially lower ART coverage among men across most of sub-Saharan Africa than in other regions, which could stem from tendencies among men to delay presentation to testing and care.^32^ Additionally, in many parts of sub-Saharan Africa and other high-burden locations, large rural catchment areas further compound barriers to HIV treatment services. Developments in the area of geospatial modelling point to emerging opportunities to improve access and delivery of health interventions at the population level.^33^

Despite the substantial progress made in reducing HIV-related mortality globally, only a small number of countries are on track to meet the 2020 and 2030 mortality targets. To accelerate the decrease in HIV-related mortality, prevention, early detection, and treatment of opportunistic infections such as tuberculosis should be a priority. Integration of services for HIV and tuberculosis, a leading cause of mortality among people living with HIV, is crucial as a strategy to increase linkage to HIV care and tackle the double burden of HIV and tuberculosis.^34^

These findings should be viewed in the context of global financing for HIV/AIDS. The excitement around the MDGs and the goal to achieve universal access to treatment for HIV/AIDS by 2010 catalysed substantial resources to tackle the epidemic. Our estimates are consistent with the positive results of such global investment. However, the current and future costs of HIV/AIDS interventions and the ability of stakeholders to meet global needs will affect the epidemic going forward.

Moreover, the costs of the epidemic are likely to change. In 2015, US$27·3 billion was spent globally on HIV/AIDS care and treatment, amounting to more than half (55·8%) of the $48·9 billion spent on HIV/AIDS annually.^1^ HIV care and treatment includes services in both inpatient and outpatient settings, financed primarily through government spending and externally sourced development assistance for health. With access to ART, extended lifespans could continue to increase the total number of people living with HIV and thus further increase demand for treatment. Although the development of new therapies might reduce the per-person cost to treat those living with HIV, drug resistance that is emerging alongside increased access to ART could compound progress by increasing the cost of treatment and necessitating additional resistance testing on a wider scale.^35^ Therefore, decision makers need to anticipate the costs of providing treatment and look to prioritise prevention to reduce future costs. The GBD Health Financing Collaborator Network assessed that the annualised rate of support per prevalent case had increased between 2000 and 2010 but had subsequently decreased between 2010 and 2015.^1^ The gap that exists between available resources and what is needed to achieve global targets will require renewed mobilisation of resources, otherwise the decrease in development assistance for HIV/AIDS could hinder progress.

Although UNAIDS and GBD use similar approaches to modelling the burden of HIV, key differences exist in data and methods. At the global level, UNAIDS and GBD estimate similar mortality and prevalence; however, GBD estimates slightly higher incidence.^36^ Incidence in recent years is difficult to estimate because of the paucity of data and need to infer incidence from prevalence data. Therefore, recent estimates are highly sensitive to model specification. For group 2 countries, one primary data difference is that the GBD uses vital registration data as an input for models, using a uniform approach to cleaning data, accounting for garbage coding, and generating full time-series estimates of HIV mortality with uncertainty. While some countries might use vital registration data when producing official UNAIDS estimates, not all countries with data of sufficient quality include it as an input in their model, and heterogeneous data processing restricts comparability. Another methodological difference is that for allocation of ART to different ages, sexes, and disease severity levels, UNAIDS uses an average of the expected number of deaths and the number of people in each untreated CD4 count group to allocate ART, whereas GBD uses a model to determine the association between a country's economic status and the allocation of treatment.

Globally, more than one in eight new HIV infections occur in South Africa, making global trends highly sensitive to differences in estimates for South Africa. UNAIDS estimates for South Africa are based on a bespoke epidemic model (Thembisa), which differs in its complexity and input data from the model used for the rest of sub-Saharan Africa.^37^ The Thembisa-based model estimates a lower peak of incidence (13·81 in 1999 in UNAIDS estimates *vs* 14·58 per 1000 in 2000 in GBD estimates), a steeper decrease in incidence between 2007 and 2012 (−28% in UNAIDS estimates *vs* −12% in GBD estimates), and a slower decrease in incidence between 2012 and 2017 than GBD does (−28% in UNAIDS estimates *vs* −34% in GBD estimates).^36^ Similarly, the same model estimates a faster decrease in the number of deaths between 2007 and 2012 (−50% in UNAIDS estimates *vs* −23% in GBD estimates), and a slower decrease in deaths between 2012 and 2017 (−29% in UNAIDS estimates *vs* −45% in GBD estimates).^36^

This study has several limitations. First, our incidence and prevalence estimates in countries with vital registration data are driven by back-estimation from mortality data using assumptions of disease progression and survival. This estimation process relies on knowledge of the distribution of deaths occurring in each year for each incidence cohort. The back-calculation is inherently uncertain in more recent years when a smaller proportion of each incidence cohort has died. Second, for countries without prevalence data for which we do not run EPP, we must run a first stage of Spectrum using input incidence estimates. Our final prevalence results have shown considerable sensitivity to this initial incidence. We have attempted to mitigate this sensitivity by testing several options for input incidence and selecting the one that produces the closest fit to mortality data for each location, yet we expect that sensitivity to input incidence might still result in overestimated HIV burden in some locations, such as Portugal. Third, in group 1 countries, we have little cause-specific mortality data and rely on prevalence data and HIV mortality data derived from cohort studies to model HIV deaths. This limitation is reflected by wider UIs for results in these locations. For on-ART mortality, we pooled data from across countries in sub-Saharan Africa, and although this process results in reliable regional estimates, it means that our model does not incorporate variation in treatment quality or health system access by country. Fourth, importantly, our estimates are subject to the data available at the time of analysis. New data sources will be incorporated in future iterations of analyses as they become available. Finally, although our forecasted incidence accounts for direct effects of ART in reducing a first generation of transmission, they do not incorporate compounding secondary transmission dynamic effects.

Despite these limitations, we made several improvements in our methods compared with previous GBD analyses. In GBD 2017, we modelled ART coverage distribution in Spectrum as a function of national wealth and disease progression. Whereas previous iterations of GBD used a version of Spectrum that disproportionately allocated ART to those with lower CD4 counts, the new model reflects earlier access to treatment regardless of disease progression for people living with HIV in higher-income countries. We also improved our estimation of paediatric HIV burden by modelling natural disease progression and informing child ART initiation and mortality using cohort data. Additionally, previous iterations assumed the same sex distribution of HIV burden across countries with generalised epidemics, but we now use a model fit to the sex ratio of prevalence from representative surveys to better reflect geographical variation.

Despite the considerable progress made in reducing HIV-related mortality and increasing the coverage of ART, HIV continues to be an enormous health burden globally. Up-to-date information on the trends of the HIV epidemic from the GBD 2017 study provides an opportunity to track the success of HIV control efforts and understand where interventions are having an impact. Our results show that decreases in mortality have out-paced decreases in incidence, therefore much needs to be done to prevent new cases of HIV. Additionally, at their current rates, many countries are not on track to reach the 2020 and 2030 UNAIDS and SDG targets. To truly end the HIV epidemic, the pace of progress needs to increase. Strides in this direction can be made by continuing to expand universal access to ART and increasing investments in proven HIV prevention initiatives that can scale to have population-level effects.

## Data sharing

This study is compliant with GATHER, and data and code for the GBD 2017 HIV estimation process are available online.
