# Supplementary material for: Global, regional, and national incidence, prevalence, and mortality of HIV, 1980–2017, and forecasts to 2030, for 195 countries and territories: a systematic analysis for the Global Burden of Diseases, Injuries, and Risk Factors Study 2017
Source: Lancet HIV. 2019 Aug 19;6(12):e831–59. doi: 10.1016/S2352-3018(19)30196-1 (PMC6934077; doi:10.1016/S2352-3018(19)30196-1)
Supplement: Supplementary appendix 1 [file mmc1.pdf]

THE LANCET
HIV

Supplementary appendix

This appendix formed part of the original submission and has been peer reviewed. We post it as supplied by the authors.

Supplement to: GBD 2017 HIV collaborators. Global, regional, and national incidence, prevalence, and mortality of HIV, 1980–2017, and forecasts to 2030, for 195 countries and territories: a systematic analysis for the Global Burden of Diseases, Injuries, and Risk Factors Study 2017. *Lancet HIV* 2019; published online Aug 19. http://dx.doi.org/10.1016/S2352-3018(19)30196-1.

Appendix

Methods

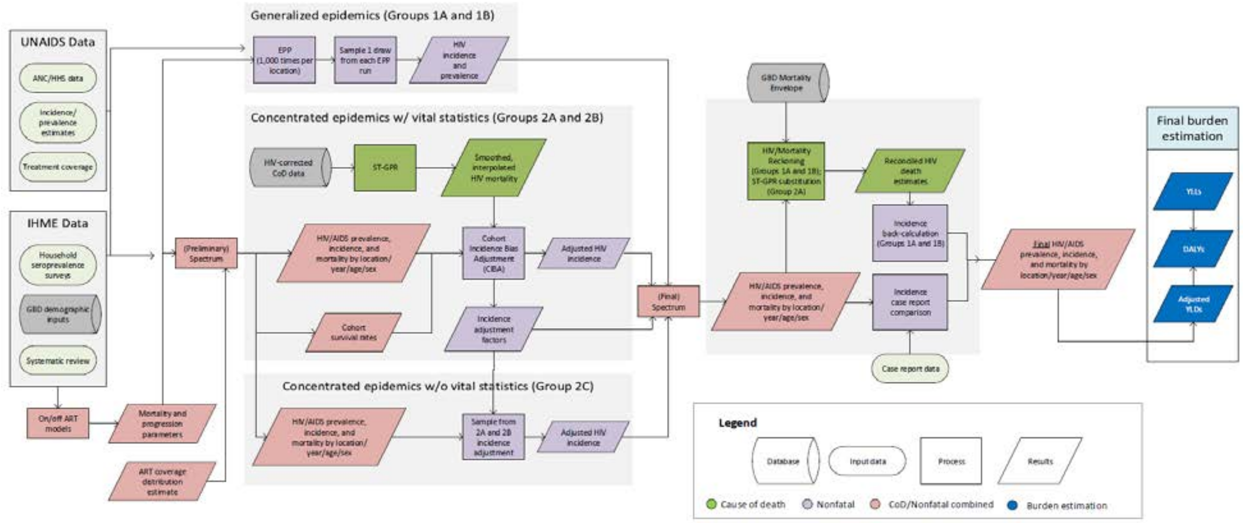

Case definition

Infection with the human immunodeficiency virus (HIV) causes influenza-like symptoms during the acute period following infection, and can lead to acquired immunodeficiency syndrome (AIDS) if untreated. HIV attacks the immune system of its host, leaving infected individuals more susceptible to opportunistic infections like tuberculosis. Although there are two different subtypes of HIV, HIV-1 and HIV-2, no distinction is made in our estimation process or presentation of results. For HIV, ICD 10 codes are B20-B24, C46-C469, D84.9; ICD 9 codes are 042-044, 112-118 (after 1980), 130 (after 1980), 136.3-136.8 (after 1980), 176.0-176.9 (after 1980), 279 (after 1980); and ICD9 BTL codes are B184-B185.

Input data

Household seroprevalence surveys

Geographically representative HIV seroprevalence survey results were used as inputs to the model for countries with generalised HIV epidemics where available.

GBD demographic inputs

Location-specific population, migration, fertility, and HIV-free survival rates from GBD 2017 were used as inputs in modeling all locations.

UNAIDS data

The files compiled by UNAIDS for their HIV/AIDS estimation process were our main source of data for producing estimates of HIV burden. Spectrum files are often built by within-country experts with the support of UNAIDS, who publishes estimates annually on behalf of countries and only shares their Spectrum files when permission is granted. These files are typically country-specific and contain both demographic data (population, fertility, migration, and HIV-free survival rates) and HIV-specific information; we substituted in our own demographic estimates for internal consistency within the GBD. The HIV-specific information from the

UNAIDS files includes what is needed to run both the Spectrum and Estimation and Projection Package (EPP) models. Spectrum requires the following inputs: AIDS mortality among people living with HIV with and without antiretroviral therapy (ART), CD4 progression among people living with HIV not on-ART, ART coverage among adults and children, Cotrimoxazole coverage among children, coverage of breastfeeding among women living with HIV, prevention of mother-to-child transmission coverage, and CD4 thresholds for treatment eligibility. EPP uses many of the same assumptions as Spectrum but fits a simpler model to HIV prevalence data from surveillance sites and representative surveys. Antenatal care, incidence, prevalence, and treatment coverage data from UNAIDS were used in modeling for all locations. We extracted all of these data from the proprietary format used by UNAIDS.

For GBD 2017, we received updated national-level files for 97 countries and updated subnational-level files for eight countries. For many of the GBD locations not covered by these files, we had UNAIDS files from an earlier year of estimation, which we used again. After combining, we were left with a set of 35 countries for which we have never received a UNAIDS file, many of them countries with small populations and/or low HIV prevalence. In those places, we generated regional averages of all needed inputs. This enabled us to run Spectrum for every GBD location.

Vital registration data

We used all available sources of vital registration and sample registration data from the GBD Causes of Death database after garbage code redistribution and HIV/AIDS mis-coding correction, except in Group 1A countries as described below.[1, 2] There are two different cause of death data sources for HIV/AIDS in China: the Disease Surveillance Point (DSP) system and the Notifiable Infectious Disease Reporting (NIDR) system. Both systems are administered by the Chinese Center for Disease Control and Prevention, but the reported number of deaths due to HIV is significantly lower in DSP. Therefore, we have used the provincial-level ratio of deaths due to HIV/AIDS from NIDR to those from DSP, choosing the larger ratio between years 2013 and 2014, and scaled the reported deaths in the DSP system, which is in turn used in the Space-Time Gaussian Process Regression (ST-GPR).

On-ART literature data

Data were identified by using search terms “HIV,” “mortality,” and “antiretroviral therapy” in PubMed searches across the literature. To be meet our criteria, studies must include only HIV-positive people who receive ART but who were ART-naïve prior to the study. In addition, studies must report either a duration-specific mortality proportion or a hazard ratio across age or sex, and must not include children.

For duration-specific survival data, studies must report uncertainty on mortality estimates or provide stratum-specific sample sizes and must include duration-specific data to allow for calculation of 0-6, 7-12, or 13-24 month conditional mortality. In addition, studies must either report separate mortality and loss-to-follow-up (LTFU) curves, be corrected for LTFU using vital registration data or double sampling, or be conducted in a high-income setting. Finally, studies must report the percent of participants who are male and the median age of participants. Hazard ratio data for ages or sexes can only be used if the hazard ratios are controlled for other variables of interest (age, sex, and CD4 category). In GBD 2013, we identified 102 papers for

extraction. For GBD 2015, we included 13 additional studies informing the duration-specific mortality estimation process and 26 studies informing the age and sex hazard ratio estimation process (some studies were used and counted in both). We also added one study to our LTFU analysis. For GBD 2016, we included 12 additional studies informing the duration-specific mortality estimation process and 11 studies informing the age and sex hazard ratio estimation process (some studies were used and counted in both). For GBD 2017, we included 17 additional studies informing the duration-specific mortality estimation process and 13 studies informing the age and sex hazard ratio estimation process (some studies were used and counted in both). We also included two new studies in our LTFU analysis.

Off-ART literature data

In GBD 2013, to characterize uncertainty in the progression and death rates, we systematically reviewed the literature on mortality without ART. We searched terms related to pre-ART or ART-naive survival since seroconversion.[3] After screening, we identified 13 cohort studies that included the cohorts used by UNAIDS from which we extracted survival at each one-year point after infection. Screening for additional, recently published studies in GBD 2015, GBD 2016 and GBD 2017 identified no new cohort studies for inclusion in this analysis.

Severity splits and disability weights

The basis of the GBD disability weight survey assessments are lay descriptions of sequelae highlighting major functional consequences and symptoms. The lay descriptions and disability weights for HIV/AIDS severity levels are shown below.

Severity level	Lay description	DW (95% CI)
Symptomatic HIV	has weight loss, fatigue, and frequent infections.	0.274 (0.184-0.377)
AIDS with antiretroviral treatment	has occasional fevers and infections. The person takes daily medication that sometimes causes diarrhea.	0.078 (0.052-0.111)
AIDS without antiretroviral treatment	has severe weight loss, weakness, fatigue, cough and fever, and frequent infections, skin rashes, and diarrhea.	0.582 (0.406-0.743)

The proportion of people living with HIV/AIDS who are being treated with antiretroviral therapy is an output of Spectrum, the compartmental model used to make consistent incidence, prevalence, and mortality estimates described below.

Modelling strategy

In GBD 2017, our general modelling strategy for estimating HIV incidence, prevalence, and mortality is very similar to the strategy used in GBD 2016. We continue to use the Spectrum program rewritten in Python for GBD 2013 to facilitate faster and more flexible execution necessary for our more intensive computational needs. We made several changes to the modeling strategy in Spectrum comparing to the Spectrum software used by UNAIDS. We also, again, ran EPP using an open-source computer program in R written by Jeffrey Eaton.[4] We ran EPP for all Group 1 countries in order to produce incidence and prevalence estimates that were consistent with the demographic and epidemiological assumptions used in GBD 2017. We explain in detail below our modifications to the assumptions of Spectrum and EPP.

We grouped countries based on quality and types of data available in order to determine modeling strategy. Group 1 includes countries with HIV prevalence data from antenatal clinics or nationally- or subnationally-representative population-based seroprevalence surveys. Group 1A included countries with a peak of at least 0.5% prevalence, and Group 1B includes countries with a peak of at least 0.25% prevalence and vital registration completeness less than 65%. Group 2 includes all other countries, which are further subdivided in Group 2A, 2B and 2C based on availability of vital registration data.

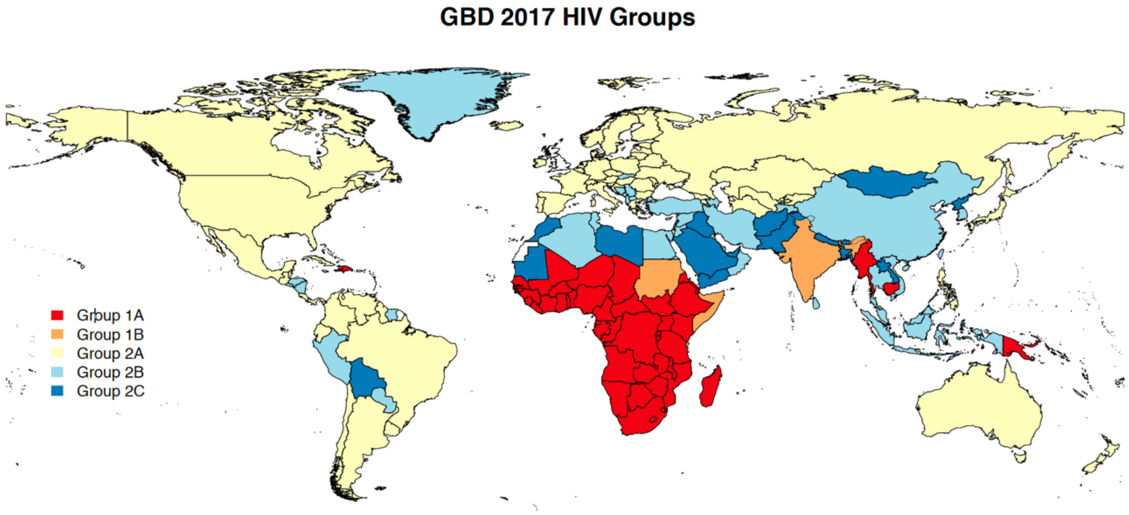

On-ART Mortality Rates

First, we corrected reported probabilities of death for loss to follow-up using an update of the approach developed by Verguet and colleagues.[5] Verguet and colleagues used tracing and follow-up studies to empirically estimate the relationship between death in LTFU and the rate of LTFU. To create estimates of age-specific hazard ratios, we synthesized hazard ratio data in five broad age groups: 15-25, 25-35, 35-45, 45-55, 55-100, and modeled the data using DisMod-MR 2.1.

To create estimates of sex-specific hazard ratios, we use the *metan* function in Stata to create estimates of relative risks separately by region, using female age groups as the reference group.

The age and sex hazard ratios were applied to the study level mortality rates, accounting for the distribution of ages and sexes in the mortality data. We then subtracted HIV-free mortality from the model life table process to calculate study level age-sex HIV-specific mortality.

We used DisMod-MR 2.1 to synthesize the age-sex split study level data into estimates of conditional probability of death over initial CD4 count.[3] We modeled the data separately by duration, age, sex and region and added a fixed effect on whether the study was conducted prior to 2002. Finally we replaced our on-ART mortality rates with those estimated off treatment if they were higher.

Changes for GBD 2017

In GBD 2017, we chose to estimate mortality for each region in its own DisMod model, whereas previous GBD iterations estimated all regions together with fixed effects. This change was driven by new results from the IeDEA cohort collaboration which provided enough data to estimate mortality trends by CD4 in each region separately.[6] We also added a year covariate to our LTFU model reflecting evidence from a large meta analysis by Zurcher and colleagues, which showed that mortality among the LTFU has declined in recent years.[7] Finally we replaced our estimated on-ART mortality rates by off-ART mortality rates, accounting for progression to lower CD4 categories, if the on-ART rates were higher. This was done to ensure individuals would not experience higher mortality when they entered treatment in spectrum.

Off-ART Mortality Rates and Disease Progression

Following UNAIDS assumptions, no-ART mortality is modeled as shown in the figure below.[3]

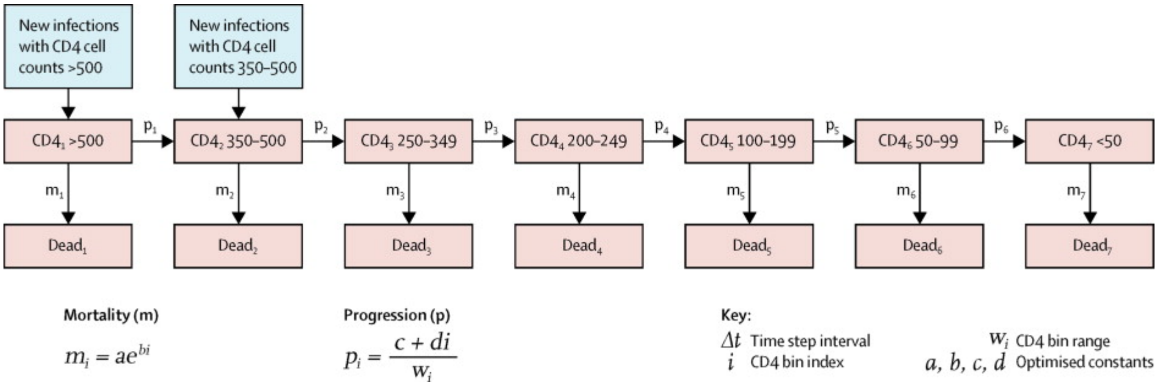

The death and progression rates between CD4 categories vary by age according to four age groups: 15–24 years, 25–34 years, 35–44 years, and 45 years or older. We modeled the logit of the conditional probability of death between years in these studies using the following formula:

$$logit\,(m_{ijk}) = \beta_0 + \sum_{i=1}^{4} \beta_{1i}\, a_i + \sum_{j=1}^{12} \beta_{2j} t_j + u_k + \varepsilon_{ijk}$$

In the formula, m is conditional probability of death from year t_j to t_{j+1}, a_i is an indicator variable for age group at seroconversion (15–24 years, 25–34 years, 35–44 years, and 45 years or

older), t_j is an indicator variable of year since seroconversion, and u_κ is a study-level random effect.

By sampling the variance-covariance matrix of the regression coefficients and the study-level random effect, we generated 1,000 survival curves for each age group that capture the systematic variation in survival across the available studies. For each of the 1,000 survival curves, we used a framework modeled after the UNAIDS optimization framework in which we find a set of progression and death rates that minimizes the sum of the squared errors for the fit to the survival curve.[8, 9]

EPP and Spectrum

UNAIDS uses two key analytical components in their epidemiological estimation. EPP is used to estimate incidence and prevalence trajectories that are consistent with prevalence surveys and antenatal clinic sero-surveillance.[4] Spectrum is a compartmental HIV progression model used to generate age-specific incidence, prevalence, and death rates from input incidence and prevalence curves and assumptions about intervention scale-up and local variation in epidemiology.

For GBD 2013, we created an exact replica of Spectrum in Python. This enabled us to run thousands of iterations of the model at once on our computing cluster and allowed for more flexible input data structures. Additionally, in order to generate estimates with more realistic ranges of uncertainty than those in UNAIDS 2012, we adjusted all input data by uniformly sampled factors between 0.9 and 1.1. These changes, along with our new estimation of with- and without-ART mortality and CD4 progression parameters, persist into GBD 2017.

In UNAIDS' implementation of Spectrum, initiation of ART is constrained by eligibility criteria and distributed across CD4 count groups according to both the expected number of deaths and the number of people in each untreated CD4 count group. Groups with a large proportion of PLHIV and high numbers of expected deaths initiated the most individuals into treatment. However, ART initiation is likely to vary based on factors outside of eligibility and expected deaths, such as health system access. We utilized data from AIDS Indicator Surveys to predict the probability of being on treatment as a function household income, stratified by CD4 count, age, and sex. This model is described in detail in the HIV/AIDS Forecasting section. The results of this prediction were translated into country-specific age-sex-year-CD4 count probabilities of coverage using a conversion factor between individual income and LDI. Predicted probabilities of coverage were input to Spectrum to inform the distribution, and not the overall level, of ART treatment by CD4 count. Spectrum converted counts of expected individuals on treatment in each CD4 count group and scaled the distribution across CD4 count groups to match the input data on the number of people on-ART coming from UNAIDS country files. In cases where the predicted number of individuals initiating treatment exceeds the total number of untreated individuals in a CD4 count group, we reallocate treatment evenly to other CD4 count groups.

Changes for GBD 2017

For GBD 2017 we improved our sex-specific modeling strategy in Spectrum by sex-splitting incidence based on a model fit to the sex ratio of prevalence observed in countries with representative surveys. We also updated the Spectrum pediatric module to reflect changes

made by UNAIDS.[10] Our pediatric module was revised to include CD4 progression and CD4-specific mortality rates taken from a model fit to survival data from IeDEA. We also updated child initiation of ART to include data on-ART distribution from IeDEA.

Countries with seroprevalence surveys and antenatal clinic data (Groups 1A and 1B)

We identified 50 countries – as well as subnational locations in India, Kenya, Ethiopia, and South Africa – with at least 0.5% adult HIV prevalence and at least one geographically representative HIV seroprevalence survey or available ANC data. In order to ensure that our estimates of incidence and prevalence in these places were consistent with our estimates of HIV progression, we used a version of EPP written in R and C++ by Jeffrey Eaton to create new fits to the available prevalence data. The version of EPP used in GBD 2017 was updated in 2017 by Jeffrey Eaton. In this new version, an ANC prevalence adjustment was included and incorporated with the 2016 lookup database for the relative risk between pregnant women and the whole adult population and an additional parameter to estimate ANC variance inflation was included as well.

For adjusting ANC data to align with the national 15 to 49 both sexes population, we extracted data on HIV prevalence among pregnant women who gave birth within the last year and attended an ANC clinic from available DHS surveys. A simple model with regional random effects was run to generate location-specific prior distributions for the ANC bias adjustment where surveys were available, and regional priors for locations without a survey. The adjustment using a time-series of relative risk between pregnant women and the adult population was removed, and the ANC bias parameter was changed to account for all of the biases observed between these two populations.

For GBD 2017, we implemented a new approach to address selection bias resulting from temporal and geographic variation in ANC reporting, which has the potential to skew unadjusted estimates, especially early in the epidemic when there are no nationally representative prevalence surveys to anchor estimates.[14] To address this issue, the specification of the likelihood of observed ANC clinic data within EPP includes random intercepts for each clinic. While this approach largely accounts for differences in level between clinics, it does not impact the estimated shape of the epidemic. In order to leverage available information from nearby geographies, we developed a model for data imputation which establishes an epidemic peak from a first-stage model fit to ANC clinic data from a location and its nearest neighbors. The model included random effects for country, clinic, and time. The year of the largest random effect was used as t_{max} the location of a single knot in an imputation model which predicted the logit of prevalence in each year for a clinic as a linear spline. We can write this method mathematically in the following way:

$$logit(\rho_i(t)) = \beta_0 + S(t) + \beta_3 X_i + \epsilon$$

$$S(t) = \beta_1 S_1(t) + \beta_2 S_2(t)$$

$$S_1(t) = \begin{cases} t_{max} - t & t \leq t_{max} \\ 0 & t > t_{max} \end{cases}$$

$$S_2(t) = \begin{cases} t - t_{max} & t \geq t_{max} \\ 0 & t < t_{max} \end{cases}$$

$\rho_i(t)$ is prevalence among ANC attending pregnant women in clinic i, with location-level intercept β_0, linear spline $S(t)$ with a knot at t_{max}, and site-specific fixed effects X_i.
One thousand draws of imputed clinic prevalence, accounting for covariance between predictors, were generated for clinic-years where at least one clinic had an observation in a given year. These draws were used for each of the one thousand EPP runs we ran for each location.

In the new version of EPP, in addition to the equilibrium prior assumption of the force of infection in projection, a random walk approach is available as an alternative method. For locations with two or more prevalence surveys and a declining trend between the mean of the most recent two surveys, the random walk approach was chosen to project the force of infection. We assumed the change of the log scaled force of infection was following a normal distribution with mean equal to the median of the change of the modeled force of infection among the years having ART implemented or prevalence data, and the SD was equal to the default setting as the mean SD of the change of the modeled force of infections among the years having prevalence data. The projection year was chosen from the most recent year between the year with the lowest model force of infection and the year of the second latest survey data.

In the new EPP code, an optimization step was added into IMIS function to speed up the parameter sampling step based on Raftery and Bao.[10] Two optimization methods have been introduced. The main algorithm is Broyden–Fletcher–Goldfarb–Shanno (BFGS) optimization. If BFGS fails, Nelder-Mead optimum is used instead. In our 2016 EPP model, by substituting in our own assumptions about HIV progression rates and on/off ART mortality, we were able to ensure that the implied relationship between incidence and mortality/prevalence in EPP is similar to that in Spectrum.

To incorporate uncertainty in our mortality and progression parameters, we run EPP with separate draws of each of these parameters. This process produced 1,000 sets of EPP output for each of the locations that make up the 48 countries in the group. Every set of EPP outputs contains 500 consistent draws of HIV incidence and prevalence in adults aged 15-49.

For every location in the group, we sampled one of the 500 incidence/prevalence draws from each of the sets of EPP results. By sampling one draw from each set, we ensured that the distribution of progression parameters dictating the relationship between incidence and prevalence was exactly the same as the distribution of the parameters generated in the previous step. At the end of this process, for every location in the set of 50 countries, we were left with 1,000 linked draws of adult incidence and prevalence and the exact mortality and progression parameters that generated those draws. We then ran these results, along with the previously described demographic and HIV-specific inputs, through Spectrum to produce location-, year-, age-, and sex-specific estimates of HIV incidence, prevalence, and mortality.

The HIV/mortality reckoning process is intended as a method of reconciling separate estimates of HIV mortality (and its resulting effect on estimates of HIV-free and all-cause mortality) in Group 1 countries by averaging estimates of HIV mortality from the model life table process

and EPP-Spectrum. Additional details on the reckoning can be found in the GBD 2017 mortality manuscript.[11]

Since Spectrum produces HIV incidence, prevalence, and deaths that are consistent with one another over time, the reckoning process results in death numbers that are no longer consistent with the incidence and prevalence produced in Spectrum. In order to recreate this consistency, we recalculated incidence for all Group 1 locations using reckoned deaths and prevalence produced by Spectrum. The updated incidence is calculated by aggregating counts of new infections, HIV deaths from Spectrum, and HIV deaths after reckoning at the year-sex level. The difference between reckoned HIV deaths and HIV deaths from Spectrum is added to Spectrum incidence, and we calculate the ratio between updated incidence and Spectrum incidence. Age-specific counts of new infections are then scaled by their corresponding sex-year ratios.

Countries with vital registration data (Group 2A and 2B)

Vital registration is one of the highest-quality sources of data on HIV burden in many countries, so generating estimates that are consistent with these data with necessary adjustment to account for any potential underreporting is critical. We identified 121 countries – as well as subnational locations from Brazil, China, Japan, Indonesia, India, Mexico, Sweden, the United Kingdom, Ukraine, Russia, New Zealand, Iran, Norway and the United States – with usable points of vital registration data, verbal autopsy (VA) data, or sample registration system (SRS) data. In India, Vietnam and Indonesia, we used SRS and VA data, respectively, as input mortality for CIBA. For India we extracted the resulting age-sex distribution of incidence, but scaled the level to match the adult incidence rate estimated from EPP for each state.

We imputed missing years of data to generate a complete time series for HIV from the estimated start year of the epidemic using ST-GPR. We analyzed mortality trends using ST-GPR starting in 1981, the year that HIV was first identified in the United States.[12] For ST-GPR, we adjusted the lambda (time weight) and GPR scale according to the completeness of vital registration data, with 4- and 5-star quality VR using parameters designed to follow the data more closely. We produced separate splines by country/age group, up to the peak year of death rate. We then ran a linear regression with fixed effects on region, age, and sex. Following this, we ran space-time residual smoothing, in which time, age, and space weights are used to inform smoothing of the residuals between data points and the linear regression estimate. From this process, we generated space-time estimates with the applied weights, along with the median absolute deviation (MAD) of the space-time estimates from the data. The MAD was calculated at various levels of the geographic hierarchy (e.g., subnational and national), and was added into the data variance term. The data variance and space-time estimates were then analyzed using Gaussian Process Regression to return a final estimate of mortality along with uncertainty.

Although Spectrum produces HIV mortality estimates that are within the realm of possibility in most countries using the incidence curves provided in the UNAIDS country files, it is a deterministic model that has not yet been integrated into an optimizable framework. Therefore, in order to “fit” it to vital registration data, we need to adjust input incidence.

To improve the fit of this process, in GBD 2015, we restructured Spectrum to track cohorts by year of HIV infection. With this version of Spectrum we can output, among many other metrics, HIV deaths by year, age, sex, and infection cohort. This enables us to adjust incidence to fit to death much more precisely and without making any rigid assumptions about the time from HIV infection to HIV death.

We have incorporated these improvements into a cohort incidence bias adjustment (CIBA) process. First, we ran Spectrum normally to produce 1,000 draws of incidence, prevalence and mortality. Then, by year, age, and sex, we took the ratio of VR deaths to Spectrum deaths to quantify the amount of bias in Spectrum. Using draw-level duration data from the new version of Spectrum, for every year-, age-, and sex-specific infection cohort, we calculated the share of all HIV deaths observed over the course of the projection period in that cohort that would occur in each year after the year of infection. For example, projecting from 1970 through 2016, we identified the cohort of men infected in 1992 at the age of 16, calculated the total number of HIV deaths in that cohort in all subsequent years through the end of 2016, and divided the annual number of deaths by that total. This showed us the distribution of deaths among that cohort over the projection period. In the most extreme case (infections in 2015), we could only produce one point of that distribution (2016), so that single value is exactly 1·0; 100% of the deaths observed in that cohort occurred in 2016.

We then used these distributions of death to weigh the ratio of VR deaths to Spectrum deaths, meaning that ratios in the years where we expect the largest share of deaths were weighed most heavily. We then multiplied the initial size of that cohort from the normal run of Spectrum by the sum of the combined ratios to get a new estimate of new cases in that year/age/sex combination.

We can write this method mathematically in the following way:

$$r_t = \frac{VR_t}{D_t}$$

$$\rho_t^{t-i} = \frac{d_t^{t-i}}{\sum_{k=t-i+1}^{n} d_k^{t-i}}$$

$$\alpha^{t-i} = \sum_{k=t-i+1}^{n} r_k * \rho_k^{t-i}$$

$$n_{\text{adjusted}}^{t-i} = \alpha^{t-i} * n^{t-i}$$

VR_t is the number of HIV/AIDS deaths in year t from ST-GPR, and D_t is the number of HIV/AIDS deaths from the first run of Spectrum. In the second equation, d_t^{t-i} is the number of HIV/AIDS deaths among members of infection cohort $t - i$ in year t, with $i \geq 1$, from the new, duration-tracking version of Spectrum, and n is final year of the projection. Therefore, ρ_t^{t-i} is the share of observed deaths in cohort $t - i$ that we expect to occur in year t. It follows that α^{t-i} is the weighted adjustment ratio described above, which we multiply by the estimated initial size of infection cohort $t - i$ as calculated in the first-stage Spectrum run to get the adjusted number of new cases, $n_{\text{adjusted}}^{t-i}$. This process is run separately for every sex, single-age, and draw.

CIBA allows ratios in each year after a given infection year to influence the final adjustment to incidence. The size of that influence is determined by the relative importance of that year in the cohort-year's distribution of deaths over time. The result is a new set of 1,000 draws of incidence and a set of 1,000 ratios of post-adjustment incidence to pre-adjustment incidence. We perform this adjustment using mean durations from the new version of Spectrum in order to try to shift the mean of the regular distribution of deaths.

Finally, to produce location-, year-, age-, and sex-specific estimates of HIV incidence, prevalence, and mortality, we ran the new estimates of incidence and all previously input data through Spectrum.

Countries without survey data or vital registration data (Group 2C)

The remaining 24 countries had neither geographically representative seroprevalence surveys nor reliable vital registration systems. To produce estimates of HIV burden in these countries, we assumed that Spectrum is similarly biased as in other Group 2 countries within the same super-region. This involved running Spectrum, adjusting incidence using 1,000 adjustment ratios randomly sampled from CIBA results from the same super-region, and rerunning Spectrum using the new draws of adjusted incidence. As above, the estimates of incidence, prevalence, and mortality were incorporated into the rest of the machinery via the reckoning process.

Source Counts

On/Off ART Mortality	**Other**
**Site-years (total)**	17045
**Number of countries with data**	148
**Number of GBD regions with data (out of 21 regions)**	21
**Number of GBD super-regions with data (out of 7 super-regions)**	7

HIV Prevalence	**Other**
**Site-years (total)**	2037
**Number of countries with data**	51
**Number of GBD regions with data (out of 21 regions)**	9
**Number of GBD super-regions with data (out of 7 super-regions)**	5

HIV Incidence case reports	**Other**
**Site-years (total)**	666
**Number of countries with data**	54
**Number of GBD regions with data (out of 21 regions)**	7
**Number of GBD super-regions with data (out of 7 super-regions)**	3

Site-Year Maps by Source Type

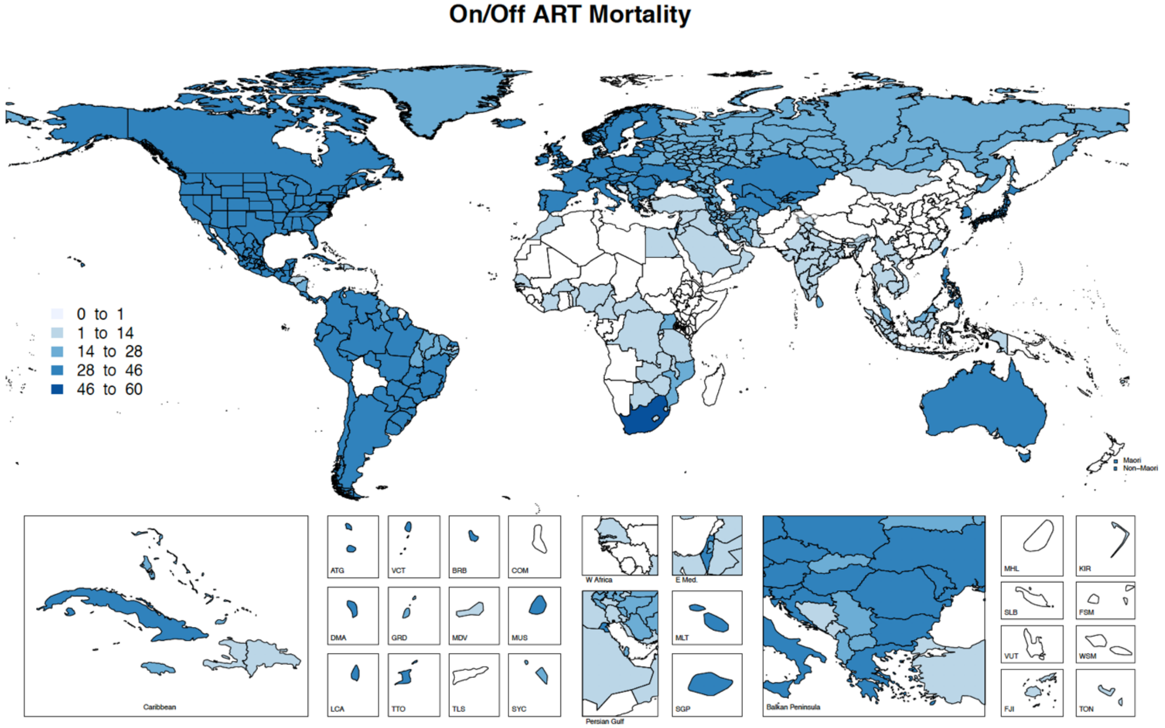

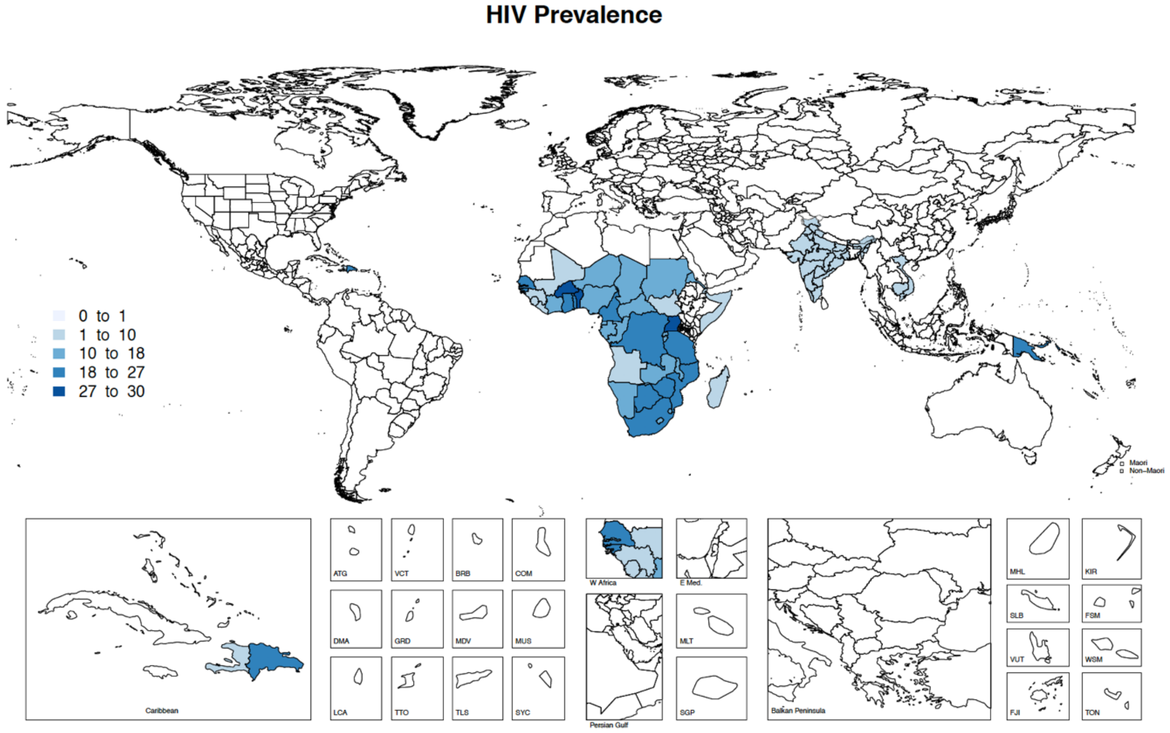

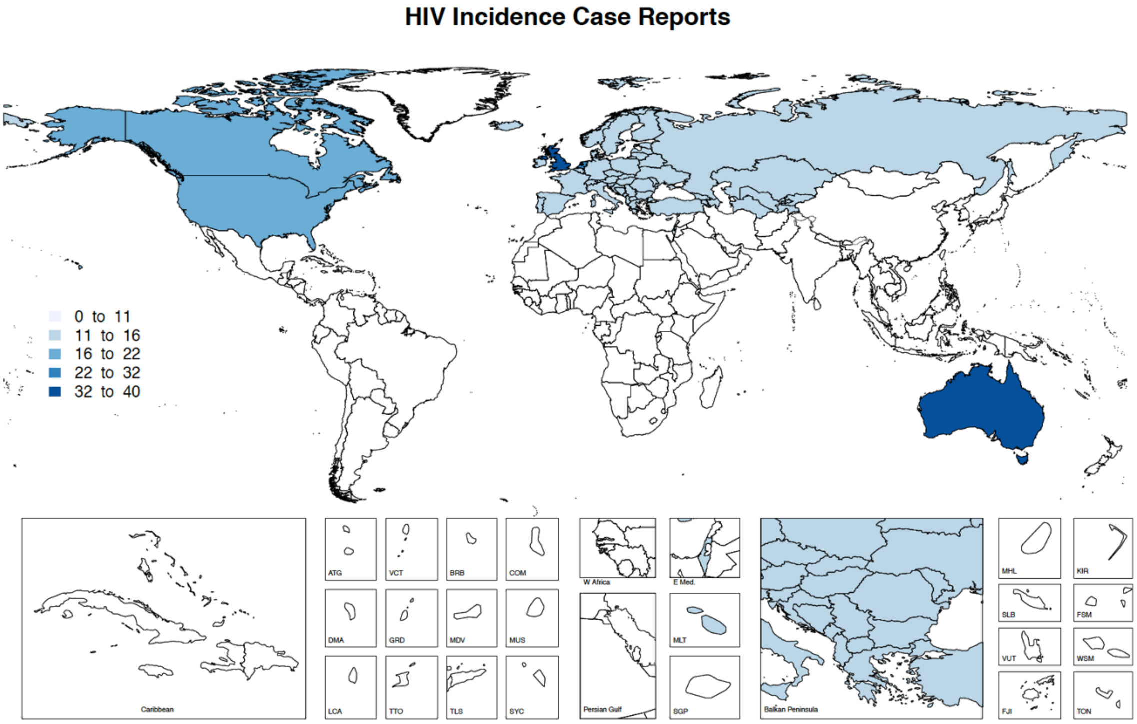

References

1. Global, regional, and national age–sex specific all-cause and cause-specific mortality for 240 causes of death, 1990–2013: a systematic analysis for the Global Burden of Disease Study 2013. *The Lancet* 2015; **385**: 117–71.

2. Birnbaum JK, Murray CJ, Lozano R. Exposing misclassified HIV/AIDS deaths in South Africa. *Bull World Health Organ* 2011; **89**: 278–85.

3. Murray CJL, Ortblad KF, Guinovart C, *et al.* Global, regional, and national incidence and mortality for HIV, tuberculosis, and malaria during 1990–2013: a systematic analysis for the Global Burden of Disease Study 2013. *The Lancet* 2014; **384**: 1005–70.

4. jeffeaton/epp. GitHub. https://github.com/jeffeaton/epp (accessed April 21, 2016).

5. Verguet S, Lim SS, Murray CJL, Gakidou E, Salomon JA. Incorporating Loss to Follow-up in Estimates of Survival Among HIV-Infected Individuals in Sub-Saharan Africa Enrolled in Antiretroviral Therapy Programs. *J Infect Dis* 2013; **207**: 72–9.

6. Anderegg N, Johnson LF, Zaniewski E, *et al*. All-cause mortality in HIV-positive adults starting combination antiretroviral therapy: correcting for loss to follow-up. *AIDS* 2017; **31 Suppl 1:** S31-40.

7. Zürcher K, Mooser A, Anderegg N, *et* al. Outcomes of HIV-positive patients lost to follow-up in African treatment programmes. *Trop Med Int Health* 2017; **22**: 375-387.

8. Ghys PD, Zaba B, Prins M. Survival and mortality of people infected with HIV in low and middle income countries: results from the extended ALPHA network. *AIDS Lond Engl* 2007; **21 Suppl 6**: S1–4.

9. Hallett TB, Zaba B, Todd J, *et al.*, ALPHA Network. Estimating incidence from prevalence in generalised HIV epidemics: methods and validation. *PLoS Med* 2008; **5**: e80.

10. Raftery AE, Bao L. Estimating and Projecting Trends in HIV/AIDS Generalized Epidemics Using Incremental Mixture Importance Sampling. *Biometrics* 2010; **66**: 1162–73.

11. Wang H, Murray CJ, Carter A, He F. Global, regional, and national under-5 mortality, adult mortality, age-specific mortality, and life expectancy, 1970-2016: a systematic analysis for the Global Burden of Disease Study 2016. *The Lancet* 2017; **390**: 1151-1210.

12. CDC. Pneumocystis Pneumonia --- Los Angeles. MMWR Wkly. 1981; published online June 5. http://www.cdc.gov/mmwr/preview/mmwrhtml/june_5.htm (accessed April 21, 2016).

13. CDC. Pneumocystis Pneumonia --- Los Angeles. MMWR Wkly. 1981; published online June 5. http://www.cdc.gov/mmwr/preview/mmwrhtml/june_5.htm (accessed April 21, 2016).

14. Ng M, Gakidou E, Murray CJL, Lim S. A comparison of missing data procedures for addressing selection bias in HIV sentinel surveillance data. *Population Health Metrics* 2013; **11:** 12.

HIV/AIDS forecasting

Coverage of antiretroviral therapy for HIV/AIDS

In recent years, we have seen a massive scale up of ART treatment among low-income nations, who through large internal investments and substantial development assistance have been able to increase ART access considerably. For that reason, if past trends in ART coverage for each country are simply scaled up in projections using a logistic curve, all countries would be projected to achieve 100% coverage by 2030. Given limitations on coverage by health system capacity, and due to the cost of treatment, we bound ART projections with a frontier by income level to reflect resource availability.

Cross-walking Cross-Sectional and Spectrum CD4 Definitions

In order to model the relationship between income and ART coverage, we must also consider CD4 count as a major stratifying variable, since individuals who are sicker (with lower CD4 counts) are more likely to have received a diagnosis and treatment. Survey data provides cross-sectional CD4 count information; however, the Spectrum modeling framework tracks individuals by categorical CD4 count at the initiation of treatment. Therefore, in order to model the relationship between CD4-specific ART coverage and income in a format that aligns with Spectrum, we cross- walked cross-sectional CD4 values to CD4 at treatment initiation.

We extracted information on the average CD4 progression over time after the initiation of ART treatment from a number of cohort studies.[1-8] We used a natural spline model to parameterize CD4 count response to treatment over time. Our outcome variable , was the difference in the average CD4 count for a cohort i at time *t* from the value at the beginning of treatment, time *t0*:

$$Yi,=CD4i,t- CD4i,t0$$

We model this change over time using the following model:

$$Y_{i,}=S_1t*CD4_{i,t0}+S_2t$$

Where $S2t$ is a natural spline on the number of months since treatment initiation, and ($S1t*CD4i$,0) is a natural spline on the number of months interacted with the starting average CD4 count of the cohort. Both spline bases use knots at 3, 12, 24, and 36 months. The model was fit, for each of the CD4 cut-points used to define compartmental categories in the Spectrum modeling framework (0-49, 50-99, 100-199, 200-249, 250-349, 350-500, and 500+). We then use the progression curves from this model to categorically backcast each individual observed in our cross-sectional survey data sourcs to one of the aforementioned categories.

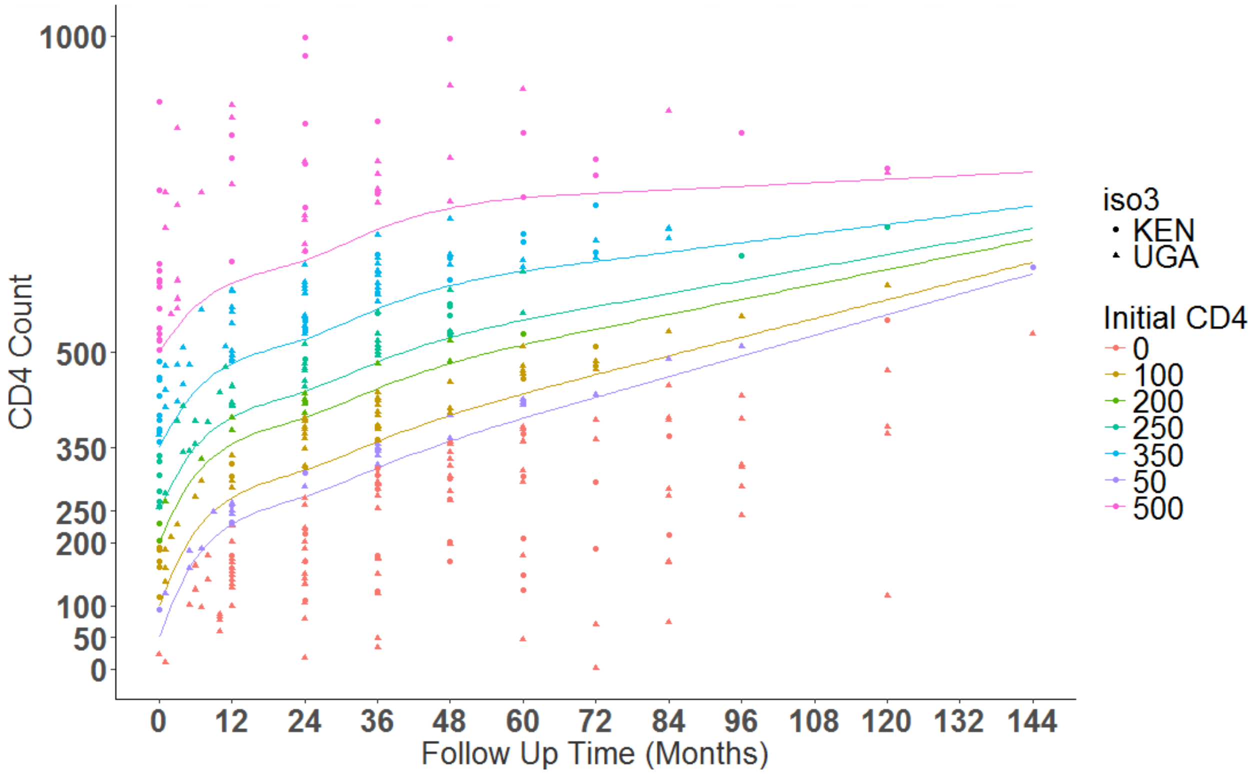

Figure 1. Categorical backcast of survey microdata using modelled progression curves.

Modeling ART Coverage Frontier as a Function of Income and CD4 Count

To obtain realistic forecasts of ART coverage it is important to place bounds on the coverage relative to resources that are expected to be available. We identified three publically available survey datasets, the 2011 Uganda and 2012 and 2007 Kenya AIDS Indicator Survey, that provide person-level information regarding the distribution of ART coverage by CD4 count. CD4 information for each participant was obtained from laboratory test values, and cross-walked to the Spectrum definition as described in the previous section. As a proxy for income, we used a household asset index based on assets present in the respondent's home, converted to international dollars.[9] A logistic curve describing the relationship between ART coverage and income is then fit, controlling for CD4 count, age and sex, using a logistic regression:

$$(Pcoverage) = \beta 0 + \beta 1 Income + \beta 2 CD4 + \beta 3 Age + \beta 4 Female$$

We used the predicted probabilities from this model to fit a stochastic frontier analysis, which estimates the maximum possible coverage for a given degree of income and CD4 count. Formally, we estimate:

$$\log\left(logit(Pcoverage) + offset\right) = \beta 0 + \beta 1 Income + \beta 2 CD4$$

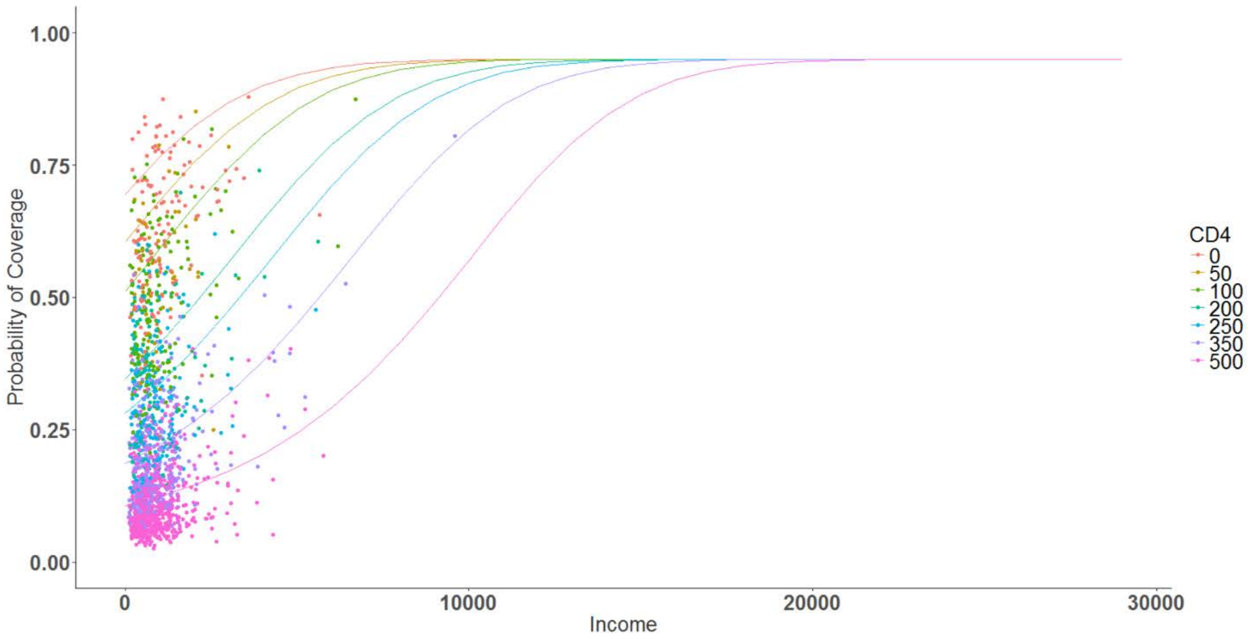

Figure 2. Predicted probabilities of coverage for each individual shown as points. Frontier of coverage as a function of income is shown with lines. Color indicates categorical CD4 count.

Forecasting ART Prices

In order to forecast ART coverage, an understanding of the cost of ART treatment over time is necessary. We created estimates and projections of the average cost of ART treatment using data from the Global Price Reporting Mechanism (GPRM).[10] From the GPRM we obtained 1,175 country-years of data representing the average cost of ART in dollars per person per year, covering 130 countries and spanning 2004-2016. We used a stochastic frontier analysis and Gaussian process regression modelling framework to complete the time series and project the estimates through 2030.

Stochastic Frontier Analysis

In order to bound the future minimum cost plausibly, we use a stochastic frontier analysis to model the minimum ART price possible over time. First we create the outcome variable by transforming year-specific cost through rescaling to an inverse zero to one scale, where 0 is the lowest observed cost and 1 is the highest across all years for a given country. This is necessary as the stochastic frontier analysis function is used to find a maximum value. Therefore, the outcome must be rescaled to find a minimum cost frontier. We then take the logit of this transformed cost variable, which creates our outcome variable:

$$Y_{c,t} = logit(\ (Cost_{c,t} - \min\ (Cost_{c}))\ /\ range(Cost_{c}) + offset)$$

We then fit a stochastic frontier analysis, with time as the independent variable, assuming a truncated normal distribution for the extent to which countries fall short of obtaining the minimum achievable ART price.

Gaussian Process Regression

We used Gaussian process regression (GPR) to complete the time series and make projections through the year 2030. GPR has been used extensively in the Global Burden of Disease estimation framework as a data synthesis tool.[11, 12] The mean function is a linear model which models the log of the difference between the cost frontier and the current cost, as a function of lag-distributed GDP per capita (LDI) and super-region secular trends:[13]

Consistent with prior implementations of GPR, a Matérn covariance function was used to smooth the residuals from the first stage mean function, and produce complete time series with uncertainty.[12]

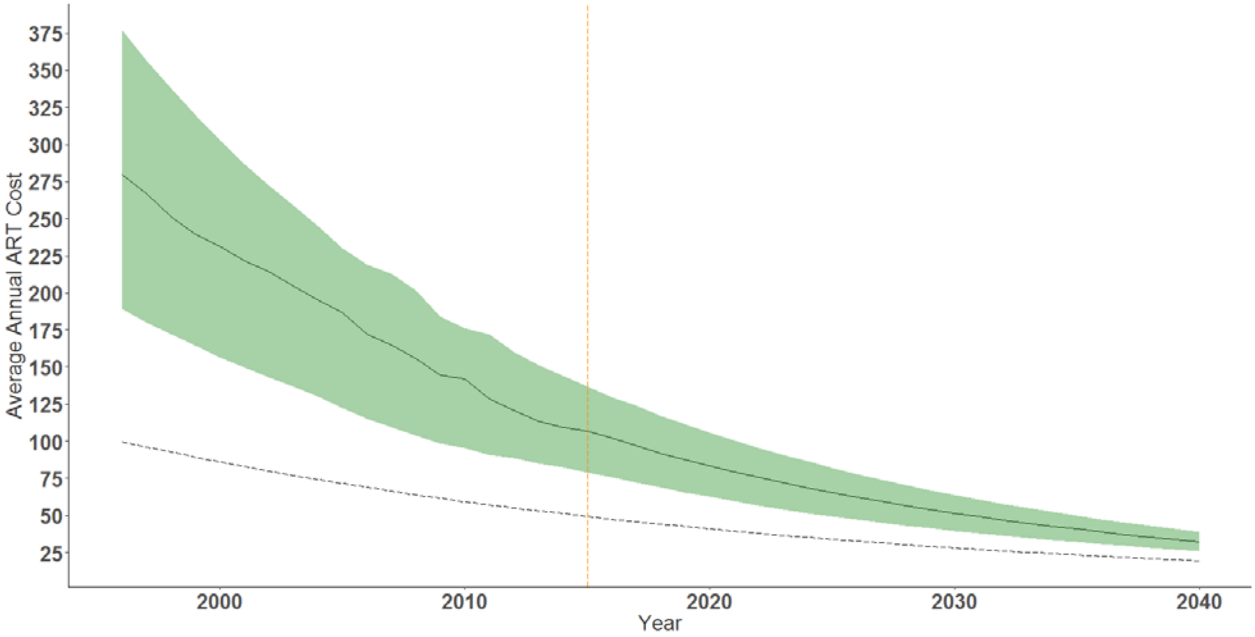

Figure 3. Median and IQR of ART price over time globally, alongside the cost frontier as a dashed line. All series are shown in USD.

Forecasting Spectrum Inputs

A number of inputs to ART forecasting, incidence hazard forecasting, and Spectrum HIV modeling systems are treated as exogenous inputs. Projection for these inputs were created using a rate of change approach, consistent with that used across the GBD forecasting platform. These inputs include:

- ART Price
- Lag Distributed GDP per capita
- Child ART coverage
- Cotrimoxazole coverage among children
- Coverage of medication used to prevent mother-to-child transmission (PMTCT) of HIV in the prenatal and postnatal periods

For each indicator, the distribution of the rate of change across countries was calculated. The time series in each indicator was projected assuming each country grows in the future at the 50th percentile of the past rate of change across countries. Inputs that represent a coverage

indicator, including PMTCT, cotrimoxazole, and ART, were forecasted in logit space, while the remaining indicators were modeled in log space.

Forecasting ART Coverage

ART coverage is projected using the ART bounds described above in the ART coverage frontiers section, as well as spending on HIV care and treatment that is forecasted independently.[13] In order to account for the changing costs of ART over time, the HIV spending covariate is rescaled to "dose equivalents" by dividing by ART cost. The relationship between country-year specific ART coverage is then modelled with a slope on dose-equivalents of HIV spending and fixed intercepts for each CD4 group.

$$DoseEquivalents = HIV\ spend/ART\ cost$$

$$(ARTc,t) = \beta 1 DoseEquivalents + (\beta 2 CD40{-}49 * I0{-}49) \ldots (\beta 8 CD4500{+} * I500{+})$$

Projected ART values were bounded using the frontiers estimated as described above, or the largest value observed in the past for the time series in question, whichever is larger. We then forecast ART coverage at the granularity it is used in Spectrum, specific to single-year age and sex groups, as well as draws used in Spectrum to propagate uncertainty:

$$(ART_{c,a,s,t,d}) = \beta 1 DoseEquivalents + (\beta 2 CD40{-}49 * I0{-}49) \ldots (\beta 8 CD4500{+} * I500{+}) + \emptyset c,a,s,t,d$$

where $\emptyset c,a,s,t,d$ is a country-year-age-sex-draw specific intercept shift term, used to ensure no disjunctions in the first year of the forecasts by removing the difference from year 2017 to year 2018 from all forecasted estimates for each time series.

HIV Incidence Hazard

Incidence hazard, the rate of new infections among the susceptible population, is a key input to the Spectrum modeling process. We forecast incidence hazard using ART projections as well as a rate of change approach similar to those described above with respect to the trend in the counterfactual incidence hazard, the expected hazard if ART coverage were zero. A time series of incidence hazard from 1970 through 2017 for each location is taken from GBD 2017 final estimates, then counterfactual incidence hazard is calculated as:

$$Hazard\ Counterfactual_{c,a,s,t,d} = \frac{Hazard_{c,a,s,t,d}}{1-(d * Viral\ Suppression_{c,a,s,t,d})}$$

$$Viral\ Suppression \sim (.6,.8)$$

Where ART is the proportion of HIV+ individuals receiving ART, hazard is the number of new HIV infections over population at risk, and viral suppression is the proportion of individuals taking ART who achieve viral suppression. We assumed that a mean of 70% of the on-ART population reached viral suppression and created uncertainty by taking draws from a uniform distribution ranging from 60% - 80%, aligning with assumptions in the EPP model.

Consistent with the approach taken to forecast the independent drivers, projections for the secular trend in the counterfactual hazard are created by calculating the rate of change over the previous five years for each country. Countries were grouped by high- and low-prevalence, where the high-prevalence group was defined by peak prevalence in the past greater than 0.5%.,

The 50th percentile rate of change was taken from each group's distribution and applied to all countries in the group. The final projected hazard rates therefore decrease in response to improvements in ART coverage, as well as change due to the underlying secular trend in the counterfactual hazard.

Projections of HIV incidence

In order to produce age- and sex-specific estimates of HIV incidence, we input the projections of incidence hazard along with ART coverage, PMTCT, Cotrimoxazole coverage, and a number of other predicted demographic inputs into the Spectrum model. Spectrum is a cohort component model originally developed by UNAIDs that we have modified to incorporate CD4-specific probability of treatment in addition to a number of other methods developments made for GBD.[14, 15] Spectrum ages a population over time using demographic parameters while applying HIV incidence hazard, disease progression, CD4-specific treatment coverage, and mortality. Our final estimates of HIV incidence and ART coverage are age-, sex-, location-specific Spectrum outputs through 2030.

References

[1] Egger S, Petoumenos K, Kamarulzaman A, *et al.* Long-term patterns in CD4 response is determined by an interaction between baseline CD4 cell count, viral load and time: the Asia Pacific HIV Observational Database (APHOD). *J Acquir Immune Defic Syndr 1999* 2009; **50**: 513–20.

[2] Li T, Tubiana R, Katlama C, Calvez V, Mohand HA, Autran B. Long-lasting recovery in CD4 T-cell function and viral-load reduction after highly active antiretroviral therapy in advanced HIV-1 disease. *The Lancet* 1998; **351**: 1682–6.

[3] Mocroft A, Phillips A, Gatell J, *et al.* Normalisation of CD4 counts in patients with HIV-1 infection and maximum virological suppression who are taking combination antiretroviral therapy: an observational cohort study. *The Lancet* 2007; **370**: 407–13.

[4] Montarroyos UR, Miranda-Filho DB, César CC, *et al.* Factors Related to Changes in CD4+ T-Cell Counts over Time in Patients Living with HIV/AIDS: A Multilevel Analysis. *PLOS ONE* 2014; **9**: e84276.

[5] Smith CJ, Sabin CA, Youle MS, *et al.* Factors Influencing Increases in CD4 Cell Counts of HIV-Positive Persons Receiving Long-Term Highly Active Antiretroviral Therapy. *J Infect Dis* 2004; **190**: 1860–8.

[6] Tarwater PM, Margolick JB, Jin J, *et al.* Increase and plateau of CD4 T-cell counts in the 3(1/2) years after initiation of potent antiretroviral therapy. *J Acquir Immune Defic Syndr 1999* 2001; **27**: 168–75.

[7] Zhou J, Kumarasamy N, Ditangco R, *et al.* The TREAT Asia HIV Observational Database. *J Acquir Immune Defic Syndr 1999* 2005; **38**: 174–9.
[8] The potential for CD4 cell increases in HIV-positive individ... : AIDS.

https://journals.lww.com/aidsonline/Fulltext/2003/05020/The_potential_for_CD4_cell_increases_in.4.aspx (accessed April 17, 2018).

[9] Ferguson BD, Tandon A, Gakidou E, Murray CJ. Estimating permanent income using indicator variables. Health systems performance assessment: debates, methods and empiricism. Geneva: World Health Organization. 2003 Feb 19:747-60.

[10] WHO | Global Price Reporting Mechanism for HIV, tuberculosis and malaria [Internet]. WHO. [cited 2016 Nov 5]. Available from: http://www.who.int/hiv/amds/gprm/en/

[11] Ng M, Fleming T, Robinson M, *et al.* Global, regional, and national prevalence of overweight and obesity in children and adults during 1980-2013: a systematic analysis for the Global Burden of Disease Study 2013. *Lancet Lond Engl* 2014; **384**: 766–81.

[12] Ng M, Freeman MK, Fleming TD, *et al.* Smoking prevalence and cigarette consumption in 187 countries, 1980-2012. *JAMA* 2014; **311**: 183–92.

[13] Dieleman JL, Sadat N, Chang AY, Fullman N, Abbafati C, Acharya P, Adou AK, Kiadaliri AA, Alam K, Alizadeh-Navaei R, Alkerwi AA. Trends in future health financing and coverage: future health spending and universal health coverage in 188 countries, 2016–40. The Lancet. 2018 May 5;**391**(10132):1783-98.

[14] Stover J. Updates to the Spectrum Model to Estimate Key HIV Indicators for Adults and Children. *AIDS* 2014; : S427–S434.

[15] Vos T, Allen C, Arora M, *et al.* Global, regional, and national incidence, prevalence, and years lived with disability for 310 diseases and injuries, 1990–2015: a systematic analysis for the Global Burden of Disease Study 2015. *The Lancet* 2016; **388**: 1545–602.
