# Supplementary material for: Global, regional, and national incidence, prevalence, and mortality of HIV, 1980–2017, and forecasts to 2030, for 195 countries and territories: a systematic analysis for the Global Burden of Diseases, Injuries, and Risk Factors Study 2017
Source: Lancet HIV. 2019 Aug 19;6(12):e831–59. doi: 10.1016/S2352-3018(19)30196-1 (PMC6934077; doi:10.1016/S2352-3018(19)30196-1)

THE LANCET
HIV

Supplementary appendix

This appendix formed part of the original submission and has been peer reviewed. We post it as supplied by the authors.

Supplement to: GBD 2017 HIV collaborators. Global, regional, and national incidence, prevalence, and mortality of HIV, 1980–2017, and forecasts to 2030, for 195 countries and territories: a systematic analysis for the Global Burden of Diseases, Injuries, and Risk Factors Study 2017. *Lancet HIV* 2019; published online Aug 19. http://dx.doi.org/10.1016/S2352-3018(19)30196-1.

Supplemental Results Appendix to Global, regional, and national incidence, prevalence, and mortality of HIV from 1980 to 2017 with forecasts to 2030, for 195 countries and territories: a systematic analysis for the Global Burden of Diseases, Injuries, and Risk Factors (GBD) 2017 Study

Table of Contents

Appendix Table 1: Baseline ART Coverage (%) in 2010, and forecast ART coverage (%) in 2020 and 2030………3

Appendix Table 2. Forecast number of deaths and percent change between 2010-2020 and 2010-2030……………………………………………………………………13

Appendix Table 3. Forecast number of incident cases and percent change in incident cases between 2010-2020 and 2010-2030……………………………………………………………………19

Appendix Figure 1. Percentage of PLHIV on and off ART coverage in all GBD super-regions, by country, by sex, 2017……………………………………………………………………………………25

Appendix Figure 2. All-age rate of HIV incidence (A), prevalence (B), and mortality (C),2017…………………32

Appendix Figure 3. Percentage of new HIV infections and HIV deaths occurring in each age group in Sub−Saharan Africa, by sex, 2007 and 2017……………………………………………………………33

Appendix Table 1: Baseline ART Coverage (%) in 2010, and forecast ART coverage (%) in 2020 and 2030	ART Coverage (%)		
	Baseline (2010)	2020	2030
**Global**	25.06 (23.2;26.8)	64.83 (61.08;67.03)	71.88 (68.22;75.03)
**Central Europe, Eastern Europe, and Central Asia**			
**Central Asia**			
Armenia	45.62 (32.85;61.06)	80.8*** (70.35;84.42)	87.46 (79.94;91.01)
Azerbaijan	42.44 (30.61;56.81)	81.96*** (79.75;83.41)	87.23 (85.34;89.1)
Georgia	35.7 (20.78;60.53)	74.9 (53.46;83.23)	79.21 (62.08;85.61)
Kazakhstan	22.9 (15.85;30.51)	69.34 (58;77.87)	79.17 (69.16;86.55)
Kyrgyzstan	7.07 (4.07;11.2)	61.51 (41.91;76.16)	70.21 (51.78;82.96)
Mongolia	12.28 (5.08;26.14)	34.9 (14.69;76.69)	37.69 (18.53;78.48)
Tajikistan	10.38 (7.18;14.68)	76.14 (70.33;81.98)	80.45 (74.67;85.23)
Turkmenistan	0.31 (0.19;0.45)	2.15 (1.61;2.75)	4.97 (4.01;6.02)
Uzbekistan	12.77 (9.62;16.76)	74.85 (65.06;84.64)	81.3 (74.34;86.04)
**Central Europe**			
Albania	24.19 (20.26;28.48)	50.66 (46.77;54.24)	61.03 (56.51;65.38)
Bosnia and Herzegovina	26.83 (22.32;32.2)	49.8 (45.89;53.6)	59.1 (54.44;63.46)
Bulgaria	35.15 (25.14;44.31)	71.02 (61.25;79.21)	78.19 (69.94;84.66)
Croatia	55.08 (46.24;64.54)	83.95*** (79.94;86.89)	86.49 (83.28;88.55)
Czech Republic	72.27 (61.35;78.62)	84.1*** (79.63;87.45)	87.36 (83.02;89.56)
Hungary	52.08 (44.2;61.23)	77.53 (72.39;82.66)	79.45 (73.68;84.46)
Macedonia	48.88 (40.34;57.73)	83.37*** (82.32;84.07)	87.25 (85.69;89.02)

Montenegro	67.97 (58.39;72.33)	81.51*** (78.81;84.99)	85.63 (83.21;88.17)
Poland	83.02 (77.15;86.69)	87.09*** (85.99;89.5)	89.66*** (88.62;90.94)
Romania	70.86 (64.85;79.38)	80.87*** (74.87;86.07)	84.13 (79.22;87.92)
Serbia	51.38 (30.63;65.24)	71.23 (52.57;85.98)	74.9 (60.05;87.61)
Slovakia	68.47 (56.54;76.12)	85.69*** (84.86;86.46)	88.39 (87.64;89.59)
Slovenia	64.11 (55.35;74.25)	86.38*** (84.22;88.05)	89.64*** (87.75;91.03)
**Eastern Europe**			
Belarus	31.21 (22.47;42.18)	68.66 (54.29;80.96)	79.86 (67.95;89.68)
Estonia	69.61 (62.55;75.3)	86.25*** (85.19;87.21)	89.97*** (89.08;91.27)
Latvia	26.96 (19.05;35.35)	65.75 (54.76;77.97)	77.21 (66.98;87.64)
Lithuania	11.29 (8.51;15.17)	54.15 (40.58;73.82)	62.3 (49.47;79.78)
Moldova	20.55 (13.17;30.6)	62.66 (42.6;82.06)	71.93 (56;87.69)
Russian Federation	28.05 (18.83;38.21)	68.28 (61.38;73.19)	81.14 (75.7;85.51)
Ukraine	18.26 (12.58;24.52)	67.31 (58.67;75.47)	79.52 (72.46;85.94)
**High-income**			
**Australasia**			
Australia	83.66 (71.95;88.81)	87.21*** (83.08;88.73)	87.14 (83.1;88.58)
New Zealand	82.1 (76.34;85.71)	90.03*** (87.66;92.74)	90.53*** (88.67;92.76)
**High-income Asia-Pacific**			
Brunei	43.33 (38.47;47.8)	65.33 (59.2;70.52)	72.4 (65.02;78.49)
Japan	73.36 (68.32;77.1)	86.54*** (85.41;88.44)	88.17 (87.43;89.66)
South Korea	41.03 (25.46;57.8)	45.89 (29.78;65.01)	50.37 (34.26;68.35)
Singapore	64.17 (42.73;82.79)	76.9 (55.59;88.65)	80.67 (62.22;89.89)
**High-income North America**			

Canada	85.7 (73.95;90.86)	86.66*** (82.15;90.53)	87.62 (84.01;90.9)
Greenland	50.11 (43.55;57.15)	81.94*** (74.82;87.84)	85.74 (77.63;91.8)
USA	67.52 (62.17;72.72)	88.59*** (86.75;91.21)	89.36 (87.81;91.77)
**Southern Latin America**			
Argentina	43.34 (28.56;63.42)	51.27 (34.14;72.15)	58.73 (43.18;75.55)
Chile	51.46 (34.8;68.16)	68.95 (46.49;84.55)	75.36 (55.81;87.16)
Uruguay	30.08 (21.17;43.61)	64.98 (49.91;79.52)	70.37 (57.12;82.36)
**Western Europe**			
Andorra	74.13 (62.27;85.42)	87.89*** (85.26;91.64)	90.79*** (89.04;93.25)
Austria	82.43 (76.83;86.02)	86.56*** (85.07;87.88)	88.83 (87.48;90.17)
Belgium	83.27 (77.7;87.16)	86.45*** (84.9;88.96)	88.2 (86.71;90.21)
Cyprus	64.98 (57.9;71.99)	84.1*** (82.59;85.14)	87.93 (86.8;88.78)
Denmark	64.45 (47.94;80.13)	72.46 (62.03;86.17)	74.83 (65.05;87.26)
Finland	81.68 (76.33;85.56)	86.86*** (84.67;89.57)	87.98 (85.91;90.3)
France	69.09 (64.84;73.05)	79.12 (73.64;83.57)	80.02 (73.96;84.61)
Germany	61.05 (53.45;71.91)	85.25*** (82.6;87.26)	86.17 (84.16;87.83)
Greece	84.83 (83.71;85.58)	86.8*** (86.01;87.63)	87.73 (87.27;88.24)
Iceland	54.28 (45.98;62.8)	85.13*** (83.08;87.66)	89.4 (88.28;90.42)
Ireland	83.07 (77.65;87.13)	86.54*** (85.36;89.19)	88.2 (87.04;89.74)
Israel	78.71 (73.53;83.72)	85.15*** (82.92;86.81)	86.44 (84.42;88.18)
Italy	86.73 (82.97;88.81)	87.9*** (86.34;89.11)	88.43 (87.03;89.81)
Luxembourg	79.04 (71.94;84.52)	85.67*** (83.73;88.64)	88.78 (87.56;90.26)
Malta	56.03 (45.35;67.7)	68.67 (59.83;84.13)	75.51 (68.53;87.14)
Netherlands	70.19 (57.41;85.46)	81.72*** (75.65;88.88)	82.78 (77.4;89.77)

Norway	82.83 (77.28;86.64)	86.17*** (85.07;87.44)	88.5 (87.42;89.7)
Portugal	86.63 (85.71;88.06)	86.99*** (86.07;89.11)	89.98*** (89.22;91.38)
Spain	88.74 (85.82;90.25)	88.19*** (85.17;90.65)	89.81*** (86.48;92.2)
Sweden	73.12 (66.68;78.85)	85.26*** (81.67;88.3)	87.8 (84.16;90.5)
Switzerland	88.11 (87.31;89.67)	88.02*** (86.89;91.48)	88.22 (87.11;91.53)
United Kingdom	81.34 (76.73;85.19)	88.97*** (88.07;89.87)	90.32*** (89.61;91.04)
**Latin America and Caribbean**			
**Andean Latin America**			
Bolivia	16.82 (3.17;47.47)	50.42 (11.75;84.64)	57.83 (18.86;86.94)
Ecuador	33.67 (23.24;44.1)	57.52 (42.87;71.34)	63.71 (50.08;75.9)
Peru	39.73 (30.11;52.06)	66.43 (50.21;80.28)	72.11 (58.78;83.88)
**Caribbean**			
Antigua and Barbuda	0.04 (0.04;0.05)	1.79 (1.54;2.06)	3.09 (2.66;3.59)
The Bahamas	61.72 (49.19;71.17)	81.8*** (73.72;84.67)	87.28 (81.19;89.94)
Barbados	78.39 (75.24;80.37)	85.83*** (78.85;88.83)	88.99 (83.82;91.3)
Belize	42.09 (31.72;54.27)	53.99 (43.97;64.59)	60.82 (52.2;69.96)
Bermuda	0.05 (0.04;0.06)	1.5 (1.37;1.65)	3.09 (2.7;3.49)
Cuba	49.7 (35.5;71.23)	70.61 (53.56;83.18)	77.73 (62.82;88.69)
Dominica	0.04 (0.03;0.05)	1.92 (1.6;2.27)	3.44 (2.91;4.01)
Dominican Republic	23.54 (18.07;29.91)	51.31 (39.15;63.54)	60.87 (49.51;71.64)
Grenada	0.04 (0.03;0.05)	1.88 (1.63;2.15)	3.5 (3.13;3.9)
Guyana	35.47 (30.92;41.94)	55.9 (49.84;62.95)	61.51 (55.59;68.18)
Haiti	19.68 (17.2;22.47)	62.71 (55.58;70.54)	71.35 (65.37;77.62)
Jamaica	49.08 (39.49;56.72)	77.98 (65.21;84.69)	83.36 (73.07;88.55)

Puerto Rico	0.05 (0.04;0.06)	1.42 (1.3;1.54)	3 (2.66;3.38)
Saint Lucia	0.04 (0.04;0.05)	1.6 (1.4;1.82)	3.01 (2.62;3.44)
Saint Vincent and the Grenadines	0.04 (0.04;0.05)	1.77 (1.51;2.03)	3.35 (2.87;3.86)
Suriname	36.13 (29.38;43.22)	76.32 (65;83.04)	81.85 (73.02;87.24)
Trinidad and Tobago	60.58 (51.17;66.53)	82.52*** (73.93;86.27)	88.1 (82.09;90.83)
Virgin Islands	0.04 (0.04;0.05)	1.59 (1.4;1.78)	3.26 (2.88;3.71)
**Central Latin America**			
Colombia	35.26 (25.18;46.72)	77.86 (64.61;83.71)	83.35 (73.07;87.98)
Costa Rica	57.81 (44.06;68.38)	83.93*** (73.2;87.45)	86.57 (78.56;88.81)
El Salvador	44.55 (37.88;52.96)	58 (49.92;68.12)	63.56 (55.44;73.58)
Guatemala	55.39 (35.36;74.66)	61.92 (39.9;78.49)	63.68 (44.6;79.09)
Honduras	82.63 (80.97;83.82)	85.25*** (83.88;86.84)	87.3 (85.79;88.49)
Mexico	46.45 (35.55;58.36)	65.86 (53.5;78.28)	71.03 (61.08;81.23)
Nicaragua	20.41 (15.51;26.9)	34.37 (27.92;42.81)	45.29 (37.99;54.63)
Panama	47.6 (37.32;56.67)	67.19 (52.35;79.68)	76.79 (65.09;85.72)
Venezuela	62.03 (50.14;72.75)	80.67*** (72.09;83.92)	84.16 (78.06;86.45)
**Tropical Latin America**			
Brazil	47 (40.79;53.56)	57.12 (50.73;65.24)	63.03 (57.32;71.46)
Paraguay	27.4 (21.95;34.83)	46.15 (38.11;55.68)	51.75 (43.85;62.67)
**North Africa and Middle East**			
Afghanistan	4.64 (0.47;14.82)	33.52 (6.28;82.8)	42.46 (11.28;88.24)
Algeria	42.79 (7.97;80)	75.46 (37;93.27)	79.7 (47.78;96.85)
Bahrain	32.26 (23.65;41.08)	54.13 (40.94;65.39)	57.28 (44.23;67.81)
Egypt	23.6 (16.63;30.43)	71.97 (61.47;78.62)	77.88 (68.34;83.37)

Iran	19.74<br>(14.05;25.64)	48.26<br>(41.53;54.87)	57.66<br>(50.81;64.77)
Iraq	0.02<br>(0.02;0.03)	8.5<br>(6.82;10.31)	14.13<br>(11.2;17.24)
Jordan	38.82<br>(31.85;47.84)	47.95<br>(36.96;58.83)	51.24<br>(40.2;63.49)
Kuwait	77.92<br>(72.59;83.74)	84.6***<br>(80.38;86.01)	84.74<br>(81.07;86.07)
Lebanon	53.47<br>(12;83.78)	59.75<br>(19.08;84.31)	66.69<br>(25.78;88.48)
Libya	0.01<br>(0.01;0.02)	5.24<br>(2.52;8.45)	9.98<br>(5.74;14.59)
Morocco	40.18<br>(6.22;83.61)	73.85<br>(29.61;90.55)	77.68<br>(39.04;91.61)
Palestine	0.02<br>(0.02;0.03)	8.15<br>(6.87;9.19)	13.83<br>(12.06;15.66)
Oman	42.41<br>(33.68;51.36)	42.75<br>(28.04;65.39)	47.56<br>(33.64;70.35)
Qatar	58.08<br>(50.63;64.39)	56.39<br>(46.6;63.55)	56.89<br>(46.99;64.33)
Saudi Arabia	0.02<br>(0.01;0.02)	3.97<br>(2.3;5.75)	6.35<br>(4.68;8.34)
Sudan	2.82<br>(2.36;3.34)	15.1<br>(11.28;18.86)	23.47<br>(18.64;28.7)
Syria	16.22<br>(9.78;21.63)	61.96<br>(41.26;74.19)	72.71<br>(55.16;81.68)
Tunisia	31.4<br>(5.81;79.56)	46.14<br>(11.74;85.82)	54.54<br>(17.44;91.35)
Turkey	46.09<br>(36.87;52.96)	47.56<br>(33.96;59.62)	55.64<br>(42.27;66.87)
United Arab Emirates	0.01<br>(0;0.02)	2.24<br>(1.25;4.34)	3.31<br>(2.09;7.2)
Yemen	25.1<br>(2.77;73.24)	50.81<br>(10.24;86.45)	57.6<br>(15.02;91.35)
**South Asia**			
Bangladesh	19.44<br>(1.72;76.86)	43.17<br>(8.92;85.55)	50.51<br>(12.95;89.73)
Bhutan	8.31<br>(0.63;48.01)	12.49<br>(2.15;55.73)	17.01<br>(4.21;61.54)
India	21.76<br>(19.17;24.21)	73.81<br>(66.61;79.14)	73.88<br>(66.07;79.4)
Nepal	24.33<br>(2.15;86.1)	55.18<br>(14.45;89.88)	57.85<br>(17.46;89.81)
Pakistan	13.31<br>(1.11;64.58)	30.36<br>(4.61;78.03)	38.53<br>(7.85;84.8)

**Southeast Asia, East Asia, and Oceania**			
**East Asia**			
China	29.04 (23.31;34.49)	38.78 (33.41;48.4)	45.35 (40.12;56.1)
North Korea	29.63 (16.85;44.93)	34.89 (14.88;59.2)	40.14 (21.82;65.2)
Taiwan (Province of China)	29.9 (22.45;37.06)	42.52 (35.1;51.73)	54.66 (45.9;65.73)
**Oceania**			
American Samoa	0 (0;0)	0.96 (0.92;1.02)	1.57 (1.43;1.77)
Federated States of Micronesia	0 (0;0)	1.07 (1;1.15)	1.99 (1.69;2.44)
Fiji	27.77 (21.1;33.21)	56.51 (47.2;64.51)	64.67 (55.73;72.29)
Guam	0 (0;0)	1 (0.97;1.04)	1.64 (1.51;1.85)
Kiribati	0 (0;0)	0.89 (0.87;0.91)	1.32 (1.21;1.48)
Marshall Islands	0 (0;0)	0.95 (0.88;1.04)	1.54 (1.34;1.81)
Northern Mariana Islands	0 (0;0)	1.03 (0.99;1.09)	2.12 (1.85;2.45)
Papua New Guinea	22.23 (7.02;76.88)	69.6 (29.96;89.8)	73.77 (37.57;90.38)
Samoa	0 (0;0)	0.93 (0.84;1.02)	1.47 (1.23;1.8)
Solomon Islands	0 (0;0)	0.92 (0.84;1.01)	1.42 (1.23;1.67)
Tonga	0 (0;0)	0.94 (0.9;1)	1.47 (1.35;1.65)
Vanuatu	0 (0;0)	0.94 (0.85;1.05)	1.54 (1.3;2.01)
**Southeast Asia**			
Cambodia	46.3 (36.47;61.92)	77.74 (62.6;89.18)	85.08 (70.85;94.8)
Indonesia	20.89 (14.53;27.07)	50.72 (39.16;61.13)	59.17 (44.51;70.92)
Laos	44.74 (3.09;85.73)	66.98 (13.44;89.91)	71.03 (19.69;90.19)
Malaysia	35.08 (28.22;42.22)	79.27 (69.92;83.88)	83.23 (74.5;87.53)
Maldives	28.25 (22.86;32.78)	31.98 (23;40.13)	39.58 (32.2;46.08)

Mauritius	35.83 (26.73;46.45)	83.36*** (74.6;86.22)	86.71 (80.77;89.15)
Myanmar	11.63 (10.2;13.23)	69.62 (63.22;76.41)	74.05 (68.19;79.76)
Philippines	1.72 (0.98;2.56)	24.37 (13.26;38.39)	34.24 (20.81;49.71)
Sri Lanka	21.18 (14.48;28.05)	62.56 (53.34;72.19)	70.58 (62.33;79.06)
Seychelles	0.02 (0.01;0.02)	2.7 (2.2;3.26)	8.17 (7.37;8.97)
Thailand	41.01 (36.19;46.49)	67.72 (59.26;73.68)	73.75 (67.18;78.92)
Timor-Leste	0.01 (0.01;0.03)	7.04 (2.72;14.42)	14.08 (7.47;28.52)
Vietnam	20.29 (14.38;25.53)	42.23 (31.81;50.23)	51.29 (41.01;58.63)
**Sub-Saharan Africa**			
**Central sub-Saharan Africa**			
Angola	8.96 (6.83;11.5)	30.75 (24.54;37.76)	40.18 (32.39;50.15)
Central African Republic	8.85 (7.75;10.05)	33.97 (26.35;43.5)	43.77 (33.72;58.09)
Congo (Brazzaville)	16.16 (13.34;19.18)	31.94 (26.75;37.85)	39.48 (33.6;45.89)
DR Congo	7.64 (6.39;9.01)	53.02 (42.66;63.17)	63.3 (51.05;76.61)
Equatorial Guinea	10.88 (9.21;12.74)	46.67 (33.95;59.22)	59.48 (45.66;72.21)
Gabon	27.14 (22.06;32.88)	84.83*** (75.52;90.38)	88.29 (81.67;93.09)
**Eastern sub-Saharan Africa**			
Burundi	17.25 (14.71;19.7)	65.35 (51.96;77.09)	70.41 (56.37;83.22)
Comoros	63.36 (10.11;91.44)	63.75 (14.74;81.54)	70.51 (20.23;86.54)
Djibouti	8.58 (5.95;11.84)	29.11 (19.17;42.53)	39.28 (27.84;53.82)
Eritrea	12.61 (10.29;15.22)	37.09 (27.59;48.26)	46.73 (35.07;61.93)
Ethiopia	27.77 (23.44;32.11)	80.74*** (68.8;89.15)	81.34 (69.2;91.35)
Kenya	24.48 (22.16;26.83)	56.93 (48.85;62.95)	65.9 (58.78;70.87)
Madagascar	0.71 (0.58;0.85)	10.12 (4.24;16.48)	17.86 (11.6;26.33)

Malawi	22.21 (19.97;24.48)	72.19 (65.16;78.52)	79.82 (72.7;86.5)
Mozambique	11.81 (10.45;13.18)	54.78 (46.8;63.78)	64.52 (57;72.51)
Rwanda	44.1 (39.64;48.54)	81.98*** (73.7;87.98)	85.4 (78.32;90.26)
Somalia	3.49 (0.53;12.13)	26.5 (5.01;69.72)	33.4 (10.64;74.09)
South Sudan	5.21 (0.72;24.85)	47.37 (12.68;89.13)	57.12 (23.51;90.58)
Tanzania	16.27 (13.35;19.72)	73.74 (60.08;85.1)	80.77 (67.46;92.68)
Uganda	19.55 (17.2;22.47)	78 (67.17;84.54)	84.1 (75.05;91.72)
Zambia	31.54 (28.69;34.27)	86.63*** (78.72;92.37)	91.75*** (85.5;96.33)
**Southern sub-Saharan Africa**			
Botswana	49.49 (45.08;53.91)	87.42*** (83.04;90.89)	90.82*** (87.68;93.5)
Lesotho	27.34 (24.91;29.68)	70.91 (66.28;75.49)	82.03 (79.37;84.59)
Namibia	37.4 (33.66;41.18)	81.23*** (73.35;88.57)	90.01*** (84.55;95.26)
South Africa	17.86 (16.64;19.13)	72.76 (67.93;77.93)	84.71 (78.92;90.43)
Swaziland	26.97 (24.77;29.34)	94.47*** (89.28;97.49)	97.36*** (93.88;99.35)
Zimbabwe	25.16 (20.96;29.99)	85.43*** (76.29;92.83)	88.55 (80.79;97.26)
**Western sub-Saharan Africa**			
Benin	31.03 (26.62;36.19)	60.09 (51.04;69.98)	66.42 (57.19;76.85)
Burkina Faso	28.03 (23.49;34.21)	68.61 (56.88;78.83)	74.28 (61.37;87.92)
Cameroon	14.97 (13.38;16.7)	43.52 (36.74;51.62)	53.4 (45.46;64.39)
Cape Verde	16.01 (12.3;20.24)	45.28 (35.44;55.65)	55.85 (45.76;66.29)
Chad	23.26 (17.34;29.83)	47.26 (37.6;57.75)	54.87 (44.68;66.53)
Cote d'Ivoire	18.7 (14.02;25.86)	47.15 (36.44;61.98)	55.87 (44.89;74.12)
The Gambia	8.04 (6.12;10.16)	35.96 (27.51;46.44)	45.18 (35.24;59.19)
Ghana	11.22 (9.57;13)	39.98 (33.17;47.38)	50.79 (43.36;58.75)

Guinea	18.97 (15.85;22.72)	57.75 (46.62;71.46)	63.01 (51.68;75.67)
Guinea-Bissau	9.14 (7.2;11.47)	41.35 (31.12;54.36)	49.35 (37.8;64.13)
Liberia	9.98 (8.39;11.59)	31.86 (24.2;40.4)	37.5 (29.16;47.75)
Mali	21.31 (18.29;25.09)	42.3 (36.07;49.36)	47.93 (40.89;55.43)
Mauritania	79.76 (35.98;89.89)	80.82*** (45.12;90.06)	81.93 (48.89;91.44)
Niger	12.93 (10.91;15.13)	41.83 (34.75;49.27)	46.83 (39.07;54.98)
Nigeria	14.26 (12.71;15.74)	38.41 (33.27;43.43)	46.56 (40.47;52.8)
Sao Tome and Principe	87.69 (83.94;91.37)	83.86*** (79.02;89.83)	83.24 (79.26;88.74)
Senegal	23.51 (19.6;27.94)	48.39 (40.49;58.1)	51.68 (43.62;60.9)
Sierra Leone	8.39 (6.97;10.11)	40.34 (31.24;53.38)	49.75 (38.76;67.7)
Togo	13.95 (11.38;16.88)	59.65 (48.94;70.51)	68.16 (56.42;82.73)

Appendix Table 2: Forecast number of deaths and percent change between 2010-2020 and 2010-2030

	2010 (95% UI)	2020 (95% UI)		2030 (95% UI)	
	Number of cases	Number of cases	Percent Change	Number of cases	Percent Change
**Central Europe, Eastern Europe, and Central Asia**					
**Central Asia**					
Armenia	11.76 (11.16;12.38)	24.68 (20.04;28.68)	109.8 (72.06;145.92)	33.38 (24.8;42.32)	183.77 (108.02;265.01)
Azerbaijan	43.96 (33.55;56.63)	38.09 (28.77;45.82)	-13.35 (-40.11;29.25)	55.77 (33.95;72.79)	26.86 (-23.85;96.86)
Georgia	14.48 (13.81;15.16)	38.68 (11.66;47.16)	167.15 (-20.88;231.21)	57.11 (18.11;73.25)	294.41 (25.93;412.24)
Kazakhstan	303.99 (293.24;315.3)	287.15 (237.89;335.52)	-5.54 (-21.74;10.63)	510.62 (292.61;710.2)	67.97 (-3.23;132.6)
Kyrgyzstan	123.97 (119.23;128.59)	191.82 (161.97;222.85)	54.73 (31;80.35)	261.88 (200.51;341.68)	111.24 (60.74;177.87)
Mongolia	8.46 (3.81;14.47)	25.49 (5.48;51.77)	201.3 (17.98;355.08)	41.39 (11.61;84.48)	389.24 (123.71;805.95)
Tajikistan	113.47 (100.42;126.6)	64.7 (43.69;88.03)	-42.98 (-62.37;-17.11)	101.39 (52.19;153.67)	-10.65 (-55.87;42.39)
Turkmenistan	120.65 (115.41;126.15)	108.44 (101.79;115.46)	-10.12 (-16.56;-2.65)	155.92 (133.4;185.91)	29.23 (9.59;56.63)
Uzbekistan	536.49 (518.24;554.68)	626.2 (532.11;738.82)	16.72 (-2.15;37.41)	809.26 (536.42;1141.11)	50.84 (0.11;111.96)
**Central Europe**					
Albania	1.79 (1.69;1.89)	1.14 (0.94;1.38)	-36.34 (-46.37;-25.34)	1.31 (0.97;1.63)	-26.46 (-45.42;-10.95)
Bosnia and Herzegovina	2.26 (2.03;2.49)	1.37 (1.16;1.61)	-39.35 (-47.8;-30.9)	1.62 (1.32;1.92)	-27.99 (-41.13;-16.7)
Bulgaria	72.83 (70.15;75.82)	44.22 (38.75;49.85)	-39.27 (-47.09;-31.12)	41.91 (26.82;54.95)	-42.46 (-63.3;-23.79)
Croatia	12.6 (12.12;13.1)	8.17 (6.77;9.17)	-35.19 (-46.55;-26.73)	8.15 (4.87;10.56)	-35.29 (-61.26;-15.77)
Czech Republic	18.6 (17.9;19.31)	23.42 (20.93;26.1)	25.92 (11.24;41.14)	25.63 (19.8;30.76)	37.76 (6.19;66.19)
Hungary	66.93 (64.05;70.08)	32.41 (25.44;39.57)	-51.59 (-62.31;-40.49)	36.1 (19.18;53.23)	-46.06 (-71.56;-19.96)
Macedonia	2.01 (1.65;2.43)	1.61 (1.26;1.93)	-19.65 (-33.57;-6.29)	1.66 (1.17;2.07)	-17.51 (-39.6;2.39)
Montenegro	1.45 (1.2;1.73)	1.07 (0.78;1.41)	-26.25 (-47.67;-3.09)	1.05 (0.72;1.44)	-27.49 (-51.25;-0.35)
Poland	168.51 (162.78;174.8)	141.23 (121.67;156.99)	-16.19 (-28.13;-6.05)	137.19 (94.99;166.72)	-18.59 (-43.67;0.04)
Romania	111.42 (107.66;115.04)	109.62 (68.14;164.69)	-1.62 (-39.37;48.33)	103.84 (57.97;187.52)	-6.8 (-48.34;66.9)
Serbia	62.72 (41.5;139.8)	56.27 (14.3;140.92)	-10.28 (-69.53;60.29)	48.92 (8.06;129.08)	-22.01 (-81.7;48.95)
Slovakia	3.85 (3.21;4.51)	3.96 (2.7;5.12)	2.72 (-25.11;21.44)	4.39 (2.42;5.92)	13.87 (-31.6;42.97)
Slovenia	1.3 (1.24;1.35)	2.22 (1.76;2.52)	71.69 (35.21;95.88)	2.17 (1.3;2.88)	67.17 (1.01;122.88)
**Eastern Europe**					
Belarus	360.07 (345.92;373.97)	314.93 (235.14;390.55)	-12.54 (-34.63;8.58)	535.67 (330.97;888.68)	48.77 (-8.81;146.82)
Estonia	42.23 (40;44.67)	36.78 (32.35;40.07)	-12.91 (-23.66;-3.53)	34.89 (22.63;41.43)	-17.39 (-46.35;-1.08)
Latvia	127.6 (122.62;132.6)	112.76 (102.27;125.25)	-11.63 (-20.5;-0.68)	136.08 (100.28;164.04)	6.64 (-21.34;29)
Lithuania	86.8 (82.82;90.94)	54.06 (43.96;63.29)	-37.71 (-49.85;-25.95)	52.58 (26.98;69.96)	-39.42 (-68.9;-18.99)
Moldova	216.06 (206.32;225.7)	148.52 (110.61;179.97)	-31.26 (-49.2;-16.12)	206.14 (158.83;261.64)	-4.59 (-26.45;21.4)
Russian Federation	12189.47 (12111.77;12268)	21794.62 (19635.27;24520.3)	78.8 (60.73;101.26)	38904.42 (30026.56;49570.05)	219.16 (146.28;307.44)
Ukraine	8611.58 (8429.82;8793.75)	4639.95 (3714.91;5538.06)	-46.12 (-57.02;-35.69)	6871.41 (3845.82;8900)	-20.21 (-55.45;3.32)
**High-income**					
**Australasia**					
Australia	85.59 (83.19;88.27)	75.68 (70.89;79.75)	-11.58 (-17.96;-6.08)	85.27 (76.79;93.74)	-0.38 (-11.1;9.91)
New Zealand	15.93 (15.39;16.48)	9.31 (8.04;10.41)	-41.55 (-49.48;-34.37)	11.88 (8.57;14.34)	-25.42 (-46.01;-9.27)
**High-income Asia-Pacific**					
Brunei	4.96 (4.56;5.32)	4.7 (3.87;5.53)	-5.25 (-20.57;9.73)	5.39 (3.94;6.8)	8.61 (-20.12;34.56)

Japan	237.09 (233.7;240.52)	198.64 (194.26;202.88)	-16.22 (-18.55;-14.1)	193.44 (182.33;201.94)	-18.41 (-23.58;-14.52)
South Korea	157.77 (150.21;166.41)	142.87 (75.07;201.09)	-9.44 (-52.91;28.55)	151.95 (25.51;309.7)	-3.69 (-84.38;96.34)
Singapore	35.26 (32.95;37.53)	23.07 (14.36;35.8)	-34.57 (-59.91;1.18)	20.34 (11.32;41.02)	-42.3 (-68.22;17.05)
**High-income North America**					
Canada	334.77 (322.51;347.76)	263.77 (214.03;293.95)	-21.21 (-35.83;-11.48)	317.33 (228.68;375.33)	-5.21 (-32.2;12.11)
Greenland	2.79 (2.44;3.07)	1.51 (1;2.03)	-45.84 (-63.96;-29.78)	1.55 (0.95;2.23)	-44.7 (-65.4;-20.5)
USA	9065.41 (8997.77;9134.48)	6885.81 (6121.15;7475.24)	-24.04 (-32.3;-17.47)	6162.75 (4110.4;7977.87)	-32.02 (-54.69;-11.95)
**Southern Latin America**					
Argentina	1569.32 (1543.54;1597.42)	1228.1 (1061.2;1479.11)	-21.74 (-32.37;-6.05)	1006.19 (735.02;1517.93)	-35.88 (-53.36;-3.19)
Chile	455.1 (445.74;463.84)	436.75 (341.41;539.42)	-4.03 (-25.22;18.85)	455.29 (317.78;629.55)	0.04 (-30.74;38.04)
Uruguay	194.55 (190.63;198.81)	155.71 (139.41;177.51)	-19.97 (-28.52;-8.58)	155.08 (119.85;197.65)	-20.29 (-38.53;1.27)
**Western Europe**					
Andorra	0.91 (0.09;3.86)	0.45 (0.06;1.93)	-50.09 (-62.83;-19.43)	0.42 (0.04;1.9)	-53.38 (-72.1;-20.83)
Austria	45.8 (43.79;47.88)	39.12 (33.68;43.98)	-14.6 (-27.51;-2.68)	43.27 (33.92;52.39)	-5.53 (-26.2;14.18)
Belgium	84.85 (81.84;88.11)	62.6 (56.79;67.45)	-26.23 (-33.36;-19.53)	64.99 (51.77;73.97)	-23.41 (-39.81;-12.52)
Cyprus	2.43 (2.14;2.71)	2.08 (1.8;2.36)	-14.49 (-29.21;2.48)	2.64 (2.2;3.08)	8.41 (-13.02;32.64)
Denmark	42.25 (40.46;44.05)	20.29 (11.6;29.26)	-51.98 (-72.54;-30.18)	23.42 (13.94;33.12)	-44.56 (-66.61;-21.96)
Finland	12.32 (11.77;12.88)	6.21 (5.44;6.82)	-49.6 (-56.21;-44.36)	6.2 (4.56;7.32)	-49.67 (-63.45;-40.08)
France	592.8 (574.81;609.68)	270.47 (158.58;415.98)	-54.37 (-73.3;-29.85)	236.13 (155.41;362.06)	-60.17 (-74.05;-39.08)
Germany	574.01 (554.65;591.42)	466.18 (416.33;499.17)	-18.78 (-27.55;-12.13)	498.86 (409.37;568.37)	-13.09 (-28.54;-0.37)
Greece	23.04 (22.03;24.09)	30.42 (28.82;32.06)	32.04 (22.6;42)	31.49 (28.77;36.51)	36.67 (22.94;59.06)
Iceland	1.15 (1.09;1.21)	0.96 (0.83;1.08)	-16.14 (-28.38;-4.59)	1.25 (0.89;1.53)	8.38 (-21.62;33.6)
Ireland	13.49 (12.86;14.19)	9.47 (8.01;10.81)	-29.77 (-41.26;-19.51)	9.84 (6.61;12.24)	-27.04 (-52.33;-8.32)
Israel	33.52 (32.26;34.86)	46.09 (41.69;50.76)	37.5 (24.26;52.02)	56.26 (47.8;63.6)	67.84 (42.26;90.98)
Italy	833.98 (809.97;856.6)	638.34 (581.48;690.36)	-23.46 (-30.46;-16.55)	669.93 (579.83;769.6)	-19.67 (-30.59;-7.26)
Luxembourg	2.61 (2.49;2.74)	2.6 (2.21;2.86)	-0.39 (-15.79;11.01)	3.04 (2.22;3.54)	16.24 (-15.15;37.06)
Malta	1.9 (1.81;1.99)	1.28 (0.91;1.82)	-32.49 (-52.85;-3.43)	1.58 (1.17;2.19)	-17.18 (-39.33;15.53)
Netherlands	61.89 (59.3;64.5)	43.14 (21.11;56.5)	-30.29 (-66.17;-8.49)	37.22 (12.02;55.22)	-39.86 (-80.69;-10.32)
Norway	16.96 (16.61;17.33)	14.91 (13.42;16.1)	-12.1 (-20.92;-4.62)	16.71 (13.48;18.98)	-1.5 (-20.07;12.39)
Portugal	714.06 (690.42;737.83)	488.89 (440.82;522.5)	-31.53 (-38.86;-26.29)	453.12 (367.48;508.45)	-36.54 (-49.13;-28.49)
Spain	1050.85 (1016.79;1086.3)	467.74 (264.51;694.08)	-55.49 (-74.92;-33.57)	365.2 (204.08;589.65)	-65.25 (-80.61;-43.32)
Sweden	25.29 (24.61;26.01)	23.09 (19.95;25.6)	-8.73 (-21.06;1.65)	24.9 (18.47;29.26)	-1.54 (-27.04;16.2)
Switzerland	47.83 (45.74;49.99)	32.59 (26.37;36.47)	-31.85 (-44.95;-22.62)	31.9 (20.7;38.67)	-33.31 (-56.59;-18.81)
United Kingdom	342.74 (340.87;344.99)	255.09 (237.16;268.68)	-25.57 (-30.82;-21.55)	284.7 (255.74;307.9)	-16.94 (-25.47;-10.15)
**Latin America and Caribbean**					
**Andean Latin America**					
Bolivia	939.36 (79.52;3507.03)	1293.4 (50.67;6106.27)	37.69 (-72.33;170.06)	1712.49 (69.24;7477.4)	82.3 (-65.88;647.35)
Ecuador	958.35 (928.92;990.42)	1060.12 (973.88;1149.66)	10.62 (0.72;20.74)	1345.87 (1205.42;1506.12)	40.44 (25.03;57.61)
Peru	1518.11 (1320.27;1825.04)	2333.02 (1113.88;3693.53)	53.68 (-21.56;116.4)	2586.33 (1212.43;4177.76)	70.37 (-13.53;147.15)
**Caribbean**					
Antigua and Barbuda	7.6 (7.45;7.73)	7.87 (7.57;8.25)	3.6 (-0.58;8.83)	10.08 (8.99;11.75)	32.65 (18.36;55.38)
The Bahamas	116.17 (114.06;118.46)	100.81 (87.11;120.04)	-13.23 (-24.98;3.36)	110.27 (79.29;141.79)	-5.09 (-31.52;21.48)
Barbados	30.1 (29.51;30.7)	20.36 (16.84;23.65)	-32.33 (-44.04;-21.17)	16.73 (11.51;21.58)	-44.41 (-61.53;-27.71)
Belize	75.47 (74.13;76.85)	81.58 (74.26;90.29)	8.08 (-2.1;19.8)	108.51 (86.27;124.8)	43.78 (14.67;65.75)
Bermuda	7.22 (7.08;7.36)	6.82 (6.59;7.08)	-5.45 (-9.02;-1.28)	8.26 (7.52;9.15)	14.39 (3.87;27.29)

Cuba	226.67 (221.9;231.46)	411.89 (343.02;500.66)	81.71 (51.26;121.66)	519.94 (389.45;773.75)	129.38 (70.54;246.1)
Dominica	5.16 (5.06;5.26)	5.33 (5.02;5.81)	3.31 (-3.17;12.84)	6.59 (5.65;8.34)	27.69 (8.98;61.81)
Dominican Republic	3430.14 (2607.57;4594.14)	2233.97 (1389.59;3365.06)	-34.87 (-50.65;-21.63)	1814.65 (756.06;3206.28)	-47.1 (-73.06;-25.35)
Grenada	6.48 (6.36;6.61)	6.26 (6.05;6.51)	-3.49 (-7.44;0.76)	7.38 (6.52;8.35)	13.87 (0.46;28.7)
Guyana	216.36 (212.58;220.59)	176.17 (146.95;208.78)	-18.58 (-32.29;-3.55)	176.45 (132.29;232.07)	-18.45 (-38.85;7.3)
Haiti	8076.39 (6776.91;9867.04)	3514.27 (2681.38;4527.81)	-56.49 (-63.63;-48.52)	2943.66 (1962.82;4165.46)	-63.55 (-74;-52.43)
Jamaica	611.79 (601.85;621.95)	411.25 (355.66;453.31)	-32.78 (-41.66;-25.73)	421.82 (329.75;478.2)	-31.05 (-46.15;-21.55)
Puerto Rico	358.72 (352.28;365.46)	213.71 (181.77;232.34)	-40.43 (-49.15;-35.07)	227.2 (143.16;273.55)	-36.67 (-59.81;-23.19)
Saint Lucia	8.12 (7.97;8.26)	7.87 (7.58;8.22)	-3.12 (-7.31;1.53)	9.36 (8.32;10.74)	15.29 (2.64;31.8)
Saint Vincent and the Grenadines	20.89 (20.49;21.3)	19.83 (19.05;20.73)	-5.05 (-9.15;-0.35)	21.4 (19.14;24.33)	2.44 (-8.63;16.09)
Suriname	120.34 (118.18;122.54)	83.6 (74.41;91.92)	-30.53 (-38.52;-23.6)	66.21 (47.93;88.42)	-44.98 (-60.48;-26.42)
Trinidad and Tobago	226.68 (222.87;230.86)	164.51 (149.01;180.27)	-27.43 (-34.12;-20.48)	117.51 (87.44;149.55)	-48.16 (-61.56;-34.05)
Virgin Islands	10.01 (9.83;10.19)	9.78 (8.75;11.08)	-2.35 (-13.08;11.18)	12.62 (9.61;17.15)	26.08 (-3.79;71.09)
**Central Latin America**					
Colombia	2797.59 (2733.2;2864.6)	2659.99 (2276.16;2917.04)	-4.92 (-18.54;4.62)	3103.18 (2424.88;3515.17)	10.92 (-13.34;25.53)
Costa Rica	157.05 (151.3;163.5)	138.78 (111.41;157.29)	-11.63 (-29.13;1.41)	134.85 (78.65;169.87)	-14.13 (-50.7;9.35)
El Salvador	665.93 (457.91;904.55)	722.66 (304.79;1061.45)	8.52 (-34.87;33.98)	682.7 (217.8;1070.03)	2.52 (-52.8;33.74)
Guatemala	756.27 (735.96;775.05)	739.13 (616.92;843.04)	-2.27 (-18.87;12.24)	973.61 (606.36;1359.68)	28.74 (-19.44;80.41)
Honduras	86.17 (71.55;108.69)	78.02 (58.67;101.79)	-9.46 (-29.62;10.89)	88.54 (60.93;122.23)	2.75 (-27.45;35.76)
Mexico	5734.4 (5687.89;5782.66)	5692.38 (5364.49;6078.03)	-0.73 (-6.47;5.98)	7413.67 (6476.18;8048.81)	29.28 (13.02;40.46)
Nicaragua	269.79 (192.22;333.76)	700.7 (411.13;978.62)	159.72 (90.1;248.89)	1036.59 (658.02;1469.39)	284.22 (171.89;430.33)
Panama	550.87 (532.91;567.68)	457.25 (414.29;508.66)	-17 (-25.34;-7.09)	512.39 (440.88;601.32)	-6.99 (-20.79;10.38)
Venezuela	1938.11 (1892.25;1985.88)	2079.13 (1608.82;2348.36)	7.28 (-17.59;21.93)	2404.36 (1497.02;2906.45)	24.06 (-23.59;51.05)
**Tropical Latin America**					
Brazil	15274.42 (15125.54;15420.65)	15166.73 (14040.07;16700.74)	-0.71 (-7.91;9.58)	20953.11 (15334.27;23966.54)	37.18 (0.17;57.3)
Paraguay	464.91 (300;590.44)	796.92 (427;1117.66)	71.41 (27.21;116.8)	980.35 (511.08;1385.49)	110.87 (52.27;174.57)
**North Africa and Middle East**					
Afghanistan	169.04 (15.05;691.79)	426.11 (10.59;1859.85)	152.08 (-65.27;654.57)	735.5 (20.08;2803.24)	335.12 (-41.08;2918.83)
Algeria	355.24 (16.99;1926.41)	356.2 (19.09;2017.98)	0.27 (-73.54;136.92)	451.83 (11.11;2416.23)	27.19 (-83.34;638.43)
Bahrain	13.92 (12.71;14.9)	11.07 (8.89;13.35)	-20.44 (-31.83;-3.49)	15.98 (11.68;20.3)	14.82 (-12.4;44.9)
Egypt	104.44 (90.7;118.47)	71.5 (50.11;101.16)	-31.54 (-46.12;-12.47)	101.98 (64.71;148.88)	-2.36 (-33.54;31.6)
Iran	452.24 (430.97;473.58)	892.01 (765.35;1018.78)	97.24 (72.27;122.6)	1448.24 (1078.97;1787.89)	220.23 (140.69;297.35)
Iraq	74.53 (61.12;95.48)	128.95 (90.47;187.96)	73.03 (35.91;114.31)	196.58 (129.76;296.79)	163.77 (90.53;267.18)
Jordan	14.27 (12.49;16)	23.88 (17.13;31.06)	67.39 (24.11;106.89)	29.45 (19.39;39.59)	106.37 (38.14;167.32)
Kuwait	4.84 (4.31;5.49)	5.07 (4.41;5.82)	4.74 (-12.74;25.64)	5.65 (4.81;6.66)	16.74 (-4.91;42.29)
Lebanon	88.76 (4.61;426.32)	130.4 (4.13;631.43)	46.92 (-50.45;310.14)	131.92 (4.11;596.29)	48.63 (-58.77;890.78)
Libya	70.13 (7.53;299.75)	122.86 (11.16;466.47)	75.19 (-17.68;392.06)	172.7 (13.5;562.18)	146.25 (-12.08;1307.96)
Morocco	889.73 (27.01;4608.83)	627.98 (15.21;3930.67)	-29.42 (-83.21;50.18)	714.44 (12.56;4503.98)	-19.7 (-85.9;189.37)
Palestine	8.52 (7.73;9.26)	11.58 (9.66;13.71)	35.85 (21.14;51.83)	17.61 (13.97;22.42)	106.63 (70.61;160.78)
Oman	61.72 (35.22;82.69)	167.73 (87.2;247.73)	171.75 (22.57;271.49)	350.67 (185.67;560.14)	468.16 (164.43;981.76)
Qatar	6.47 (5.23;7.38)	6.19 (4.82;7.43)	-4.2 (-16.04;9.48)	7.62 (5.91;9.58)	17.8 (-1.73;41.58)
Saudi Arabia	419.63 (363.84;513.4)	558.22 (412.25;721.93)	33.03 (3.27;68.21)	732.97 (497.94;1086.52)	74.67 (20.67;163.95)
Sudan	4628.5 (3565.73;5971.88)	5212.84 (3797.35;6998.14)	12.62 (-8.89;36.98)	5357.18 (2660.22;8672.96)	15.74 (-37.79;81.15)
Syria	21.96 (18.76;26.92)	10.77 (7.5;17.4)	-50.96 (-62.41;-28.98)	19.65 (12.83;32.85)	-10.48 (-36.66;36.75)

Tunisia	119.07 (6.24;599.9)	213.12 (7.25;862.86)	78.98 (-51.17;445.75)	306.87 (12.35;1091.2)	157.73 (-20.17;2157.57)
Turkey	106.57 (90.68;124.98)	202.02 (156.71;247.28)	89.57 (48;129.77)	256.96 (188.85;345.58)	141.12 (81.21;219.3)
United Arab Emirates	106.88 (10.81;460.05)	248.35 (14.12;1141.37)	132.36 (-26.18;695.54)	516.24 (22.59;2281.88)	383.02 (5.06;2345.23)
Yemen	264.99 (12.28;1238.03)	468.31 (7.49;2327.78)	76.73 (-68.85;602.56)	772.91 (12.7;3178.84)	191.68 (-57.02;3294.63)
**South Asia**					
Bangladesh	379.3 (3.69;1937.21)	673.57 (6.43;3343.53)	77.58 (-61.62;899.2)	841.97 (8.05;3979.09)	121.98 (-64.62;2506.01)
Bhutan	45.13 (0.75;225.87)	93.42 (3.09;376.51)	107.02 (-7.34;1108.78)	126.59 (4.58;455.15)	180.53 (0.37;3522.34)
India	132108.58 (125968.23;139057.07)	35653.95 (29851.83;41591.05)	-73.01 (-77.15;-68.64)	31642.47 (21845.06;48219.55)	-76.05 (-83.39;-63.94)
Nepal	3206.58 (34.32;16948.31)	2485.86 (10.31;13354.02)	-22.48 (-96.58;51.37)	2842.62 (7.54;16556.76)	-11.35 (-97.68;80.72)
Pakistan	1819.14 (23.83;9670.32)	7006.48 (127.66;29968.47)	285.15 (22.28;3825.63)	11332.35 (247.67;41701.03)	522.95 (73.5;12410.29)
**Southeast Asia, East Asia, and Oceania**					
**East Asia**					
China	19833.77 (19344.05;20455.45)	32673.47 (28616.16;38444.21)	64.74 (43.58;95.06)	34126 (23910.17;44773.58)	72.06 (19.7;125.38)
North Korea	477.8 (7.7;2989.05)	886.68 (8.78;5288.9)	85.58 (-65.03;396.23)	1029.3 (7.17;5821.79)	115.42 (-73.29;1755.87)
Taiwan (Province of China)	175.11 (165.51;185.29)	150.05 (112.91;205.72)	-14.31 (-36.47;17.67)	80.45 (34.24;144.5)	-54.06 (-80.34;-17.4)
**Oceania**					
American Samoa	0.32 (0.27;0.4)	0.4 (0.31;0.51)	25.04 (2.92;53.68)	0.69 (0.47;0.97)	111.7 (50.61;201.42)
Federated States of Micronesia	12.62 (0.19;65.34)	49.32 (1.84;174.17)	290.79 (66.83;6795.74)	92.24 (2.83;337.73)	630.97 (142.63;21891.84)
Fiji	12.27 (9.77;15.42)	12.82 (8.73;21.01)	4.47 (-18.38;42.84)	16.82 (11.08;25.61)	37.07 (4.18;85.32)
Guam	3.96 (3.33;4.8)	4.94 (3.8;6.29)	24.7 (1.4;47.83)	7.3 (4.97;10.48)	84.19 (28.62;161.56)
Kiribati	0.56 (0.48;0.68)	0.53 (0.43;0.67)	-5.03 (-14.64;10.27)	0.7 (0.47;1.17)	25.43 (-10.69;103.05)
Marshall Islands	1.8 (0.03;12.13)	3.79 (0.17;18.88)	110.31 (-13.94;2920.32)	7.33 (0.35;33.49)	306.02 (21.92;11857.98)
Northern Mariana Islands	0.49 (0.41;0.59)	0.63 (0.47;0.81)	26.93 (2.57;56.05)	1.43 (0.9;2.15)	190.53 (87.89;342.33)
Papua New Guinea	2450.82 (1067.96;5509.02)	1302.09 (487.84;4103.79)	-46.87 (-71.92;-16.7)	1484.72 (367.5;5082.24)	-39.42 (-78.92;10.57)
Samoa	6.36 (0.12;42.55)	12.66 (0.55;59.24)	99.07 (-22.13;2825.68)	21.09 (0.86;101.98)	231.64 (-7.73;9388.74)
Solomon Islands	18.59 (0.35;123.76)	39.79 (1.78;208.53)	114.06 (-9.94;3598.32)	73.78 (3.09;347.12)	296.91 (17.65;13592.08)
Tonga	1.12 (0.88;1.43)	1.56 (1.2;2)	39.53 (14.06;72.53)	2.75 (1.87;3.88)	146.44 (73.43;254.31)
Vanuatu	9.21 (0.18;55.3)	18.9 (0.95;88.43)	105.27 (-10.73;2657.98)	35.5 (1.55;173.72)	285.44 (17.14;10401.83)
**Southeast Asia**					
Cambodia	3028.23 (2057.95;4101.98)	1036.19 (486.9;1753.19)	-65.78 (-79.98;-47.65)	614.69 (142.78;1472.76)	-79.7 (-94.14;-57.02)
Indonesia	4088.61 (3775.54;4521.61)	6726.54 (5851.13;7917.85)	64.52 (49.05;89.23)	8949.7 (7670.32;12139.88)	118.89 (94.9;205.31)
Laos	477.66 (2.41;3670.72)	556.85 (3.9;4706.72)	16.58 (-72.67;669.44)	717.57 (2.69;5620.34)	50.23 (-82.91;1630.87)
Malaysia	2072.91 (1765.39;2377.55)	1298.77 (744.87;1869.73)	-37.35 (-59.32;-12.45)	1528.1 (803.26;2352)	-26.28 (-55.54;11.96)
Maldives	0.54 (0.49;0.6)	0.74 (0.64;0.84)	36.87 (17.85;60.67)	0.91 (0.79;1.05)	68.78 (43.38;99.7)
Mauritius	69.2 (65.19;73.41)	87.14 (72.84;102.03)	25.92 (5.06;48.43)	100.89 (75.68;128.05)	45.79 (9.71;85.76)
Myanmar	15513.05 (12886.16;18817.17)	4321.09 (3031.61;5793.64)	-72.15 (-78.82;-63.49)	4026.19 (2375.84;5848.41)	-74.05 (-84.03;-62.48)
Philippines	4298.61 (4223.01;4372.08)	4847.1 (4279.91;5651.72)	12.76 (-0.93;31.74)	8647.68 (6134.25;12564.85)	101.17 (42.86;191.32)
Sri Lanka	74.01 (63.65;87.34)	54.67 (30.8;84.81)	-26.14 (-55.53;13.62)	84.64 (41.93;143.44)	14.37 (-41.57;92.75)
Seychelles	5.15 (4.94;5.52)	5.88 (5.24;6.74)	14.13 (4.81;24.03)	6.2 (5.4;7.43)	20.46 (7.65;40.17)
Thailand	16920.36 (13432.04;21890.47)	15403.95 (10403.95;24136.35)	-8.96 (-39.68;18.9)	12630.2 (6914.17;22639)	-25.36 (-59.33;10.47)
Timor-Leste	216.89 (4.93;1270.32)	229.2 (0.55;1316.87)	5.68 (-95.44;128.3)	272.94 (0;1506.96)	25.85 (-100;298.65)
Vietnam	10127 (8797.79;12003.53)	10474.28 (8004.58;14463.24)	3.43 (-11.32;22.79)	10706.14 (7984.95;15197.33)	5.72 (-13.38;31.13)
**Sub-Saharan Africa**					
**Central sub-Saharan Africa**					
Angola	12971.63 (9770.16;16930.73)	15148.26 (10567.52;20446.23)	16.78 (-6.07;40.49)	15796.87 (5302.74;27404.73)	21.78 (-53.96;97.58)

Central African Republic	10548.72 (8695.9;12642.72)	4716.76 (3636.21;6244.11)	-55.29 (-63.52;-43.75)	3820.82 (1796.96;7079.78)	-63.78 (-82.58;-32.62)
Congo (Brazzaville)	5618.37 (4531.7;6861.83)	5309.34 (4048.25;6752.54)	-5.5 (-17.55;4.68)	5445.94 (3538.07;7613.83)	-3.07 (-28.55;18.33)
DR Congo	42001.78 (34533.83;50396.39)	13539.61 (9287.5;18613.25)	-67.76 (-75.6;-58.54)	11497.4 (4732.09;20322.97)	-72.63 (-88.53;-56.39)
Equatorial Guinea	1639.21 (1253.66;2138.24)	1576.78 (891.63;2461.16)	-3.81 (-38.67;39.58)	1350.7 (249.85;2992.31)	-17.6 (-82.6;77.95)
Gabon	2333.85 (1783.28;3051.4)	494.35 (210.77;867.59)	-78.82*** (-90.38;-66.71)	318.02 (69.7;769.49)	-86.37 (-96.77;-70.82)
**Eastern sub-Saharan Africa**					
Burundi	8821.18 (7850.09;9945.39)	2062.27 (1451.15;2909.91)	-76.62*** (-82.99;-68.25)	1797.43 (865.89;3164.6)	-79.62 (-89.75;-64.51)
Comoros	1.17 (0.02;8.39)	2.59 (0.07;15.48)	121.45 (-46.33;10326.88)	6.17 (0.13;41.3)	426.48 (-6.4;51542.73)
Djibouti	967.9 (749.96;1256.44)	671.76 (446.94;975.26)	-30.6 (-45.7;-15.76)	659.92 (272.46;1154.34)	-31.82 (-69.2;7.41)
Eritrea	2820.88 (2337.79;3381.45)	1492.29 (1082.76;1997.1)	-47.1 (-57.24;-35.79)	1313.57 (651.92;2149.51)	-53.43 (-76.08;-25.19)
Ethiopia	49414.68 (43852.47;55575.28)	7484.62 (3927.29;11856.23)	-84.85*** (-91.44;-76.99)	5015.9 (1784.04;10007.73)	-89.85*** (-96.12;-80.67)
Kenya	78721.66 (74421.7;83426.91)	46376.49 (40006.99;53966.39)	-41.09 (-47.85;-32.82)	42381.52 (32364.73;54653.73)	-46.16 (-58.38;-31.14)
Madagascar	2825.61 (2264.83;3501.24)	3230.13 (1768.92;8112.34)	14.32 (-32.52;203.76)	7713.62 (1678.52;34620.14)	172.99 (-37.78;1187.32)
Malawi	60026.65 (51683.94;71264.73)	23763.48 (19526.01;28794.55)	-60.41 (-66.19;-53.05)	16367.48 (10057.17;23545.17)	-72.73 (-81.66;-62.62)
Mozambique	89990.75 (74176.39;110239.25)	53570.23 (36702.6;73889.16)	-40.47 (-54.45;-27.66)	47322.45 (23377.46;76986.64)	-47.41 (-71.42;-24.89)
Rwanda	6952.76 (5951.1;8260.49)	3226 (2517.95;4077.39)	-53.6 (-61.58;-46.03)	2339.35 (1405.91;3428.69)	-66.35 (-78.26;-54.31)
Somalia	3254.45 (1275.66;9110.74)	2225.93 (331.7;7692.32)	-31.6 (-78.56;-7.32)	2632.87 (100.44;9919.79)	-19.1 (-93.77;28.17)
South Sudan	6187.44 (2289.23;15862.05)	3820.75 (1088.57;12517.45)	-38.25 (-67.9;-18.38)	4071.87 (82.7;15834.99)	-34.19 (-97.78;5.89)
Tanzania	91291.33 (72450.12;112245.55)	26566.25 (17724.15;38167.17)	-70.9 (-79.93;-58.32)	23117.07 (7720.98;49368.01)	-74.68 (-90.91;-43.34)
Uganda	67453.37 (56794.76;80714.09)	27127.72 (21147.49;37683.1)	-59.78 (-67.74;-44.7)	21526.34 (11567.59;46200.3)	-68.09 (-81.99;-32.41)
Zambia	43419.7 (36440.72;53109.12)	10102.75 (5265.47;17140.14)	-76.73*** (-87.32;-65.49)	7191.88 (3430.48;14789.54)	-83.44 (-91.62;-70.29)
**Southern sub-Saharan Africa**					
Botswana	9667.53 (7899.53;12157)	3393.32 (1957.04;5175.81)	-64.9 (-78.33;-51.87)	1916.2 (681.24;4050.79)	-80.18 (-92.82;-61.48)
Lesotho	12118.12 (9978.01;15252.06)	5380.44 (4050.54;7007.08)	-55.6 (-63.94;-48.59)	4282.37 (2893.17;5980.37)	-64.66 (-75.18;-53.49)
Namibia	8148.77 (6653.24;10121.39)	3171.36 (1883.28;4735.1)	-61.08 (-74.38;-49.33)	2326.97 (835.96;4306.82)	-71.44 (-88.59;-54.28)
South Africa	293922.36 (264332.99;330383.16)	88312.74 (62791.25;114544.86)	-69.95 (-77.37;-62.84)	52735.24 (26265.27;85378.82)	-82.06 (-90.88;-72.32)
eSwatini	8543.85 (6892.66;10751.97)	1403.69 (940.56;1895.37)	-83.57*** (-88.25;-77.94)	575.64 (247.54;1099.25)	-93.26*** (-96.96;-86.72)
Zimbabwe	67092.11 (53252.06;83154.01)	17071.48 (11660.85;23818.12)	-74.56*** (-82.44;-63.94)	15494.74 (5203.54;31262.08)	-76.91 (-92.31;-52.05)
**Western sub-Saharan Africa**					
Benin	2712.5 (2209.95;3361.69)	1806.71 (1267.18;2493.97)	-33.39 (-47.35;-20.09)	1654.73 (885.4;2603.62)	-39 (-63.55;-17.17)
Burkina Faso	6146.42 (4903.1;7472.46)	2255.55 (1526.75;3184.93)	-63.3 (-72.26;-53.33)	1639.97 (554.19;3064.03)	-73.32 (-89.76;-53.75)
Cameroon	33180.22 (27123.71;40520.18)	21905 (16313.05;28763.77)	-33.98 (-45.37;-23.4)	18369.74 (9437.63;29016.55)	-44.64 (-69.13;-21.34)
Cape Verde	166.76 (120.11;234.55)	123.43 (76.88;190.88)	-25.98 (-42.34;-8.82)	141.75 (73.49;230.31)	-14.99 (-45.16;15.72)
Chad	7336.77 (5522.07;9886.09)	5802.05 (4145.59;7987.97)	-20.92 (-37.36;-4.34)	5952.09 (2501.58;9724.67)	-18.87 (-60.45;20.98)
Cote d'Ivoire	33451.95 (24462.06;43189.47)	19952.42 (12762.71;30209.57)	-40.35 (-56.91;-15.56)	25532.28 (8743.7;46256.83)	-23.67 (-71.37;34.26)
The Gambia	1109.14 (824.08;1487.14)	858.59 (557.09;1235.65)	-22.59 (-40.11;-5.82)	824.31 (263.89;1470.76)	-25.68 (-71.15;14.5)
Ghana	20982.77 (17201.41;25642.49)	13336.6 (10110.48;17617.26)	-36.44 (-45.9;-27.39)	12860.17 (7165.02;19907.51)	-38.71 (-63.87;-12.05)
Guinea	5036.82 (4041.03;6333.64)	2141.62 (1286.72;3203.64)	-57.48 (-71.06;-45.55)	1988.53 (798.3;3435.94)	-60.52 (-82.54;-39.58)
Guinea-Bissau	2006.71 (1477.28;2680.29)	1266.4 (796.12;1873.95)	-36.89 (-51.38;-22.73)	1251.47 (541.77;2123.31)	-37.64 (-69.4;-8.96)
Liberia	2772.26 (2253.74;3316.17)	1712.37 (1291.21;2248.4)	-38.23 (-50.34;-23.93)	1779.02 (957.41;2906.11)	-35.83 (-63.15;0.09)
Mali	5907.6 (4828.65;7319.49)	4919.43 (3467.24;6651.79)	-16.73 (-33.98;-1.65)	4939.93 (2742.77;7510.91)	-16.38 (-48.36;10.31)
Mauritania	20.06 (0.56;151.36)	23.78 (0.75;200.02)	18.57 (-56.99;761.1)	35.13 (0.88;277.7)	75.15 (-53.17;1455.08)
Niger	3991.63 (3401.89;4760.52)	1812.17 (1425.18;2262.03)	-54.6 (-61.62;-47.29)	1647.81 (998.25;2457.06)	-58.72 (-73.7;-43.3)
Nigeria	210794.59 (171525.54;256867.14)	150944.81 (112848.96;192956.1)	-28.39 (-39.77;-19.76)	147417.36 (88878.48;211887.41)	-30.07 (-53.96;-11.02)
Sao Tome and Principe	0.31 (0.18;0.53)	0.2 (0.08;0.37)	-34.67 (-65;3.94)	0.27 (0.08;0.55)	-12.73 (-66.67;78.26)

Senegal	2785.84<br>(2266.3;3436.11)	1803.71<br>(1365.43;2424.43)	-35.25<br>(-45.15;-25.33)	2023.39<br>(1319.94;2957.85)	-27.37<br>(-47.58;-3.88)
Sierra Leone	3246.43<br>(2446.17;4213.92)	2043.77<br>(1375.77;2871.05)	-37.05<br>(-54.79;-17.95)	1860.26<br>(621.26;3716.91)	-42.7<br>(-79.03;8.91)
Togo	9226.31<br>(7501.72;11582.09)	3173.5<br>(2154.62;4354.26)	-65.6<br>(-73.56;-57.66)	2338.99<br>(921.33;4152.6)	-74.65<br>(-89.35;-58.23)

***Meets Target
UI = Uncertainty Interval

Appendix Table 3: Forecast number of incident cases and percent change in incident cases between 2010-2020 and 2010-2030

	2010 (95% UI)	2020 (95% UI)		2030 (95% UI)	
	Number of cases	Number of cases	Percent Change	Number of cases	Percent Change
**Central Europe, Eastern Europe, and Central Asia**					
**Central Asia**					
Armenia	113.49 (82.51;155.71)	176.64 (128.56;259.74)	55.64 (12.25;134.63)	188.28 (142.78;268.53)	65.9 (20.45;148.78)
Azerbaijan	173.68 (109.11;228.55)	353.64 (233.64;527.2)	103.61 (45.88;185.5)	339.56 (223.71;501.43)	95.51 (41.56;213.61)
Georgia	552.86 (369.63;838.69)	426.16 (277.47;710.14)	-22.92 (-37.51;-5.25)	475.39 (311.5;793.05)	-14.01 (-29.45;4.75)
Kazakhstan	851.37 (719.84;1030.11)	2555.89 (1726.17;3309.45)	200.21 (111.51;273.49)	2408.92 (1659.17;3051.95)	182.95 (101.13;248.84)
Kyrgyzstan	510.82 (351.63;743.61)	936.76 (596.86;1455.91)	83.38 (17.39;179.85)	1074.5 (700.17;1632.68)	110.35 (34.86;225.95)
Mongolia	44 (15.71;91.43)	70.85 (17.56;156.16)	61.02 (-9.79;153.28)	97.59 (26.25;204.03)	121.79 (33.74;240.89)
Tajikistan	271.05 (195.07;332.97)	674.77 (364.81;952.82)	148.95 (71.69;242.25)	829.2 (492.18;1191.42)	205.92 (115.11;327.53)
Turkmenistan	108.73 (92.44;139.36)	177.19 (135.26;259.74)	62.97 (35.2;103.63)	259.56 (197.91;385.65)	138.73 (97.66;199.54)
Uzbekistan	796.65 (113.59;1302.66)	2398.88 (734.08;4032.67)	201.12 (27.6;2405)	2740.39 (1022.03;4485.46)	243.99 (67.85;2698.03)
**Central Europe**					
Albania	2.4 (1.94;3.19)	2.88 (2.23;4.01)	20.15 (8.01;35.08)	2.68 (2.15;3.63)	11.6 (-2.76;27.99)
Bosnia and Herzegovina	3.25 (2.64;4.07)	3.56 (2.62;4.69)	9.55 (-5.28;24.4)	3.97 (3.01;4.99)	22.06 (6.92;37.72)
Bulgaria	98.53 (88.4;112.53)	125.61 (95.99;158.81)	27.48 (-4.29;62.52)	125.13 (96.78;182.28)	26.99 (-4.99;91.58)
Croatia	45.91 (32.69;62.05)	46.08 (26.43;62.32)	0.37 (-35.41;45.18)	44.79 (27.94;60.06)	-2.43 (-29.92;38.7)
Czech Republic	109.4 (72.54;155.57)	111.87 (52.34;174.38)	2.25 (-37.93;21.32)	111.26 (59.97;166.03)	1.7 (-25.2;17.89)
Hungary	89.42 (68.77;116.9)	140.05 (94.93;211.97)	56.62 (29.51;94.56)	167.72 (113.62;256.51)	87.57 (56.55;134.19)
Macedonia	8.31 (6.19;10.16)	9.41 (7.14;12.06)	13.33 (-3.76;29.43)	9.22 (6.87;11.59)	11 (-0.61;23.73)
Montenegro	5.87 (4.49;7.16)	6.56 (5.03;8.38)	11.69 (-10.86;27.96)	6.37 (4.91;8.12)	8.47 (-12.24;23.12)
Poland	681.86 (401.99;1151.95)	674.35 (348.44;1179.13)	-1.1 (-28.22;14.14)	641.19 (389.25;1067.54)	-5.96 (-17.55;13.75)
Romania	1199.06 (967.29;1432.68)	941.63 (704.03;1167.66)	-21.47 (-35.05;-3.86)	1001.52 (798.12;1205.34)	-16.47 (-27.68;-2.22)
Serbia	173.83 (123.61;251.5)	140.99 (91.71;227.43)	-18.89 (-36.29;1.87)	171.91 (110.6;276.78)	-1.1 (-23.49;25.4)
Slovakia	24.91 (18.9;33.02)	23.28 (16.98;32.28)	-6.55 (-16.79;9.79)	22.45 (16.92;29.89)	-9.89 (-17.35;5.64)
Slovenia	11.6 (8.11;16.88)	12.39 (8.62;18.5)	6.79 (-8.64;24.48)	12.97 (9.63;19.51)	11.79 (-4.54;43.74)
**Eastern Europe**					
Belarus	1024.89 (874.35;1259.12)	2505.67 (1695.72;3519.11)	144.48 (69.22;233.9)	2114.07 (1452.22;2894.38)	106.27 (45.62;173.97)
Estonia	275.16 (229.92;329.47)	208.19 (158.36;272.5)	-24.34 (-44.27;10.18)	207.36 (174.69;250.13)	-24.64 (-38.86;-1.83)
Latvia	278.68 (247.01;331.43)	436.09 (328.01;508.82)	56.49 (5.47;85.71)	351.66 (261.59;404.65)	26.19 (-16.49;51.04)
Lithuania	162.1 (120.25;203.38)	149.18 (45.76;217.73)	-7.97 (-66.05;11.83)	138.28 (51.17;193.19)	-14.7 (-62.41;2.83)
Moldova	624.31 (440.25;880.39)	786.49 (570.85;1103.18)	25.98 (5.39;49.75)	765.08 (568.94;1050.1)	22.55 (-0.08;49.87)
Russian Federation	61827.71 (48996.61;77237.63)	141267.28 (115071.78;173465.44)	128.49 (76.19;194.9)	118615.82 (98296.54;140999.41)	91.85 (47.98;158.47)
Ukraine	8145.07 (5448.83;9714.34)	27454.77 (18396.96;34764.1)	237.07 (154.06;375.66)	22940.9 (14730.74;28842.96)	181.65 (123.85;281.99)
**High-income**					
**Australasia**					
Australia	1025 (635.46;1554.31)	1508.62 (887.93;2197.94)	47.18 (30.01;75)	1810.93 (1102.65;2618.36)	76.68 (54.95;110.03)
New Zealand	190.59 (101.36;295.29)	208.91 (101.96;332.05)	9.61 (-25.92;49.52)	276.32 (135.98;431.21)	44.98 (0.66;97.16)
**High-income Asia-Pacific**					
Brunei	24.59 (14.49;38.94)	36.33 (20.5;58.13)	47.73 (19.77;68.49)	38.24 (22.74;60.03)	55.51 (28.03;80)

Japan	1527.03 (1059.1;2130.27)	1391.76 (745.78;2022.49)	-8.86 (-35.64;4.19)	1328.38 (778.78;1929.35)	-13.01 (-32.64;-2.21)
South Korea	1397.69 (678.13;2610.27)	1425.31 (336.18;2773.11)	1.98 (-73.59;78.21)	1763.43 (413.9;3374.91)	26.17 (-67.04;120.85)
Singapore	144.09 (74.63;237.03)	198.44 (106.19;308.08)	37.71 (16.28;75.05)	277.61 (153.84;428.95)	92.66 (59.25;154)
**High-income North America**					
Canada	2096 (1174.39;3210)	4028.32 (1725.36;6624.51)	92.19 (23.62;144.52)	5317.77 (2293.94;8645.27)	153.71 (64.25;216.07)
Greenland	7.58 (4.39;12.23)	11.63 (4.94;19.01)	53.33 (-6.33;80.63)	14.01 (6.28;22.56)	84.73 (19.48;122.2)
USA	39513 (24086.87;60204.76)	57180.66 (21291.95;91742.03)	44.71 (-22.9;73.1)	73931.07 (28330.51;119130.85)	87.11 (2.54;122.59)
**Southern Latin America**					
Argentina	10712.74 (5002.14;18382.79)	12821.53 (6612.22;21877.64)	19.68 (6.05;36.57)	13501.18 (7439.69;22629.67)	26.03 (11.21;50.66)
Chile	2963.62 (1632.35;4925.14)	4107.26 (2167.43;6742.56)	38.59 (27.62;49.54)	4145.51 (2275.14;6627.52)	39.88 (31.7;49.83)
Uruguay	388.47 (280.05;543.68)	869.88 (669.81;1126.82)	123.92 (71.24;204.79)	921.13 (700;1299.05)	137.11 (72.82;245.43)
**Western Europe**					
Andorra	4.64 (0.26;20.9)	4.76 (0.27;22.12)	2.73 (-31.62;59.46)	4.69 (0.28;21.96)	1.21 (-30.31;61.56)
Austria	650.8 (333.84;1040.52)	739.69 (404.68;1130.52)	13.66 (-9.06;65.49)	778.45 (422.66;1294.12)	19.61 (-5.48;83.81)
Belgium	997 (457.82;1742.86)	1210.57 (486.89;1942.97)	21.42 (-12.87;74.83)	1329.63 (589.41;2128.76)	33.36 (-3.29;93.52)
Cyprus	80 (45.35;126.25)	116.82 (83.36;165.79)	46.03 (18.36;97)	124.11 (90.09;174.39)	55.14 (26.71;111.81)
Denmark	386.37 (243.09;565.64)	433.62 (221.9;668.75)	12.23 (-31.71;49.89)	474.06 (251.88;716.19)	22.69 (-23.47;64.07)
Finland	174 (81.05;300.62)	160.88 (45.36;276.74)	-7.54 (-59.62;55.18)	164.35 (52.84;273.35)	-5.55 (-52.61;54.75)
France	3921 (2142.49;6420.98)	5536.48 (2803.48;9167.1)	41.2 (16.5;73.36)	6571.04 (3378.8;11101.29)	67.59 (35.38;105.37)
Germany	5915.97 (3040.44;9154.08)	7168.96 (4234.6;10267.86)	21.18 (-9.81;87.61)	8503.65 (5026.21;11900.9)	43.74 (9.36;117.96)
Greece	691 (497.12;1065.77)	513.76 (330.16;842.92)	-25.65 (-36.65;-10.77)	501 (331.39;788.76)	-27.5 (-36.49;-16.37)
Iceland	12 (6.7;17.83)	20.56 (9.87;30.79)	71.34 (7.91;139.61)	21 (11.04;31.39)	75.02 (28.56;146.96)
Ireland	486 (229.81;774.48)	548.69 (215.13;851.89)	12.9 (-24.4;44.04)	622.92 (278.27;953.13)	28.17 (-4.31;69.44)
Israel	598.97 (313.78;968.61)	734.37 (396.36;1038.34)	22.61 (-9.85;86.46)	889.53 (502.3;1273.38)	48.51 (15.74;119.15)
Italy	5523.25 (3083.72;8186.98)	7893.24 (4724.57;11974.53)	42.91 (9.25;102.73)	9151.79 (5758.04;13564.01)	65.7 (26.71;137.24)
Luxembourg	57 (27.96;90.84)	99.73 (45.07;146.62)	74.96 (22.65;144.05)	109.72 (53.86;160.53)	92.5 (39.64;165.46)
Malta	67.64 (38.13;103.86)	97.21 (52.37;152.74)	43.71 (31.57;56.51)	89.38 (48.95;138.62)	32.13 (22.25;42.59)
Netherlands	947.11 (606.65;1266.42)	583.64 (292.45;880.96)	-38.38 (-65.9;-0.85)	688.56 (346.4;1036.35)	-27.3 (-61.21;17.65)
Norway	226.95 (119.56;356.83)	301.36 (163.98;451.38)	32.79 (5.31;76.06)	335.61 (182.07;500.99)	47.88 (17.24;95.9)
Portugal	5927 (3216.4;8508.34)	6762.85 (3460.81;9899.57)	14.1 (-10.78;45.24)	5151.88 (2584.39;7483.35)	-13.08 (-32.41;14.24)
Spain	3694.66 (1845.46;5966.82)	6738.49 (2884.48;12444.53)	82.38 (48.88;119.08)	7036.14 (3077.14;12741.8)	90.44 (52.13;130.62)
Sweden	447 (249.04;681.65)	660.99 (313.15;1013.58)	47.87 (4.34;76.31)	662.59 (333.31;1002.99)	48.23 (8.55;73.15)
Switzerland	588.35 (294.18;866.98)	733.29 (253.63;1126.57)	24.63 (-15.46;47.47)	936.43 (312.18;1416.55)	59.16 (8.07;91.74)
United Kingdom	6088 (3450.51;8884.71)	7316.94 (4307.7;10374.73)	20.19 (4.66;37.61)	7891.02 (4774.19;11149.16)	29.62 (13.89;49.37)
**Latin America and Caribbean**					
**Andean Latin America**					
Bolivia	2263.76 (261.96;8692.58)	2847.05 (328.51;11144.45)	25.77 (-21.28;76.69)	3585.21 (476.53;13165.67)	58.37 (11.94;134.79)
Ecuador	2493.67 (1997.24;3344.43)	3349.02 (2709.91;4275.23)	34.3 (16.45;51.91)	3779.36 (3190.54;4660.74)	51.56 (28.65;75)
Peru	6342.4 (5028.63;8378.15)	7504.36 (5235.31;11576.45)	18.32 (-5.52;52.81)	8778.81 (6541.15;12493.24)	38.41 (19.26;67.59)
**Caribbean**					
Antigua and Barbuda	8.4 (7.51;10.4)	9.98 (8.35;13.08)	18.8 (2.85;40.92)	13.87 (11.55;18.15)	64.97 (41.86;96.78)
The Bahamas	236.72 (216.79;267.05)	363.86 (306.14;469.52)	53.71 (27.65;100.72)	333.73 (270.55;438.36)	40.98 (11.14;90.8)
Barbados	64.97 (54.96;84.57)	67.95 (47.89;93.46)	4.59 (-23.07;36.83)	56.03 (39.66;74.82)	-13.76 (-35.39;12.34)
Belize	151.61 (136.6;172.4)	273.53 (228.4;337.27)	80.41 (57.66;110.33)	286.06 (248.1;342.69)	88.67 (66.22;113.87)
Bermuda	6.58 (5.78;7.26)	6.61 (5.84;7.57)	0.58 (-12;18.68)	10 (8.78;11.62)	52 (31.34;83.98)

Cuba	2252.53 (1415.25;3200.19)	2820.47 (1569.55;4377.65)	25.21 (3.68;49.51)	2680.86 (1495.28;4178.78)	19.02 (-0.32;44.09)
Dominica	7.06 (5.89;9.72)	7.23 (5.72;10.31)	2.47 (-9.96;19.69)	9.91 (7.87;13.75)	40.4 (22.71;63.89)
Dominican Republic	4469.58 (2861.94;6256.74)	4212.9 (2641.88;6341.49)	-5.74 (-25.94;24.55)	4002.33 (2462.15;6055.52)	-10.45 (-30.2;17.61)
Grenada	7.98 (7.12;9.58)	7.25 (6.18;9.16)	-9.1 (-21.1;6.76)	10.78 (9.19;13.37)	35.1 (16.79;59.13)
Guyana	811.03 (547.04;1034.93)	634.78 (458.09;798.33)	-21.73 (-33.83;-4.61)	655.27 (422.28;871.73)	-19.21 (-33.5;-1.59)
Haiti	11026.56 (8897.46;13756.17)	9247.65 (6862.17;12115.12)	-16.13 (-30.21;0.96)	9248.96 (6674.68;12383.75)	-16.12 (-31.44;0.99)
Jamaica	1152.7 (977;1432.04)	1275.36 (969.07;1680.21)	10.64 (-8.49;31.47)	1180.97 (925.82;1498.16)	2.45 (-14.69;24.02)
Puerto Rico	160.27 (135.12;183.92)	212.03 (178.27;247.36)	32.29 (15;53.4)	275.58 (232.34;322.94)	71.95 (49.46;101.86)
Saint Lucia	8.46 (7.41;11.09)	9.77 (8.12;13.15)	15.55 (0.24;33.48)	13.34 (11.06;17.98)	57.82 (35.34;83.26)
Saint Vincent and the Grenadines	22.8 (20.02;27.73)	22.89 (19.2;29.81)	0.41 (-13.49;19.25)	30.4 (25.36;39.72)	33.32 (14.01;58.66)
Suriname	203.59 (182.24;237.1)	217.53 (175.61;290.27)	6.85 (-12.8;33.97)	199.65 (159.19;254.83)	-1.93 (-19.93;22.02)
Trinidad and Tobago	529.87 (473.84;629.72)	409.76 (345.57;501.76)	-22.67 (-33.1;-10.42)	346.99 (282.18;425.07)	-34.51 (-45.9;-22.22)
Virgin Islands	12.75 (10.34;17.11)	16 (10.89;24.81)	25.48 (0.94;53.37)	19.51 (13.67;29.71)	53.03 (24.39;91.75)
**Central Latin America**					
Colombia	5887.09 (4956.83;7386)	9959.03 (7459.92;13317.47)	69.17 (45.72;99.95)	10976.35 (8394.65;14526.39)	86.45 (58.37;125.71)
Costa Rica	412.93 (340.47;561.65)	413.6 (310.02;563.29)	0.16 (-13.61;16.8)	463.28 (387.82;591.04)	12.19 (-0.7;31.33)
El Salvador	1934.24 (1243.9;2453.07)	1368.53 (847.96;1846.69)	-29.25 (-45.67;-8.54)	1526.76 (959.75;2013.45)	-21.07 (-37.3;-3.46)
Guatemala	1311.78 (756;2592.44)	2781.23 (1356.3;6026.18)	112.02 (35.99;219.77)	3909.21 (1974.41;7793.03)	198.01 (97.07;343.67)
Honduras	498.16 (407.87;620.78)	611.31 (422.4;835.82)	22.71 (-14.57;68.11)	770.52 (555.39;1001.37)	54.67 (11.56;98.77)
Mexico	15296.33 (12046.47;23687.19)	21821.41 (16256.39;28849.67)	42.66 (-6.69;70.41)	26813.32 (20223.97;34485.54)	75.29 (16.09;106.42)
Nicaragua	1404.98 (1096.39;1720.97)	2118.35 (1428.04;2890.07)	50.77 (17.45;95.51)	2400.1 (1635.24;3304.34)	70.83 (32.68;123.52)
Panama	1158.15 (935.51;1538.29)	1881.37 (1459.4;2534.73)	62.45 (46;80.65)	1691.21 (1325.4;2296.4)	46.03 (30.58;64.46)
Venezuela	5657.73 (4840.04;7291.02)	8372.24 (6644.58;10357.87)	47.98 (17.61;79.14)	9563.36 (7685.36;11582.49)	69.03 (31.29;106.27)
**Tropical Latin America**					
Brazil	62884.89 (48144.5;74554.55)	89225.18 (65486.2;106959.24)	41.89 (24.24;53.34)	96956.73 (69943.28;116647.03)	54.18 (38.97;67.32)
Paraguay	1638.4 (1116.29;2092.46)	1842.06 (1221.57;2476.36)	12.43 (-3.81;31.33)	2157.2 (1420.89;2812.76)	31.67 (17.96;47.98)
**North Africa and Middle East**					
Afghanistan	322.06 (26.46;1251.17)	1051.66 (51.25;4338.87)	226.54 (32.13;568.59)	1301.33 (76.62;5447.9)	304.07 (100.07;717.45)
Algeria	1073.47 (109.92;3783.83)	1050.31 (24.06;4049.01)	-2.16 (-96.23;135.2)	1375.75 (0.82;5063.44)	28.16 (-99.89;217.75)
Bahrain	19.87 (17.9;23.03)	26.48 (22.41;31.72)	33.28 (15.26;52.98)	51.83 (43.29;63)	160.82 (123.6;203.47)
Egypt	312.4 (257.02;374.11)	613.12 (440.32;786.72)	96.26 (46.94;153.7)	779.16 (577.59;1005.64)	149.41 (86.32;222.72)
Iran	1478.67 (1171.21;1815.78)	3333.82 (2208.97;4445.28)	125.46 (55.63;193.62)	3942.44 (2609.36;5512.15)	166.62 (81.43;251.43)
Iraq	123.74 (67.31;212.29)	252.34 (131.05;485.26)	103.92 (44.34;235.94)	345.05 (184.39;647.86)	178.85 (102.31;370.02)
Jordan	40.02 (31.23;54.62)	61.89 (42.8;78.73)	54.67 (0.13;118.6)	75.76 (52.6;95.29)	89.32 (26.12;163.73)
Kuwait	10.73 (8.62;12.59)	19.68 (15.54;25.16)	83.36 (57.95;119.65)	30.42 (24.02;38.05)	183.44 (142.97;235.17)
Lebanon	128.71 (11.24;539.58)	275.23 (15.35;1190.1)	113.84 (2.06;354.91)	244.82 (13.77;1138.45)	90.21 (-1.21;390.88)
Libya	112.33 (9.56;465.02)	214.23 (12.95;946.04)	90.71 (-7.59;330.35)	259.15 (17.33;1092.18)	130.7 (34.76;468.38)
Morocco	1364.39 (95.3;5722.65)	1286.15 (72.93;5512.66)	-5.73 (-51.36;114.92)	1566.2 (91.73;6695.47)	14.79 (-38.41;157.35)
Palestine	14.03 (9.99;20.61)	22.06 (14.36;33.85)	57.24 (35.97;82.17)	31.09 (21.07;48.49)	121.64 (93.62;158.85)
Oman	174.87 (7.95;306.85)	712.74 (332.63;1286.79)	307.58 (144.92;8758.68)	858.65 (401.35;1496.25)	391.01 (204;11088.36)
Qatar	6.7 (5.86;8.11)	12.93 (10.27;16.58)	92.88 (64.32;133.6)	22.23 (17.07;29)	231.51 (173.83;311.79)
Saudi Arabia	513.79 (199.45;801.37)	798.12 (473.81;1495.44)	55.34 (14.33;200.72)	1145.34 (727.88;1919.16)	122.92 (69.53;296.54)
Sudan	9647.11 (7162.61;12706.27)	8597.95 (4670.08;15463.12)	-10.88 (-46.53;46.02)	12216.42 (6488.16;20319.41)	26.63 (-24.96;86.47)
Syria	59.18 (43.89;87.16)	49.18 (33.07;74.86)	-16.91 (-32.48;1.04)	75.73 (53.01;114.92)	27.96 (7.49;51.66)

Tunisia	323.79 (35.78;1050.02)	458.94 (51.78;1708.48)	41.74 (-24.87;145.05)	506.87 (59.83;1826.6)	56.54 (-1.99;176.64)
Turkey	392.53 (315.93;525.74)	505.02 (356.75;745.79)	28.66 (7.99;47.86)	521.65 (392.94;753.99)	32.89 (17.35;50.95)
United Arab Emirates	179.97 (10.41;958.7)	280.7 (13.19;1554.78)	55.97 (-19.08;327.14)	549.35 (26.71;3036.12)	205.24 (58.96;843.31)
Yemen	545.62 (39.01;2462.8)	1219.33 (53.74;5466.52)	123.48 (-16.25;356.97)	1656.2 (86.64;7359.81)	203.55 (34.64;552.73)
**South Asia**					
Bangladesh	1152.29 (30.5;4864.12)	1077.2 (25.34;4586.19)	-6.52 (-48.25;85.29)	1282.34 (33.09;5501.78)	11.29 (-29.6;93.97)
Bhutan	102.15 (2.72;423.2)	150.59 (4.03;666.51)	47.42 (-14.72;188.94)	178.6 (5.66;739.49)	74.83 (21.53;244.41)
India	98676.87 (69514.03;135948.95)	108884.4 (68211.14;162430.12)	10.34 (-22.63;53.61)	139460.66 (86709.36;204484.74)	41.33 (-0.67;94.27)
Nepal	3992.99 (109.17;17279.43)	2555.25 (65.06;11359.95)	-36.01 (-66.57;30.36)	3535.95 (92.67;15522.98)	-11.45 (-53.11;66.73)
Pakistan	8379.76 (251.14;33265.19)	16320.34 (496.93;68397.41)	94.76 (8.93;309.66)	19750.21 (661;79870.13)	135.69 (55.17;399.46)
**Southeast Asia, East Asia, and Oceania**					
**East Asia**					
China	55710.48 (38322.9;89620.9)	36680.43 (15280.46;62276.58)	-34.16 (-63.89;-22.78)	43842.62 (20542.82;70824.76)	-21.3 (-51.8;-11.36)
North Korea	1172.65 (14.8;6047.75)	1257.31 (10.03;7960.25)	7.22 (-68.08;104.51)	1459.56 (14.51;8651.35)	24.47 (-55.39;117.27)
Taiwan (Province of China)	395.23 (281.21;616.77)	215.69 (107.45;384.38)	-45.43 (-68.02;-33.06)	193.29 (94.92;323.66)	-51.09 (-71.48;-40.68)
**Oceania**					
American Samoa	0.46 (0.29;0.73)	0.79 (0.45;1.34)	71.2 (27.5;131.68)	1.33 (0.73;2.27)	186.3 (102.91;278.8)
Federated States of Micronesia	38.24 (0.45;209.26)	91.23 (0.79;535.26)	138.55 (-8.26;419.99)	131.42 (1.1;821.96)	243.65 (13.72;719.3)
Fiji	31.63 (25.72;40.48)	54.17 (41.64;72.7)	71.23 (49.97;96.78)	58.19 (43.93;77.58)	83.94 (54.23;138.32)
Guam	5.41 (3.42;8.37)	8.29 (4.61;13.93)	53.15 (15.49;103.05)	11.53 (6.42;19.66)	113.07 (59.23;184.63)
Kiribati	0.58 (0.45;0.81)	0.8 (0.47;1.52)	38.72 (-8.07;153.13)	1.16 (0.66;2.31)	100.81 (26.69;282.23)
Marshall Islands	3.83 (0.04;21.79)	8.9 (0.09;57.26)	132.26 (11.11;283.66)	11.79 (0.14;72.01)	207.56 (63.07;392.31)
Northern Mariana Islands	0.58 (0.37;0.88)	0.83 (0.49;1.3)	41.12 (9.45;98.86)	1.32 (0.83;2.07)	125.88 (59.75;244.32)
Papua New Guinea	2194.95 (174.41;6585.57)	3430.58 (314.36;13294.76)	56.29 (-41.05;188.37)	3712.12 (309.67;14642.23)	69.12 (-42.27;222.79)
Samoa	13.32 (0.17;75.08)	30.49 (0.35;195.22)	128.91 (3.71;294.23)	36.04 (0.46;220.69)	170.58 (33.03;357.55)
Solomon Islands	36.82 (0.46;209.96)	96.88 (1.09;629.12)	163.09 (19.26;337.52)	125.49 (1.57;776.02)	240.78 (69.61;456.18)
Tonga	1.91 (1.17;3.15)	3.1 (1.7;5.42)	62.15 (23.5;115.16)	4.45 (2.57;7.52)	132.94 (73.29;225.98)
Vanuatu	26.03 (0.32;137.06)	45.11 (0.51;297.35)	73.3 (-78.68;317.79)	59.14 (0.76;369.16)	127.2 (-67.55;445.58)
**Southeast Asia**					
Cambodia	2349.78 (630.25;4575.15)	1187.32 (260.83;2781.92)	-49.47 (-84.37;12.72)	1035.21 (130.61;2621.98)	-55.94 (-92.31;-0.25)
Indonesia	15084.07 (12145.96;20575.33)	19535.81 (15806.6;26250.83)	29.51 (16.95;48.15)	22148.29 (18205.19;28675.95)	46.83 (30.69;71.7)
Laos	985.32 (11.96;5459.98)	898.95 (9.27;5651.94)	-8.77 (-55.71;49.53)	1221.31 (14.93;6988.14)	23.95 (-32.64;91.3)
Malaysia	3513.89 (2816.72;4053.88)	4992.32 (3411.44;6083.03)	42.07 (12.22;65.26)	6583.03 (4582.76;7956.46)	87.34 (51.37;118.84)
Maldives	1.01 (0.8;1.38)	1.41 (1.09;1.93)	39.25 (22.62;58)	2.04 (1.66;2.57)	101.64 (77.11;132.75)
Mauritius	313.6 (227.45;363.52)	333.7 (259.56;426.09)	6.41 (-22.22;50.65)	407.01 (326.67;506.72)	29.79 (-0.56;83.15)
Myanmar	14626.51 (10635.77;19818.62)	12062.13 (9131.89;16338.63)	-17.53 (-26.4;-6.47)	16921.46 (12804.54;23621.32)	15.69 (2.07;32.11)
Philippines	10538.92 (7142.01;16894.3)	16056.99 (9575.62;26171.67)	52.36 (20.01;85.41)	21440.83 (13414.79;35156.07)	103.44 (61.98;154.74)
Sri Lanka	141.98 (109.27;174.98)	327.44 (195.86;476.92)	130.63 (31.21;246.27)	406.01 (257.48;552.99)	185.96 (75.06;313.81)
Seychelles	6.79 (5.78;8.86)	6.39 (4.94;8.88)	-5.99 (-16.13;5.54)	8.84 (6.61;12.6)	30.15 (12.53;49.96)
Thailand	33034.11 (26017.54;45774.6)	24197.98 (18842.97;34030.55)	-26.75 (-37.92;-15.06)	20687.95 (15832.18;30760.02)	-37.37 (-47.59;-24.03)
Timor-Leste	268.64 (3.41;1541.92)	288.59 (3.17;1797.45)	7.43 (-44.62;82.46)	382.38 (4.33;2437.33)	42.34 (-32.37;134.74)
Vietnam	25260.55 (20775.86;32029.61)	20686.89 (16476.8;27769.37)	-18.11 (-26.84;-8.4)	25027.77 (20858.48;32476.09)	-0.92 (-8.83;9.55)
**Sub-Saharan Africa**					
**Central sub-Saharan Africa**					
Angola	27875.6 (21007.4;36549.02)	25237.52 (11583.66;41500.56)	-9.46 (-61.27;59.72)	28387.22 (11099.05;48307.06)	1.84 (-62.63;85.99)

Central African Republic	5375.9<br>(2693.68;7998.32)	5971.88<br>(1661.79;12525.6)	11.09<br>(-65.36;206.92)	6005.07<br>(1269.99;13156.51)	11.7<br>(-73.35;222.61)
Congo (Brazzaville)	7331.01<br>(5547.37;9269.6)	7802.97<br>(5645.37;10333.19)	6.44<br>(-10.93;29.48)	8450.57<br>(5946.17;11334.77)	15.27<br>(-5.12;40.96)
DR Congo	16313.3<br>(9425.15;24679.28)	20978.34<br>(6717.05;42792.91)	28.6<br>(-54.71;159.29)	22874.85<br>(4771.32;50352)	40.22<br>(-67.25;203.46)
Equatorial Guinea	4068.04<br>(2961.21;5352.5)	3137.32<br>(929.79;6967.85)	-22.88<br>(-76.19;48.97)	3517.91<br>(729.78;8286.9)	-13.52<br>(-82.89;75.7)
Gabon	1893.6<br>(1129.79;2692.7)	917.58<br>(527.79;1564.93)	-51.54<br>(-67.66;-18.59)	846.95<br>(459.92;1522.2)	-55.27<br>(-72.03;-18.37)
**Eastern sub-Saharan Africa**					
Burundi	1688.45<br>(904.95;2600.95)	4320.18<br>(1337.89;9466.01)	155.87<br>(6.76;374.28)	4929.05<br>(1117.97;11413.03)	191.93<br>(-7.44;462.54)
Comoros	2.26<br>(0.04;13.2)	14.83<br>(0.35;83)	556.83<br>(170.7;2699.69)	19.03<br>(0.45;109.56)	742.97<br>(263.85;3546.19)
Djibouti	631.2<br>(312.52;1093.07)	947.78<br>(291.97;1952.48)	50.16<br>(-51.8;211.18)	975.61<br>(285.92;2058.41)	54.56<br>(-55.25;227.94)
Eritrea	1402.44<br>(868.77;1990.56)	2040.76<br>(592.72;4089.85)	45.52<br>(-50.64;160.4)	2274.99<br>(485.07;4728.59)	62.22<br>(-61.32;201.8)
Ethiopia	16676.38<br>(12474.6;21795.93)	23861.18<br>(14992.83;35457.05)	43.08<br>(-6.7;105.46)	32122.11<br>(17464.23;51996.31)	92.62<br>(10.08;194.13)
Kenya	97329.12<br>(77874.06;121849.1)	117941.11<br>(82447.55;168301.16)	21.18<br>(-2.46;48.37)	170077.44<br>(118419.1;235557.58)	74.74<br>(39.65;112.01)
Madagascar	1829.45<br>(910.66;2868.18)	10738.61<br>(1290.95;50491.25)	486.99<br>(7.66;2150.32)	15633.23<br>(1936.29;68491.33)	754.53<br>(58.7;2966.81)
Malawi	54099.01<br>(43311.28;68026.25)	39440.94<br>(23051.66;58662.43)	-27.09<br>(-61.74;13.8)	39167.99<br>(18103.12;61856.1)	-27.6<br>(-69.76;21.18)
Mozambique	174428.18<br>(148563.34;207475.74)	135760.95<br>(88081.71;195694.4)	-22.17<br>(-49.89;9.15)	153643.69<br>(95867.82;223143.49)	-11.92<br>(-44.88;24.14)
Rwanda	11511.57<br>(9380.48;14129.89)	9157.47<br>(6287.61;13228.5)	-20.45<br>(-44.25;14.5)	9244.53<br>(5853.49;13839.34)	-19.69<br>(-48.3;21.92)
Somalia	2716.47<br>(292.78;10425.63)	3172.21<br>(167.74;12401.28)	16.78<br>(-67.87;127.6)	4772.81<br>(151.08;19357.46)	75.7<br>(-71.75;267.18)
South Sudan	7048.58<br>(580.75;22238.88)	4946.67<br>(200.45;18987.02)	-29.82<br>(-68.77;-7.67)	5482.27<br>(189.43;20659.83)	-22.22<br>(-71.91;6.1)
Tanzania	56959.9<br>(23074.26;98006.36)	62253.26<br>(6826.47;183294.35)	9.29<br>(-90.48;459.98)	67996.2<br>(195.75;216250.95)	19.38<br>(-99.74;558.38)
Uganda	114642.88<br>(79823.51;143136.91)	72704.12<br>(25610.84;201024.21)	-36.58<br>(-79.63;123.34)	72104.52<br>(11371.22;242617.42)	-37.11<br>(-90.91;168.17)
Zambia	58240.53<br>(46654.81;71666.5)	48738.09<br>(32026.12;71313.04)	-16.32<br>(-44.78;32.86)	55778.02<br>(35543.85;83732.01)	-4.23<br>(-39;53.99)
**Southern sub-Saharan Africa**					
Botswana	14942.83<br>(12126.86;18201.11)	9332.43<br>(6769.69;12328.06)	-37.55<br>(-56.12;-14.31)	9508.97<br>(6620.74;12982.67)	-36.36<br>(-56.08;-11.5)
Lesotho	21977.88<br>(18242.75;26872.17)	13837<br>(10151.79;18269.42)	-37.04<br>(-56.7;-16.35)	13427.37<br>(9476.91;18188.34)	-38.91<br>(-60;-17.85)
Namibia	11172.21<br>(8594.6;14324.07)	8455.25<br>(5098.24;13439.77)	-24.32<br>(-53.68;24.33)	8206.44<br>(5065.35;12615.66)	-26.55<br>(-55.23;16.54)
South Africa	413120.32<br>(339512.54;499977.23)	282117.95<br>(208107.68;354882.78)	-31.71<br>(-45.51;-17.59)	310443.92<br>(222835.31;397728.64)	-24.85<br>(-41.22;-7.3)
eSwatini	18083.28<br>(15525.67;21056.24)	2297.71<br>(505.17;4350.93)	-87.29***<br>(-97.32;-74.53)	2649.65<br>(348.32;5248.56)	-85.35<br>(-98.06;-69.8)
Zimbabwe	45748.57<br>(21577.72;82427.63)	69580.22<br>(7055.46;147350.05)	52.09<br>(-90.1;473.81)	79840.97<br>(2701.55;171853.29)	74.52<br>(-96.16;556.24)
**Western sub-Saharan Africa**					
Benin	4889.74<br>(3706.61;6312.8)	4628.01<br>(2797.38;6890.28)	-5.35<br>(-41.76;32.52)	5014.94<br>(2810.86;7735.06)	2.56<br>(-42.67;47.57)
Burkina Faso	2762.6<br>(1570.9;4234.03)	3718.54<br>(916.05;7261.27)	34.6<br>(-71.17;228.51)	3954.47<br>(388.76;8436.69)	43.14<br>(-87.01;283.52)
Cameroon	37334.82<br>(26045.26;49075.24)	34336.87<br>(11742.22;54204.83)	-8.03<br>(-68.43;52.25)	37272.77<br>(10268.66;61420.9)	-0.17<br>(-72.35;70.52)
Cape Verde	271.08<br>(192.7;373.5)	352.75<br>(250.57;493.58)	30.13<br>(12.73;48.12)	373.47<br>(264.23;519.31)	37.77<br>(19.01;56.58)
Chad	8524.89<br>(6089.51;11545.08)	11324.75<br>(4775.51;18928.19)	32.84<br>(-41.06;136.91)	13587.51<br>(4736.27;23626.55)	59.39<br>(-41.35;203.64)
Cote d'Ivoire	28740.77<br>(11762.28;46075.1)	51694.87<br>(10830.55;96546.72)	79.87<br>(-57.48;486.91)	56258.08<br>(10069.75;109083.7)	95.74<br>(-60.76;566.74)
The Gambia	1731.77<br>(1181.07;2447.8)	1444.23<br>(483.05;2498.88)	-16.6<br>(-69.03;36.08)	1692.97<br>(497.46;3018.48)	-2.24<br>(-69.12;65.5)
Ghana	16490.14<br>(11002.27;23528.55)	27602.33<br>(15963.36;44441.74)	67.39<br>(-7.3;193.13)	28610.55<br>(15774.51;46667.15)	73.5<br>(-7.65;205.79)
Guinea	7746.12<br>(5172.81;10289.67)	5980.35<br>(3206.27;9508.67)	-22.8<br>(-56.17;26.5)	7074.11<br>(3774.84;11417.9)	-8.68<br>(-48.57;50.32)
Guinea-Bissau	3155.29<br>(1992.61;4590.69)	2416.96<br>(858.45;4465.61)	-23.4<br>(-70.91;31.77)	2732.37<br>(910.16;5072.63)	-13.4<br>(-68;52.75)
Liberia	1571.46<br>(867.54;2433.43)	2857.24<br>(1300.98;4853.28)	81.82<br>(-9.37;235.47)	3197.81<br>(1372.51;5534.64)	103.49<br>(-3.14;288.36)
Mali	9021.4<br>(6774.81;11641.28)	8202.01<br>(5587.79;11126.39)	-9.08<br>(-26.39;9.88)	9834.86<br>(6517;13552.47)	9.02<br>(-13.48;34.29)
Mauritania	74.89<br>(2.05;446.36)	88.6<br>(2.52;476.49)	18.32<br>(-30.09;130.28)	130.22<br>(3.88;727.33)	73.9<br>(3.12;237.81)
Niger	1670.51<br>(1030.78;2404.91)	2842.7<br>(1711.92;4265.28)	70.17<br>(16.55;149.33)	3439.28<br>(1897.61;5364.43)	105.88<br>(32.69;206.14)
Nigeria	289543.2<br>(228393.04;359264.02)	227542.58<br>(164431.42;296715.1)	-21.41<br>(-36.24;-0.65)	268159.46<br>(189389.91;355042.92)	-7.39<br>(-26.36;19.12)
Sao Tome and Principe	1.07<br>(0.29;2.37)	1.78<br>(0.45;3.91)	66.74<br>(-32.52;317.13)	2.87<br>(0.71;6.02)	168.39<br>(10.42;556.15)

Senegal	3344.13 (2215.28;4636.23)	3909.01 (2411.47;5804.78)	16.89 (-14.21;79.34)	6209.05 (3723.11;9346.78)	85.67 (34.07;192.45)
Sierra Leone	3547.09 (1548.15;5670.55)	3596.68 (656.84;7801.15)	1.4 (-82.61;196.17)	3918.43 (424.08;8764.71)	10.47 (-89.08;233.54)
Togo	5779.33 (3608.28;8287.42)	4796.52 (1340.81;9423.25)	-17.01 (-76.91;78.36)	4886.15 (731.53;10306.72)	-15.45 (-87.29;93.92)

***Meets Target
UI = Uncertainty Interval

Appendix Figure 1. Percentage of PLHIV on and off ART coverage in all GBD super−regions, by country, by sex, 2017
Latin America and Caribbean

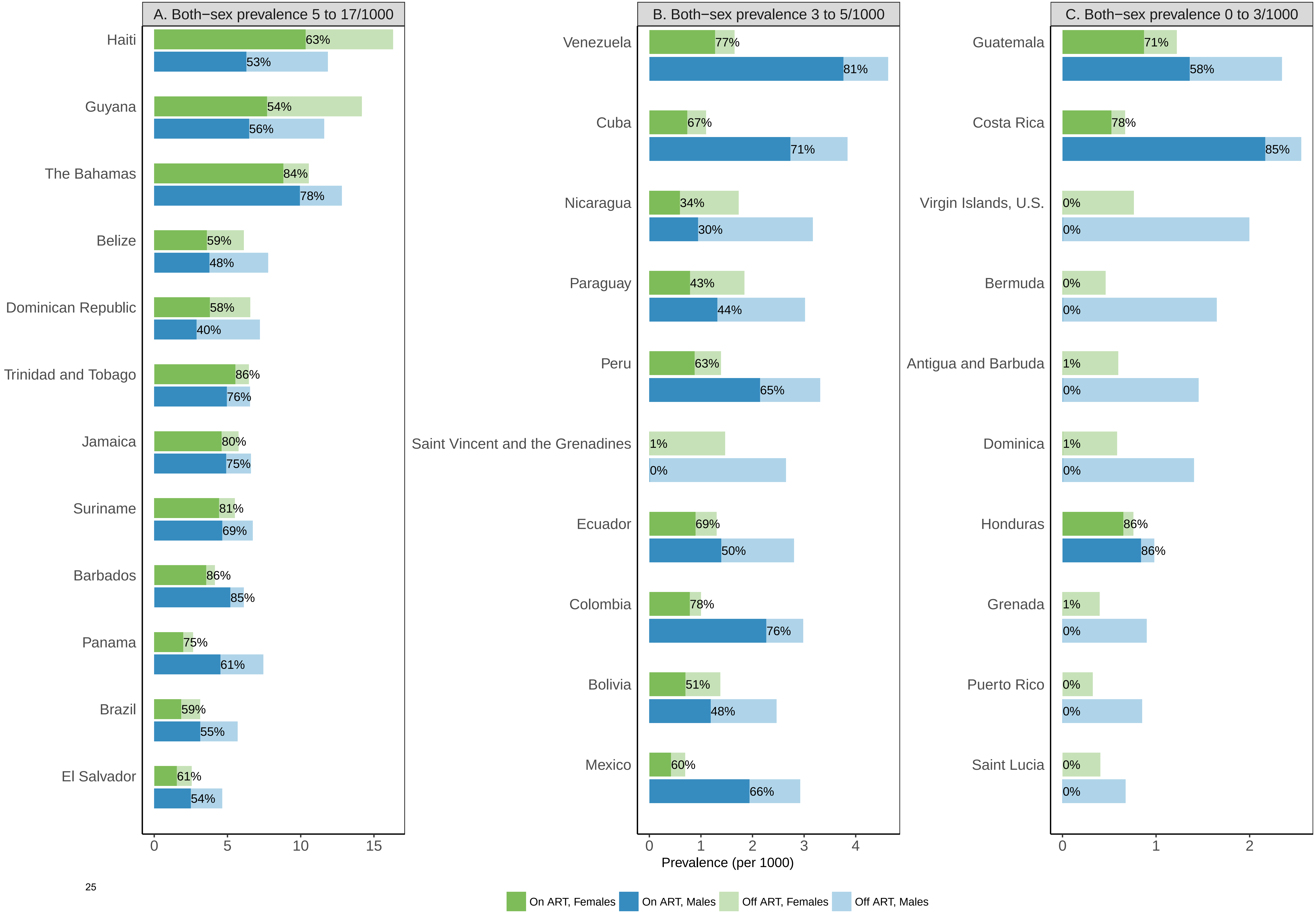

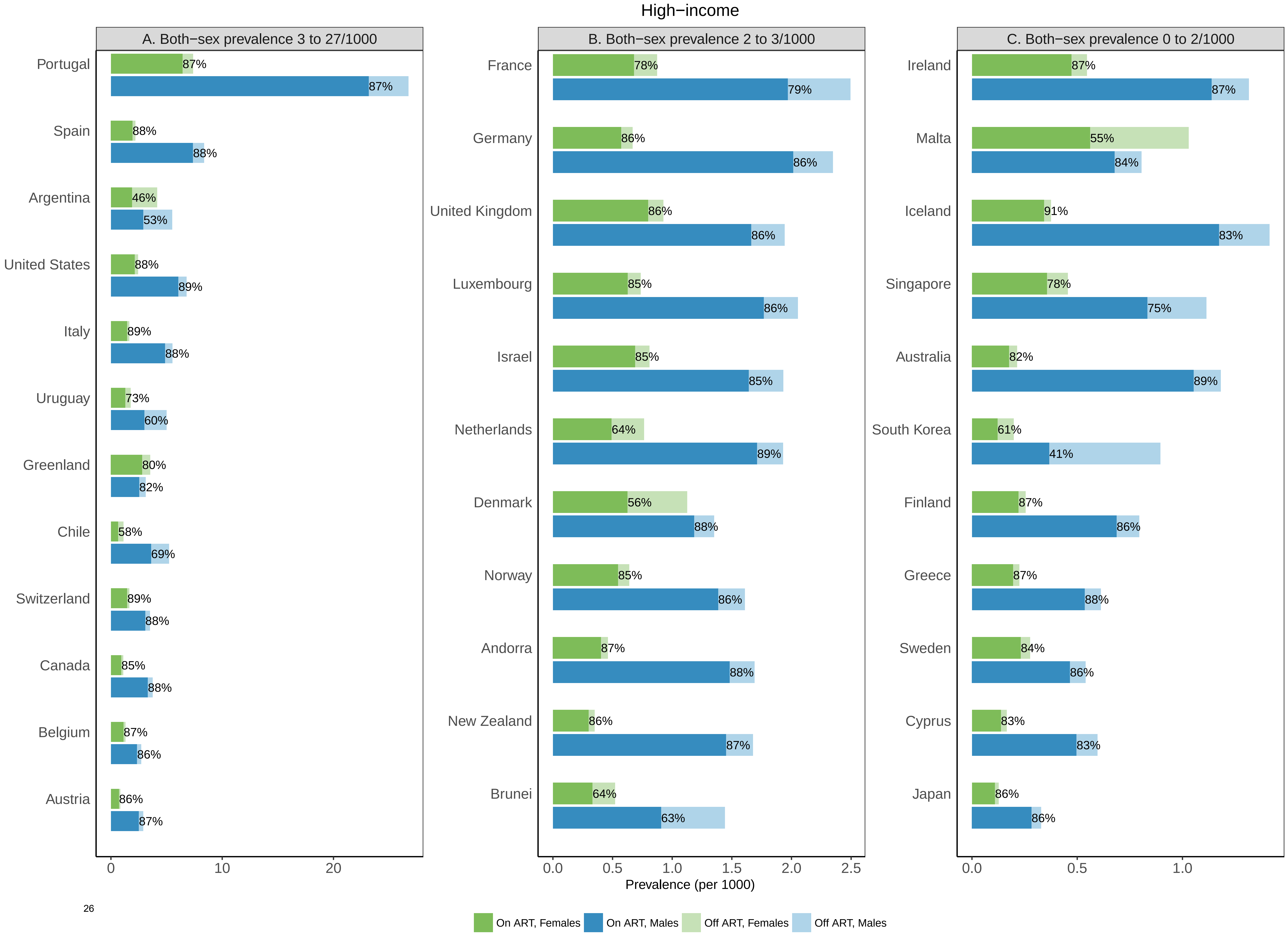
High−income
A. Both−sex prevalence 3 to 27/1000
B. Both−sex prevalence 2 to 3/1000
C. Both−sex prevalence 0 to 2/1000
Portugal 87% 87%
Spain 88% 88%
Argentina 46% 53%
United States 88% 89%
Italy 89% 88%
Uruguay 73% 60%
Greenland 80% 82%
Chile 58% 69%
Switzerland 89% 88%
Canada 85% 88%
Belgium 87% 86%
Austria 86% 87%
France 78% 79%
Germany 86% 86%
United Kingdom 86% 86%
Luxembourg 85% 86%
Israel 85% 85%
Netherlands 64% 89%
Denmark 56% 88%
Norway 85% 86%
Andorra 87% 88%
New Zealand 86% 87%
Brunei 64% 63%
Ireland 87% 87%
Malta 55% 84%
Iceland 91% 83%
Singapore 78% 75%
Australia 82% 89%
South Korea 61% 41%
Finland 87% 86%
Greece 87% 88%
Sweden 84% 86%
Cyprus 83% 83%
Japan 86% 86%
0 10 20
0.0 0.5 1.0 1.5 2.0 2.5
0.0 0.5 1.0
Prevalence (per 1000)
On ART, Females
On ART, Males
Off ART, Females
Off ART, Males

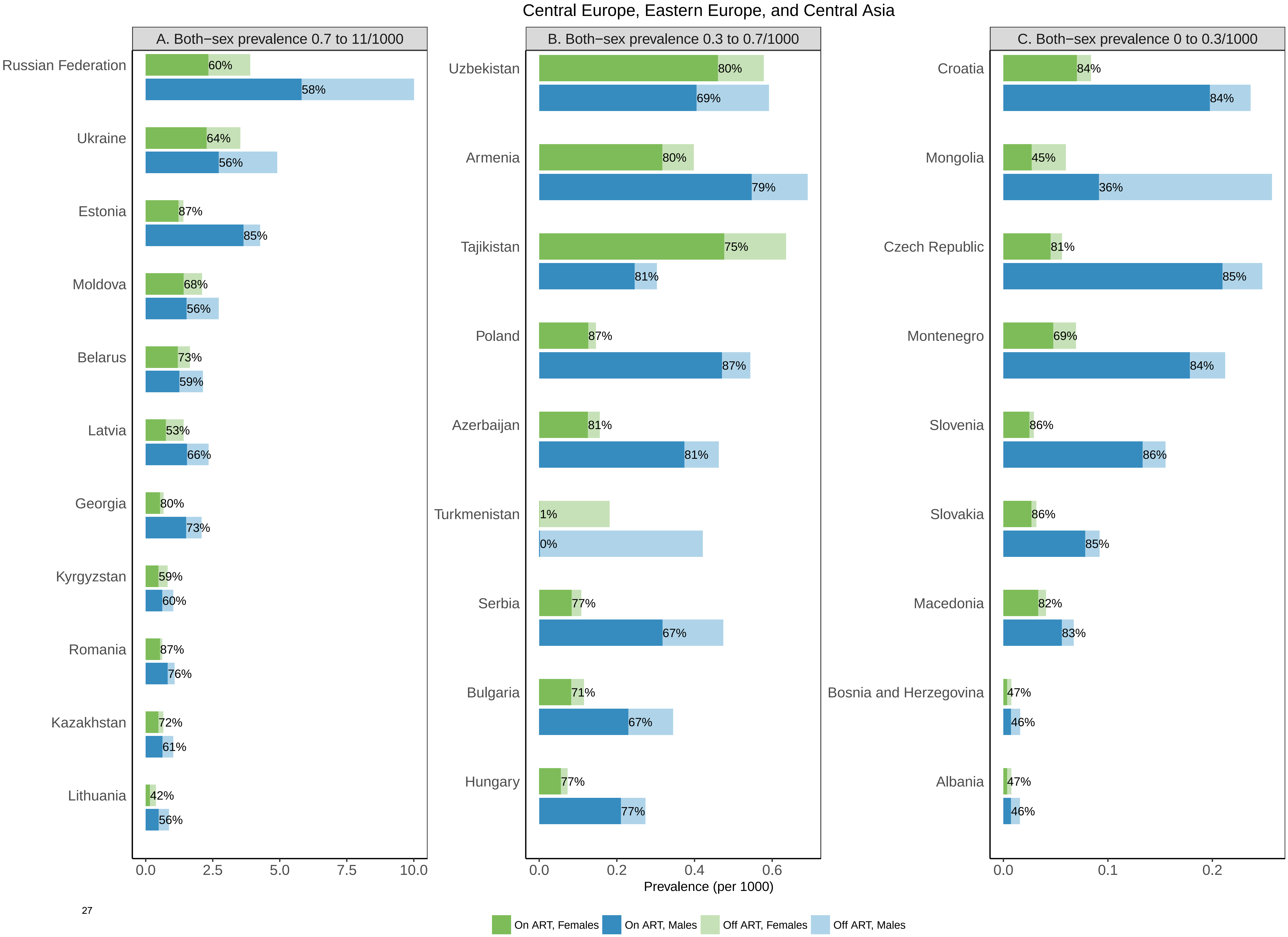

Central Europe, Eastern Europe, and Central Asia
A. Both−sex prevalence 0.7 to 11/1000
B. Both−sex prevalence 0.3 to 0.7/1000
C. Both−sex prevalence 0 to 0.3/1000
Russian Federation 60% 58%
Ukraine 64% 56%
Estonia 87% 85%
Moldova 68% 56%
Belarus 73% 59%
Latvia 53% 66%
Georgia 80% 73%
Kyrgyzstan 59% 60%
Romania 87% 76%
Kazakhstan 72% 61%
Lithuania 42% 56%
Uzbekistan 80% 69%
Armenia 80% 79%
Tajikistan 75% 81%
Poland 87% 87%
Azerbaijan 81% 81%
Turkmenistan 1% 0%
Serbia 77% 67%
Bulgaria 71% 67%
Hungary 77% 77%
Croatia 84% 84%
Mongolia 45% 36%
Czech Republic 81% 85%
Montenegro 69% 84%
Slovenia 86% 86%
Slovakia 86% 85%
Macedonia 82% 83%
Bosnia and Herzegovina 47% 46%
Albania 47% 46%
0.0 2.5 5.0 7.5 10.0
0.0 0.2 0.4 0.6
0.0 0.1 0.2
Prevalence (per 1000)
On ART, Females
On ART, Males
Off ART, Females
Off ART, Males

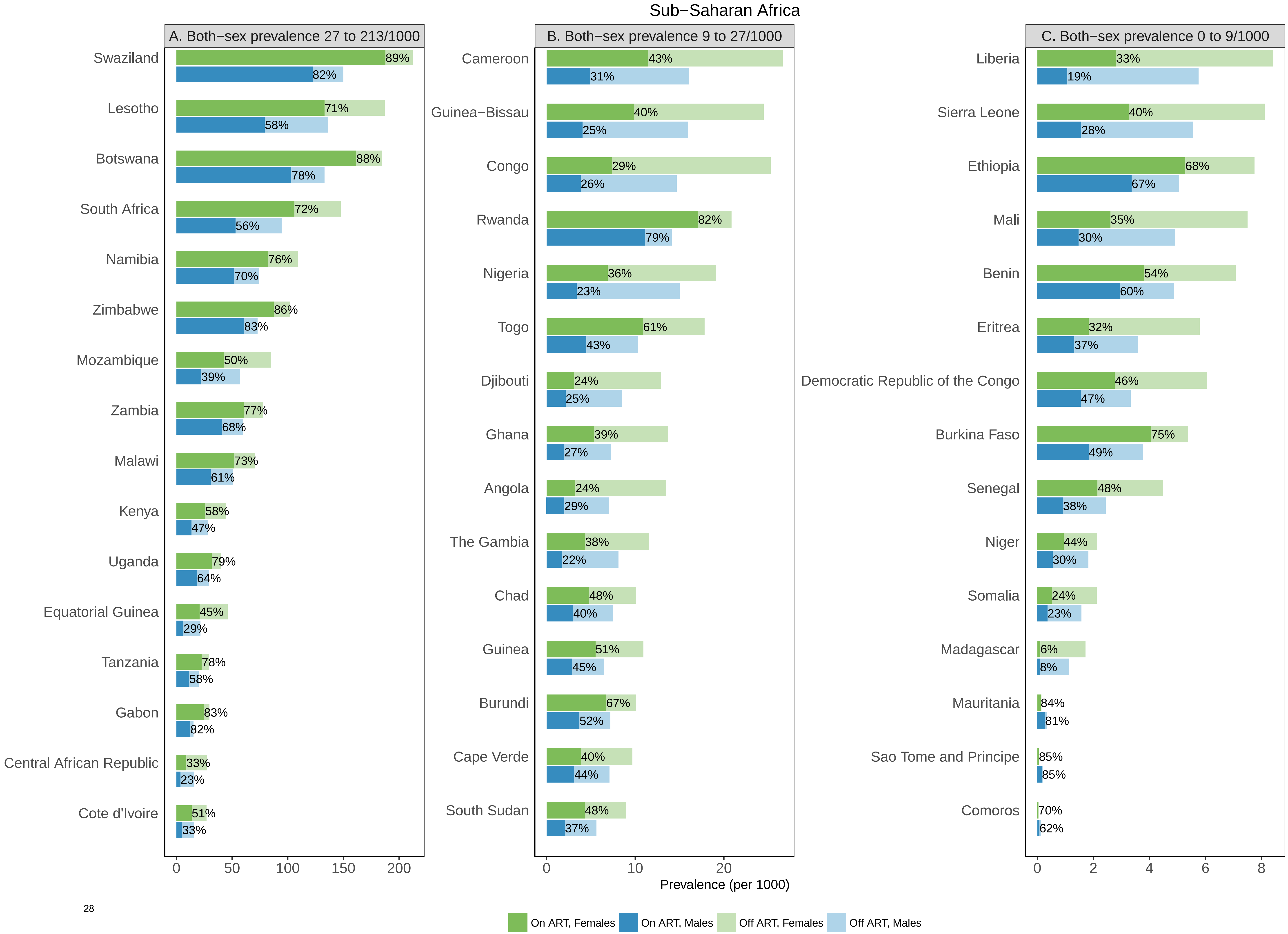

Sub−Saharan Africa
A. Both−sex prevalence 27 to 213/1000
B. Both−sex prevalence 9 to 27/1000
C. Both−sex prevalence 0 to 9/1000
Swaziland 89% 82%
Lesotho 71% 58%
Botswana 88% 78%
South Africa 72% 56%
Namibia 76% 70%
Zimbabwe 86% 83%
Mozambique 50% 39%
Zambia 77% 68%
Malawi 73% 61%
Kenya 58% 47%
Uganda 79% 64%
Equatorial Guinea 45% 29%
Tanzania 78% 58%
Gabon 83% 82%
Central African Republic 33% 23%
Cote d'Ivoire 51% 33%
Cameroon 43% 31%
Guinea−Bissau 40% 25%
Congo 29% 26%
Rwanda 82% 79%
Nigeria 36% 23%
Togo 61% 43%
Djibouti 24% 25%
Ghana 39% 27%
Angola 24% 29%
The Gambia 38% 22%
Chad 48% 40%
Guinea 51% 45%
Burundi 67% 52%
Cape Verde 40% 44%
South Sudan 48% 37%
Liberia 33% 19%
Sierra Leone 40% 28%
Ethiopia 68% 67%
Mali 35% 30%
Benin 54% 60%
Eritrea 32% 37%
Democratic Republic of the Congo 46% 47%
Burkina Faso 75% 49%
Senegal 48% 38%
Niger 44% 30%
Somalia 24% 23%
Madagascar 6% 8%
Mauritania 84% 81%
Sao Tome and Principe 85% 85%
Comoros 70% 62%
0 50 100 150 200
0 10 20
0 2 4 6 8
Prevalence (per 1000)
On ART, Females
On ART, Males
Off ART, Females
Off ART, Males

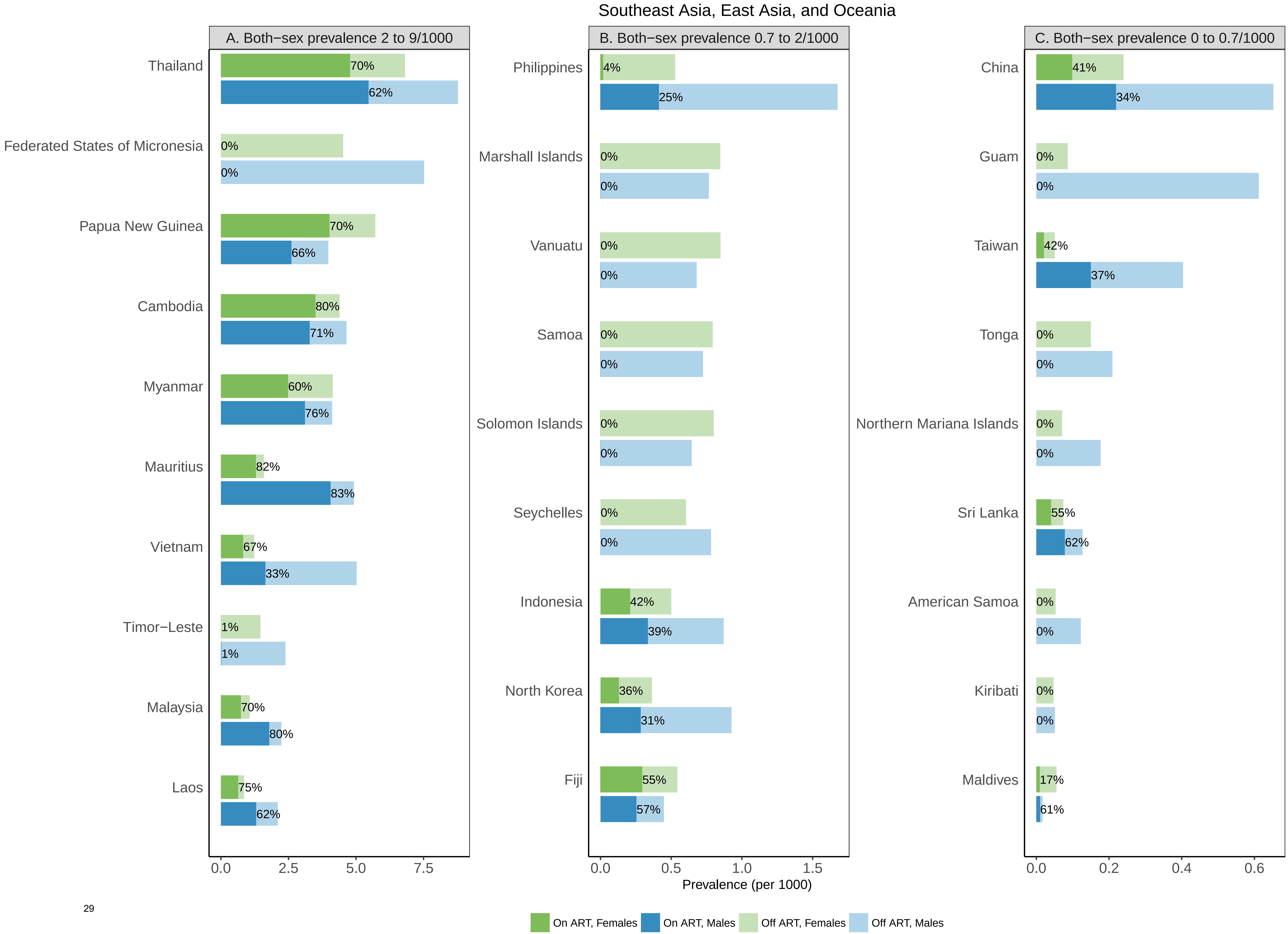
Southeast Asia, East Asia, and Oceania
A. Both−sex prevalence 2 to 9/1000
B. Both−sex prevalence 0.7 to 2/1000
C. Both−sex prevalence 0 to 0.7/1000
Thailand 70% 62%
Federated States of Micronesia 0% 0%
Papua New Guinea 70% 66%
Cambodia 80% 71%
Myanmar 60% 76%
Mauritius 82% 83%
Vietnam 67% 33%
Timor−Leste 1% 1%
Malaysia 70% 80%
Laos 75% 62%
Philippines 4% 25%
Marshall Islands 0% 0%
Vanuatu 0% 0%
Samoa 0% 0%
Solomon Islands 0% 0%
Seychelles 0% 0%
Indonesia 42% 39%
North Korea 36% 31%
Fiji 55% 57%
China 41% 34%
Guam 0% 0%
Taiwan 42% 37%
Tonga 0% 0%
Northern Mariana Islands 0% 0%
Sri Lanka 55% 62%
American Samoa 0% 0%
Kiribati 0% 0%
Maldives 17% 61%
0.0 2.5 5.0 7.5
0.0 0.5 1.0 1.5
0.0 0.2 0.4 0.6
Prevalence (per 1000)
On ART, Females
On ART, Males
Off ART, Females
Off ART, Males

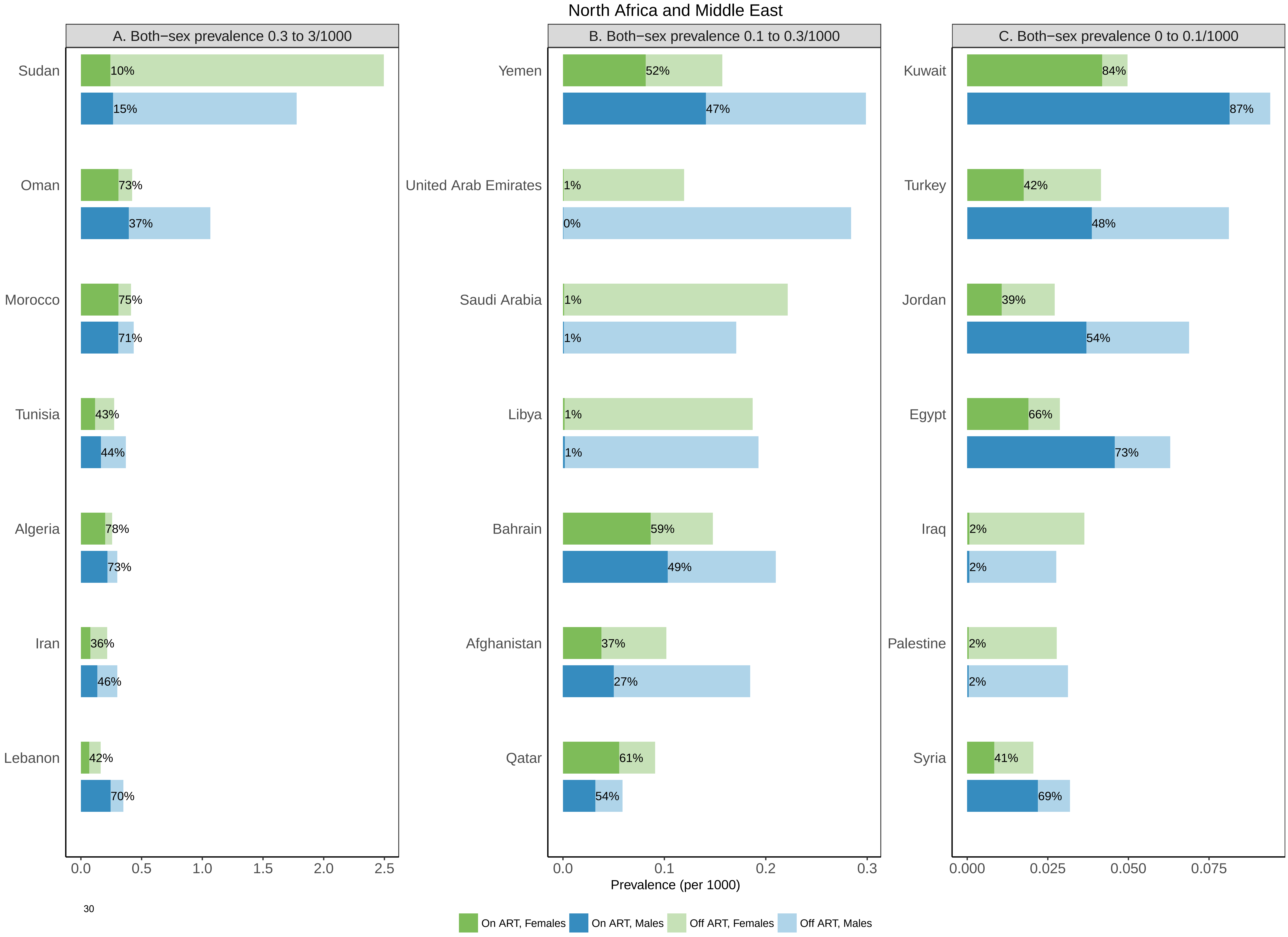
North Africa and Middle East
A. Both−sex prevalence 0.3 to 3/1000
Sudan
10%
15%
Oman
73%
37%
Morocco
75%
71%
Tunisia
43%
44%
Algeria
78%
73%
Iran
36%
46%
Lebanon
42%
70%
0.0
0.5
1.0
1.5
2.0
2.5
B. Both−sex prevalence 0.1 to 0.3/1000
Yemen
52%
47%
United Arab Emirates
1%
0%
Saudi Arabia
1%
1%
Libya
1%
1%
Bahrain
59%
49%
Afghanistan
37%
27%
Qatar
61%
54%
0.0
0.1
0.2
0.3
Prevalence (per 1000)
C. Both−sex prevalence 0 to 0.1/1000
Kuwait
84%
87%
Turkey
42%
48%
Jordan
39%
54%
Egypt
66%
73%
Iraq
2%
2%
Palestine
2%
2%
Syria
41%
69%
0.000
0.025
0.050
0.075
On ART, Females
On ART, Males
Off ART, Females
Off ART, Males

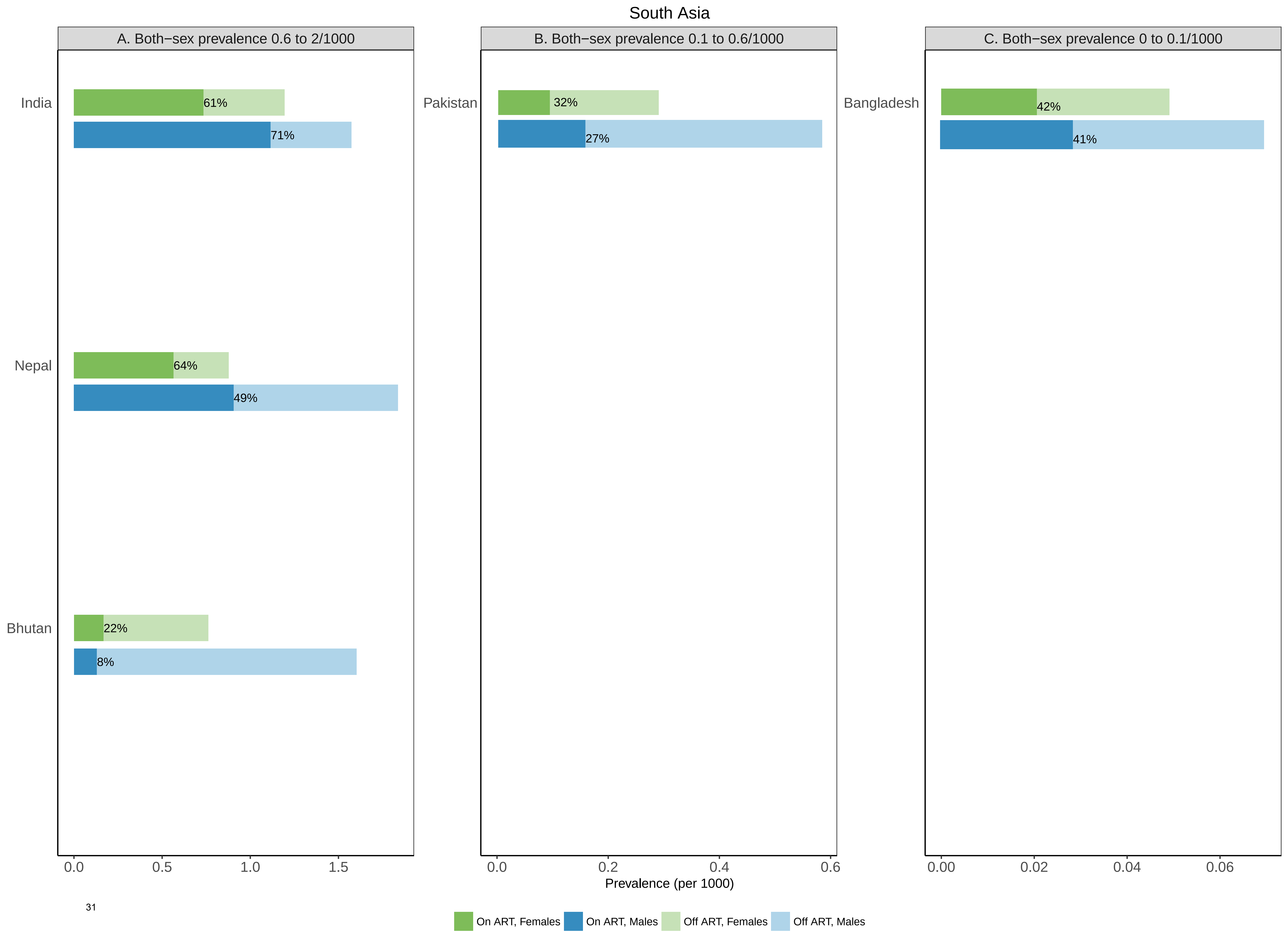
South Asia
A. Both−sex prevalence 0.6 to 2/1000
B. Both−sex prevalence 0.1 to 0.6/1000
C. Both−sex prevalence 0 to 0.1/1000
India
61%
71%
Nepal
64%
49%
Bhutan
22%
8%
Pakistan
32%
27%
Bangladesh
42%
41%
0.0
0.5
1.0
1.5
0.0
0.2
0.4
0.6
0.00
0.02
0.04
0.06
Prevalence (per 1000)
On ART, Females
On ART, Males
Off ART, Females
Off ART, Males

Appendix Figure 2. All Age HIV incidence (A), prevalence (B), and mortality (C), in 2017, for both sexes.

A) Incidence

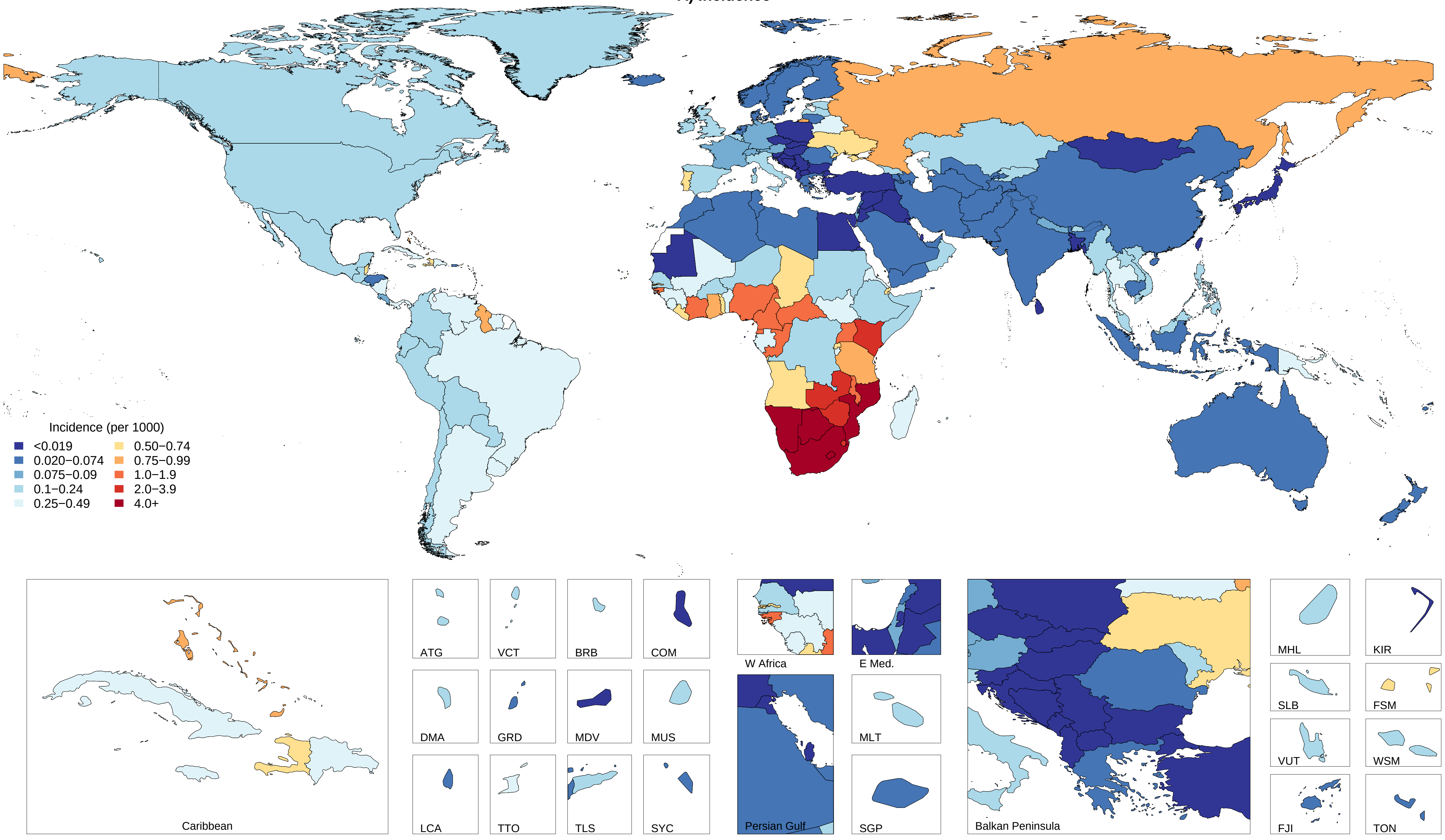

B) Prevalence

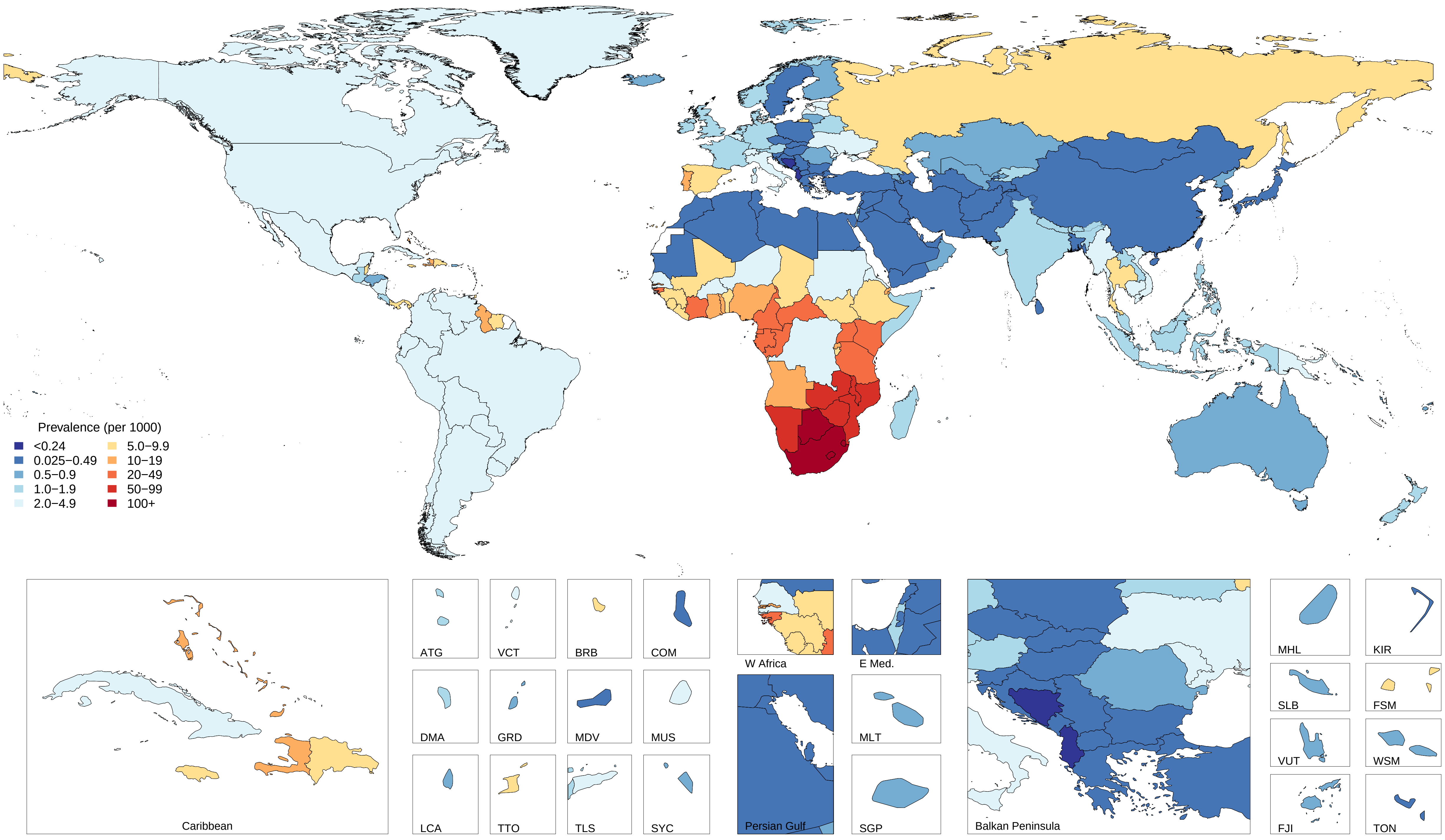

C) Mortality

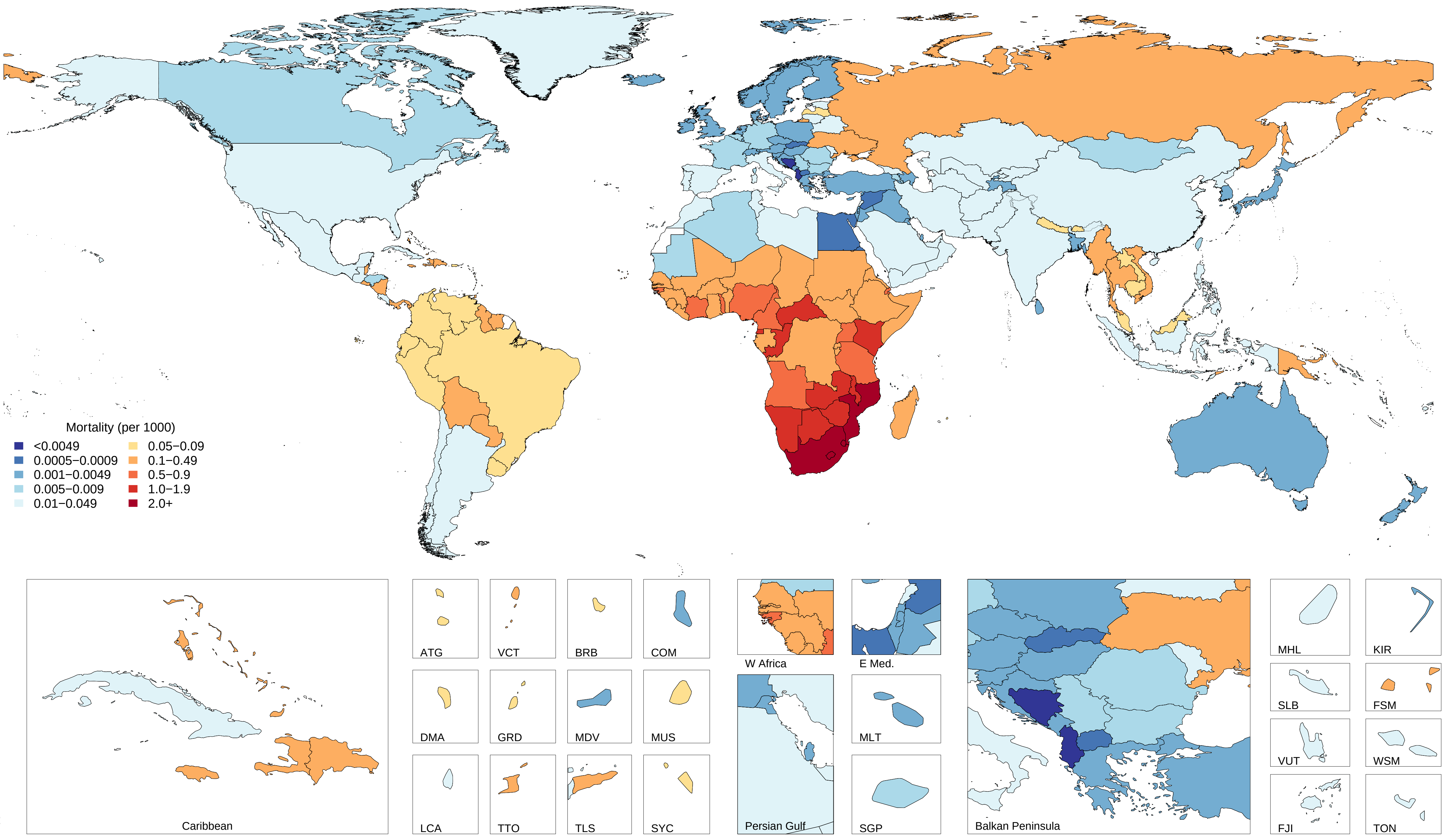

Appendix Figure 3. Percentage of new HIV infections and HIV deaths occurring in each age group in Sub−Saharan Africa, by sex, 2007 and 2017

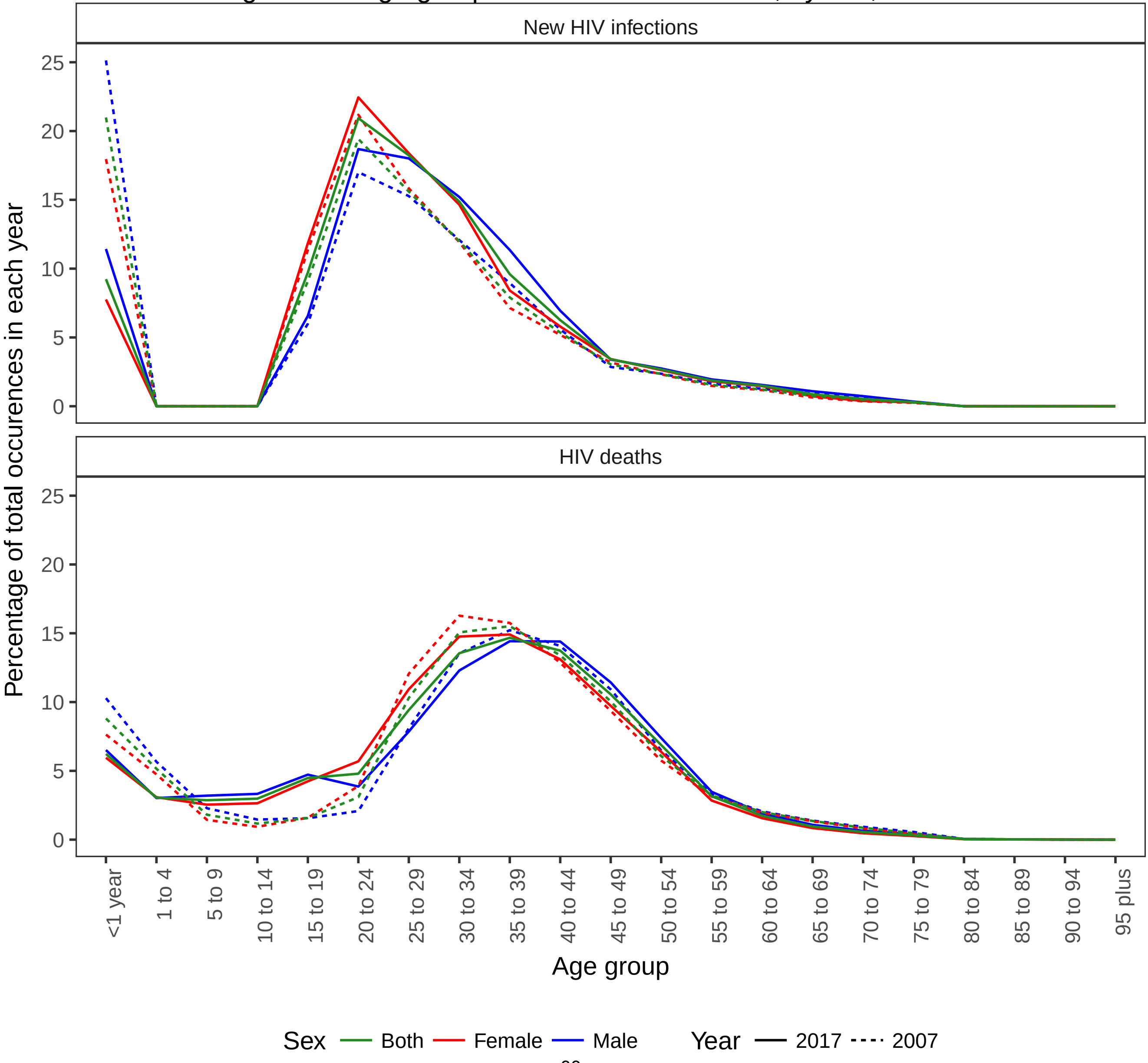

Supplement: Supplementary appendix 2 [file mmc2.pdf]
